# A label-free method for measuring the composition of multicomponent biomolecular condensates

**DOI:** 10.1038/s41557-025-01928-3

**Published:** 2025-09-03

**Authors:** Patrick M. McCall, Kyoohyun Kim, Anna Shevchenko, Martine Ruer-Gruß, Jan Peychl, Jochen Guck, Andrej Shevchenko, Anthony A. Hyman, Jan Brugués

**Affiliations:** 1 Max Planck Institute of Molecular Cell Biology and Genetics, Dresden, Germany; 2 Max Planck Institute for the Physics of Complex Systems, Dresden, Germany; 3 Center for Systems Biology Dresden, Dresden, Germany; 4 Cluster of Excellence Physics of Life, Technische Universität Dresden, Dresden, Germany; 5 Leibniz Institute of Polymer Research Dresden, Dresden, Germany; 6 Biotechnology Center, Technische Universität Dresden, Dresden, Germany; 7 Max Planck Institute for the Science of Light, Erlangen, Germany

**Keywords:** Thermodynamics, Organelles, RNA-binding proteins, Phase-contrast microscopy, Bioanalytical chemistry

## Abstract

Many subcellular compartments are biomolecular condensates made of multiple components, often including several distinct proteins and nucleic acids. However, current tools to measure condensate composition are limited and cannot capture this complexity quantitatively because they either require fluorescent labels, which can perturb composition, or can distinguish only one or two components. Here we describe a label-free method based on quantitative phase imaging and analysis of tie-lines and refractive index to measure the composition of reconstituted condensates with multiple components. We first validate the method empirically in binary mixtures, revealing sequence-encoded density variation and complex ageing dynamics for condensates composed of full-length proteins. We then use analysis of tie-lines and refractive index to simultaneously resolve the concentrations of five macromolecular solutes in multicomponent condensates containing RNA and constructs of multiple RNA-binding proteins. Our measurements reveal an unexpected decoupling of density and composition, highlighting the need to determine molecular stoichiometry in multicomponent condensates. We foresee this approach enabling the study of compositional regulation of condensate properties and function.

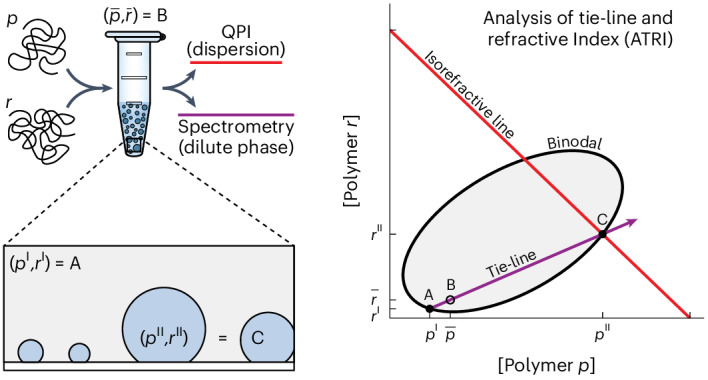

## Abstract (web summary)

Biomolecular condensates are made of multiple components but current techniques cannot capture their complex composition quantitatively. Now it has been shown that the dense-phase binodal point defining the composition of multicomponent condensates can be inferred precisely from the intersection of a spectrometrically determined tie-line with an isorefractive line obtained from quantitative phase imaging.

## Main

**Fig. 1:**
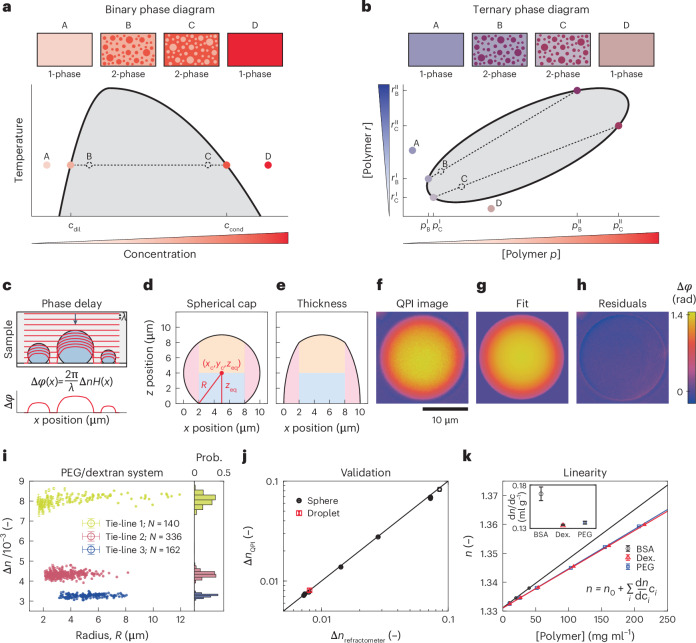
QPI measures the refractive index and shape of demixed micrometre-sized droplets. **a**, Phase diagram in a prototypical binary mixture with sketches of samples prepared in the one-phase (A,D) and two-phase regimes (B,C). Compositions of coexisting phases, *c*_dil_ and *c*_cond_, lie on the binodal curve (solid black) that separates the one-phase (mixed) and two-phase (demixed) regimes. A tie-line (dashed black) connects compositions of coexisting phases to the corresponding average composition (open dashed black circle). Varying average solute concentration in the two-phase regime (grey) changes the relative volumes of coexisting phases but not their composition. **b**, Phase diagram in a prototypical ternary mixture with sketches of samples prepared at different average compositions. Unlike the binary case, ternary mixtures prepared at different points in the two-phase regime may lie on different tie-lines, yielding compositionally distinct pairs of coexisting phases. **c**, Schematic of optical wavefronts (red) distorted by droplets on a flat surface (top) and the cumulative optical phase shift (bottom). **d**, Schematic defining the geometry of a spherical cap. **e**, Local thickness profile *H*(*x*) for the geometry in **d**. Shading in **d**,**e** denotes separate terms in the analytic expression for *H*(*x*) (Methods). **f**, Phase image of dextran-rich droplet on passivated glass. **g**, Fit of droplet in **f** to spherical cap. **h**, Residuals from fit. Scale bar in **f**–**h**, 10 µm. **i**, Refractive index difference ∆*n* versus droplet radius *R* extracted from fits to individual dextran-rich droplets obtained from PEG/dextran mixtures prepared on three different tie-lines (colours). Error bars are 95% confidence intervals from fits. Probability histograms at right. **j**, Mean refractive index differences measured with QPI versus bulk refractometry for PEG/dextran mixtures (red) or silica microspheres in different glycerol–water mixtures (black). Error bars in *x* are the s.d. of *N* = 5 repeat measurements. Error bars in *y* are the s.d. from a population of objects in a single sample. For the PEG/dextran system, *N* = 140 droplets on tie-line 1. In order of increasing Δ*n*_QPI_ for the silica bead system, *N* = 62, 71, 92, 77, 57, 7, 16, 8, 10, 56, 63 or 21 microspheres. Solid line is *y* = *x*. **k**, Refractive index is a linear function of component concentration for three model biopolymers (BSA, dextran, PEG). Data points represent mean ± s.d. of *N* = 3 repeats. Inset: slopes (d*n/*d*c*) for each polymer. Error bars represent 95% confidence intervals from the linear fits in the main panel. Source data

Many compartments in living cells exist as condensed phases of biopolymers, termed biomolecular condensates, which are demixed from the surrounding cytoplasm or nucleoplasm^1,2^, and are implicated in a wide range of cellular processes^3^. Phase separation of a simple binary mixture of a polymer in solvent results in a dilute phase coexisting with a polymer-rich condensed phase (Fig. 1a). Although demixing of a single full-length protein in a binary mixture is often sufficient to reconstitute simplified condensates^4–7^, condensates in vivo contain dozens of components^8–10^. Indeed, the functional identity of a particular condensate inside a cell is determined by its composition. Unlike binary systems, such multicomponent condensates possess a continuum of compositions^11^, each connected to a coexisting dilute phase via a tie-line in a phase diagram (Fig. 1b). Changes in component abundance can thus shift the system to a new tie-line, altering condensate composition and physical properties^12,13^. Despite its central role in physically defining condensates and specifying their properties, the composition of multicomponent condensates in vivo, and component stoichiometries in reconstituted systems in vitro, are largely unknown.

In multicomponent systems, condensate composition is typically estimated by fusing each molecular species to a spectrally distinct fluorescent tag^12,14,15^. Although this approach is powerful, fluorescent tags contribute to interactions between species and with the solvent, potentially shifting the thermodynamic balance that specifies phase composition^16,17^. Interactions with the condensed phase can also drive strong deviations in fluorophore characteristics relative to behaviour calibrated in the dilute phase^15,18,19^, confounding quantification. Label-free techniques avoid these issues entirely, but existing approaches require harsh treatments^20–23^ and are effectively limited to binary systems for native-like molecules^24–28^ (Supplementary Notes 1 (refs. ^19–28^) and 2 (refs. ^6,29–32^)).

Reflecting these limitations, recent ultraviolet (UV) absorption measurements of the composition of condensates reconstituted from recombinant proteins looked exclusively at binary systems containing intrinsically disordered protein regions (IDRs) rather than the full-length proteins^29–31^. This is largely because the former can be purified with sufficient yield from bacteria following denaturation. However, in many cases, producing proteins in bacteria is not an option because bacteria lack the machinery needed to add post-translational modifications (PTMs) or assist folding of certain protein domains. Furthermore, denaturants may irreversibly alter protein conformational ensembles. Thus, it remains unclear how the additional features of native-like proteins obtainable from eukaryotic expression systems, including PTMs, native-like conformational ensembles, and the additional domains present in full-length proteins, will change the picture now emerging for condensates formed from IDRs alone. As functional roles for PTMs and structured protein domains accumulate alongside molecular parts lists^8–10^, there is a pressing need for methods to reveal condensate composition in more faithful reconstitutions that include multiple native-like components.

In this work, we present a label-free method to precisely measure the composition of micrometre-sized condensates reconstituted from multiple components. Utilizing quantitative phase imaging (QPI), this method requires 1,000-fold less material than bulk label-free alternatives, which enables dynamic and temperature-dependent measurements of condensates formed from full-length native-like components. We first validate an analysis procedure for QPI that extracts the refractive index difference Δ*n* between micrometre-sized droplets and the surrounding solution with improved precision relative to existing tomography-based approaches^27^. For binary systems, we find that the protein sequence, and the use and choice of labels influence dense-phase concentrations and uncover an increase in density with age that may underlie previous reports of time-dependent mechanical properties^33^. We then show that the concentrations of many individual species in multicomponent condensates can be resolved quantitatively by combining Δ*n* with tie-line measurements in an analysis of tie-lines and refractive index (ATRI). We demonstrate this explicitly for model condensates containing RNA and up to four distinct RNA-binding-protein constructs. By resolving the chemical composition of multicomponent condensed phases in situ and in unprecedented detail, we anticipate that this label-free method will enable mechanistic studies of complex compositional regulation of biomolecular condensate properties and function with many native components.

## Results

### Validating droplet refractive index differences from QPI

To measure the compositional difference between micrometre-sized droplets and the coexisting dilute phase, we use QPI^34,35^. Physically, QPI measures the optical phase shift accumulated along a wavefront as it traverses spatial inhomogeneities in the refractive index within a sample, such as high-refractive index droplets immersed in a lower-index medium (Fig. 1c). Within the straight-line optical path approximation (Supplementary Fig. 1, and Supplementary Note 3), the optical phase shift Δ*φ* measured by QPI at pixel $\left(x,y\right)$ is proportional to the product of the refractive index difference, Δ*n*, and the droplet shape

1 $$\Delta \varphi \left(x,y\right)=\frac{2\uppi }{\lambda }\Delta {nH}\left(x,y\right),$$

where *λ* is the imaging wavelength and *H*(*x*,*y*) is the local thickness of the droplet along the imaging axis. Thus, the refractive index difference between coexisting thermodynamic phases can be obtained from the droplet’s shape and optical phase shift.

**Extended Data Fig. 1:**
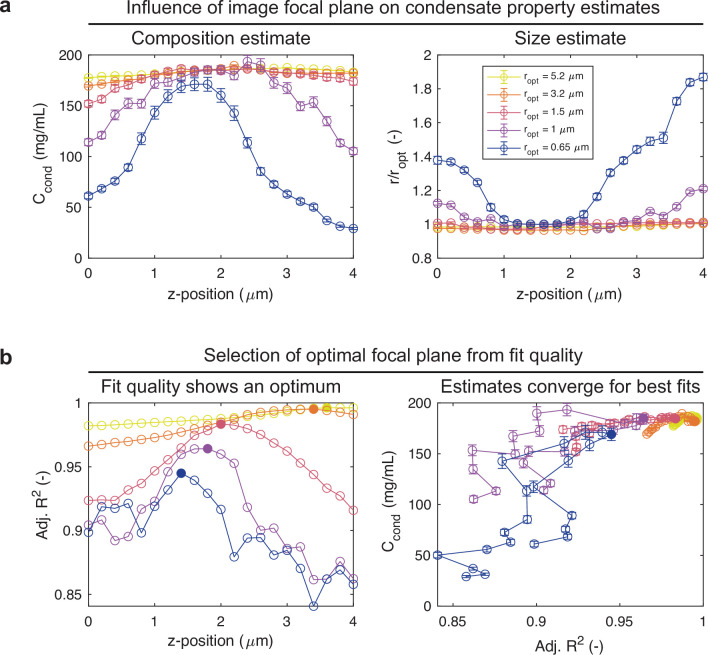
Fit quality guides optimal image plane selection for QPI analysis. Estimates of condensate properties from QPI data vary non-monotonically with z-position of the focal plane, displaying size-dependent extrema. In order to obtain suitable data on a polydisperse droplet population, QPI images are acquired in z-stacks with a distance of 0.2 µm between consecutive images. **a**, Influence of image focal plane on estimates of dense-phase protein concentration (left) and radius (right) for aged PGL-3 condensates of different sizes (colours) in contact with a coverslip. Property estimates are obtained by fitting condensate images in each plane of the z-stack independently. Estimates vary weakly with z-position for large droplets but strongly for small ones. **b**, For each condensate, the quality of the fit obtained in each image varies non-monotonically with focal plane and displays a maximum near the droplet equatorial plane (left, solid circles). Note that z = 0 corresponds to a focal plane somewhat below the coverslip surface. Plotted as a function of fit quality (right), property estimates from individual images across multiple droplets converge to a consistent value for the highest Adj. R^2^ values. For each condensate, we retain the estimate from only the image plane in which the Adj. R^2^-value for its fit was greatest. To ensure accuracy, condensates whose highest Adj. R^2^-value lie below a threshold (typically 0.95 to 0.98) are discarded. Using this protocol, the 0.65-µm condensate (blue), whose best concentration estimate lies below those of the larger condensates, is discarded. Each coloured trace corresponds to an individual droplet. Error bars represent the uncertainty in the fit to the image acquired at the indicated z-position. Source data

For proteins, and for many other solutes relevant for molecular biology experiments, the refractive index of an aqueous solution is well described as a linear function of concentration over a wide range^36–38^ (Supplementary Figs. 2 and 3, and Supplementary Note 4 (refs. ^36–43^)). In the linear approximation (Supplementary Fig. 4), the refractive index difference between two phases with *N* solutes is

2 $$\Delta n\approx\mathop{\sum}\limits_{i=1}^{N}\frac{{\mathrm{d}n}}{\mathrm{d}{c}_{i}}{\Delta c}_{i},$$

where Δ*c*_*i*_ is the concentration difference of the *i*th component between the two phases. For a binary mixture, *N* = 1 and the sum contains only a single term. The condensed-phase protein concentration, *c*_cond_, in a binary system is therefore given by

3 $${c}_{\mathrm{cond}}=\frac{\Delta n}{{\mathrm{d}n}/{\mathrm{d}c}}+{c}_{\mathrm{dil}},$$

where *c*_dil_ is the dilute phase concentration and d*n*/d*c* is the slope of the concentration dependence of the refractive index, which can be estimated from the amino acid sequence^32^. This suggests that QPI is suitable for measuring the concentration difference between a condensate and its coexisting dilute phase in a binary system when the condensate shape is known. The shape of a sessile droplet on a flat substrate is well captured by a spherical cap (Fig. 1d) for droplets smaller than the capillary length^44^, which we estimate as typically ≳3 µm for reconstituted condensates (Supplementary Fig. 5 and Supplementary Note 5 (ref. ^44^)). We therefore extract the condensate refractive index and shape parameters from QPI images by fitting (Fig. 1e, Methods and Extended Data Fig. 1).

To validate this QPI approach experimentally, we use two different reference systems. The first is a well-characterized dextran/polyethylene glycol (PEG) aqueous two-phase system^45^ for which we can readily measure Δ*n* independently by bulk refractometry (Methods). QPI images of dextran-rich droplets on a passivated coverglass and surrounded by a coexisting PEG-rich phase (Fig. 1f) are well modelled as spherical caps (Fig. 1g), as evidenced by small and spatially unstructured residuals in the droplet interior following fitting (Fig. 1h). For the best fits, Δ*n* is independent of size for three different PEG/dextran compositions (Fig. 1i, left), and is approximately symmetrically distributed (Fig. 1i, right), indicative of equilibrated phases and uncertainty dominated by statistics rather than systematics, respectively. Crucially, the Δ*n* values extracted from femtolitre-droplets in QPI images are in excellent agreement with those measured independently from 100-µl volumes of each phase using a digital refractometer (Fig. 1j). To validate application of the straight-line optical path approximation for larger Δ*n*, we used silica microspheres suspended in glycerol–water mixtures as a second reference system, where Δ*n* is set by the glycerol/water ratio. As with the dextran droplets, we recovered the expected shape without bias and found excellent agreement between Δ*n* measurements extracted from QPI images and those expected on the basis of digital refractometry measurements, now over a much larger range (Fig. 1j and Supplementary Fig. 6). Taken together, these data demonstrate that the present analysis pipeline extracts accurate geometric and optical measurements of homogeneous sessile droplets from QPI image data for Δ*n* of at least 0.085. With d*n*/d*c* estimated from protein sequences^32^ or measured by refractometry when possible (Fig. 1k, Supplementary Fig. 3 and Supplementary Note 1), Δ*n* measured by QPI, and *c*_dil_ measured by standard analytical methods or neglected (Supplementary Note 6), equation (3) enables calculation of condensed-phase protein concentrations in binary systems (Supplementary Note 7 (ref. ^32^)).

### Condensates of native-like proteins

**Fig. 2:**
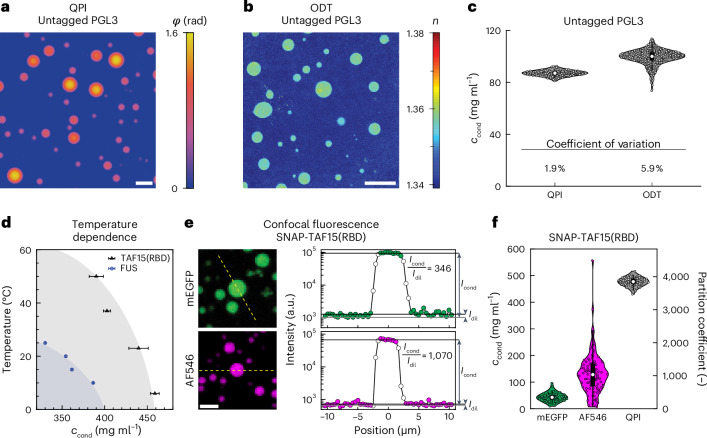
Label-free composition measurement of multidomain protein condensates. **a**,**b**, Quantitative phase (**a**) and refractive index (**b**) images of untagged full-length PGL-3 condensates acquired by QPI (*λ* = 650 nm, 75 mM KCl, 25.0 °C) and ODT (*λ* = 532 nm, 87 mM KCl, 21.5 °C). Scale bar, 10 µm. **c**, PGL-3 concentration measured from individual condensates by QPI (*N* = 269) or ODT (*N* = 355). **d**, Temperature dependence of protein concentration in condensates of mEGFP-TAF15(RBD) (black triangles) and FUS-mEGFP (blue circles) measured by QPI in buffer containing 150 mM KCl. Each datapoint represents the mean of a population of condensates from a single sample. In ascending order of temperature, error bars are s.e.m. from *N* = 1,068, 894, 571 and 208 condensates (FUS) or *N* = 41, 18, 28 and 31 condensates (TAF15). Shaded regions denote the two-phase regime for each system. Parabolic boundaries are not fits and are meant only as a guide to the eye. **e**, Confocal fluorescence images of SNAP-TAF15(RBD) condensates doped with either 23% mEGFP-TAF15(RBD) or 12% AF546-SNAP-TAF15(RBD) at 22 °C in buffer containing 100 mM KCl. Scale bar, 5 µm. Intensity profiles along the dashed yellow lines are shown on the right. Grey lines denote the average detector background. Pixels near the droplet interface (open circles) were excluded from analysis. **f**, Comparison of SNAP-TAF15(RBD) concentrations in individual condensates (left) and partition coefficients (right) measured by QPI (*N* = 119 condensates) or confocal fluorescence intensity ratios of mEGFP (*N* = 107 condensates) or AF546 (*N* = 104 condensates). For each condition, condensates are from a single sample in buffer containing 100 mM KCl and either 50 mM Tris–HCl (pH 7.4) with 5% (v/v) glycerol at 22 °C (fluorescence) or 10 mM Tris–HCl (pH 7.4) with 1% (v/v) glycerol at 37 °C (QPI). Variation from the differences in environmental conditions are small compared to the variation between fluorescence and QPI (Supplementary Fig. 8). To convert between dense-phase concentrations and partition coefficients, the dilute-phase protein concentration is taken from the literature^7^ to be 1.97 µM. For the violin plots in **c**,**f**, white circles denote medians, thick black bars are the interquartile range and whiskers extend 1.5× beyond the interquartile range. Source data

To demonstrate the suitability of our method for condensates reconstituted with native full-length proteins, we first investigated PGL-3, a major component of P granules in *Caenorhabditis* *elegans*^8^ that forms condensates in vitro^6^. Using QPI (Fig. 2a), we found the concentration in individual PGL-3 condensates is symmetrically distributed about a mean of 87.0 ± 0.1 mg ml^−1^ (s.e.m., *N* = 269) (Fig. 2c), approximately 1,000-fold higher than that in the coexisting dilute phase. The standard deviation of the measured population is only 1.7 mg ml^−1^ (Fig. 2c), yielding a low coefficient of variation (1.9%) which reflects both the high precision of the QPI method and the low droplet-to-droplet variation expected near phase equilibrium. Given the impracticality of bulk label-free measurements for this untagged full-length protein (Supplementary Note 2), we used optical diffraction tomography (ODT)^10,46^ for comparison (Fig. 2b,c). We find ODT provides accuracy comparable to that of QPI, although with reduced precision (Fig. 2c). Compared with a coefficient of variation of approximately 20% in previous concentration measurements of Tau-protein condensates protein by another tomographic phase-imaging approach^27^, both QPI and ODT provide greater precision.

We next formed condensates using constructs derived from full-length FUS protein and the RNA-binding domain of TAF15, TAF15(RBD), both reported previously in ref. ^7^), and measured their temperature-dependent composition with QPI (Fig. 2d, Supplementary Fig. 7, Supplementary Table 1 and Supplementary Note 8 (refs. ^27,29,46–49^). Interestingly, we find these condensates to be much denser than those of PGL-3 (Supplementary Table 1), with the 34% polymer volume fraction in SNAP-TAF15(RBD) condensates at 37 °C comparable to that in protein crystals^50^. Taken together, these data not only reveal that protein sequence can tune condensate composition over at least a 5-fold range, but also demonstrate that the QPI method enables precise label-free measurements on condensates of full-length proteins. This removes the primary practical barrier preventing study of native full-length proteins that are available only in limited quantities but which are most physiologically relevant.

### Influence of fluorescent labels

Conventional approaches to study condensates of full-length proteins typically require fluorescent labels, introducing two complications in phase-separating systems. First, large GFP- or SNAP-tags may alter the same phase behaviour they are being used to measure by shifting the balance of the polymer–solvent interactions that drive demixing. Second, fluorophore photophysics may vary between the starkly different chemical environments presented by the two phases (Supplementary Table 1). We leveraged label-free QPI to assess both of these potential effects. In the case of PGL-3, we found that fusion to a monomeric enhanced green fluorescent protein (mEGFP) tag increases the protein mass concentration in the condensed phase by 14% (Supplementary Table 1). As the tag increases the construct’s molecular weight by more than 14%, this actually corresponds to a decrease in the molar protein concentration, consistent with the tag imparting a modest solubilizing effect. Together with previous reports that labelling with small organic fluorophores increases the critical temperature of γ-crystallins^16^, these data suggest that fluorescent labels may promote or suppress demixing, depending on the tag.

**Extended Data Fig. 2:**
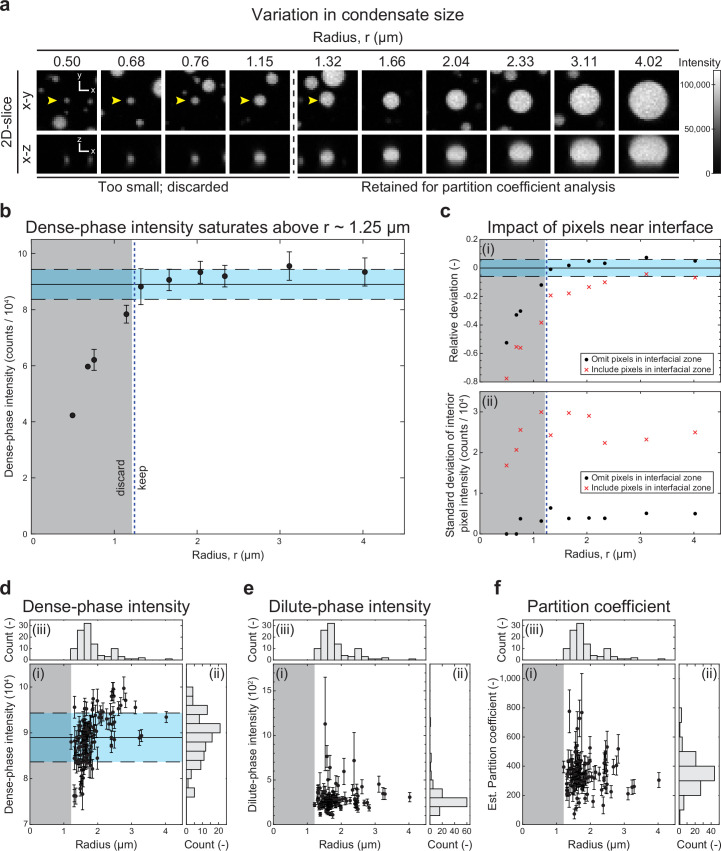
Impact of condensate size on partition coefficient estimation by fluorescence intensity. **a**, Confocal fluorescence images of SNAP-TAF15(RBD) condensates doped with mEGFP-TAF15(RBD) in buffer containing 100 mM KCl at 22 °C. Yellow arrowheads indicate the condensate of interest when larger objects are also present. White coordinate axes denote 2 µm in the indicated directions and all images are displayed with the same contrast. **b**, Dense-phase intensity of the condensates from **a** as a function of size. Datapoints and error bars denote mean and s.e.m. of interior pixel intensities along a 51-pixel linescan through the centre of each condensate, omitting pixels in the interfacial zone (see Methods). Error bars are absent for the two smallest condensates because each contain only a single interior pixel. Blue region denotes the mean ± one standard deviation ((8.90 ± 0.53) × 10^4^ counts) from the N = 107 condensates of size *r* ≥ 1.21 µm from Fig. 2e,f. For reference, the dashed vertical line (dark blue) denotes a lateral distance of 3 pixels (1.25 µm) in the images of **a**. The apparent dense-phase intensity saturates for droplets larger than *r* ~ 1.25 µm. This is comparable to twice the theoretical decay-length of the microscope’s point-spread function in the z-direction (2*l*_*z*_ ~ 1.19 µm, Methods). **c**, Impact of pixels near the interface between dense and dilute phases on dense-phase intensity quantification. **c** (**i**), Relative deviation of the apparent dense-phase intensity of individual droplets in **a** from the mean of 107 large condensates calculated with omission (black circles) or inclusion (red Xs) of pixels in the interfacial zone. Inclusion of interfacial pixels suppresses the apparent dense-phase intensity and approximately doubles the minimum size at which the dense-phase intensity estimate reaches the population mean. **c** (**ii**), Standard deviation of interior pixel intensities for the droplets in **a**, calculated with omission or inclusion of pixels in the interfacial zone. Distribution widths do not show a strong size-dependence over the range investigated. Inclusion of interfacial pixels broadens the distributions approximately 5-fold. **d**–**f** (**i**) Size-dependence of dense-phase intensity (**d**), dilute-phase intensity (**e**), and partition coefficient estimate (**f**) for the 107 large droplets of Fig. 2e,f. Distributions of each quantity are shown in side- and top-panels (**ii**, **iii**). Error bars in **d** and **e** denote s.e.m. of pixel values in the corresponding region for each individual linescan. The linescan through the largest droplet contains N = 18 interior pixels, N = 27 dilute-phase pixels and 6 pixels in the interfacial exclusion zone. Error bars in **f** denote propagation of uncertainty from the dense- and dilute-phase intensities assuming uncorrelated errors. The Pearson correlation coefficients between condensate radius and apparent dilute-phase intensity (+0.0717) and partition coefficient estimate (−0.0576) are small and statistically insignificant (p = 0.23 and 0.28, respectively). We observe a modest correlation between size and apparent dense-phase intensity (+0.5198, p < 10^−5^), though this stems primarily from the smallest condensates in the dataset. For the subset of condensates farther into the saturated portion of the intensity vs size curve (*r* ≥ 2 µm, N = 25), this correlation falls to -0.0750 and is statistically insignificant (p = 0.40). All p-values are one-sided and computed from a simulation procedure, see Methods. The mean partition coefficient estimate for the *r* ≥ 2 µm subset is 330 ± 106, slightly smaller than the 348 ± 119 estimate from all N = 107. This suggests that the systematic error incurred by inclusion of condensates with 1.21 µm ≤ *r* < 2 µm in our analysis in Fig. 2e,f is negligible compared to other sources of variation. Taken together, these data indicate that size-dependent variation in apparent dense-phase intensity is far too small in the relevant size-range to account for the 10-fold deviation we observe between partition coefficient estimates by mEGFP fluorescence vs QPI. Source data

**Extended Data Fig. 3:**
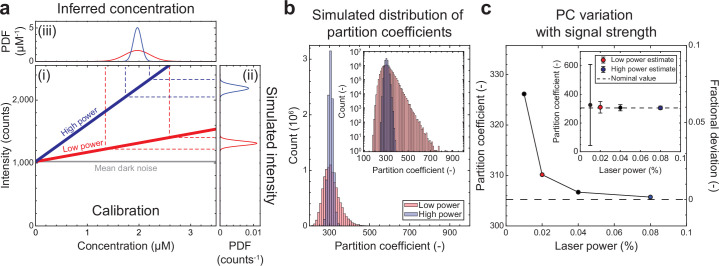
Impact of low fluorescence intensity on partition coefficient estimation. **a**, Estimation of concentration from fluorescence intensity measurements. **a.i** Typical calibration curves relating the intensity measured on a linear detector to the concentration of fluorescent molecules. The slope of the calibration curve is proportional to the power of the excitation laser. Red and blue calibration curves correspond to the low power (0.02%) used in the experiments of Fig. 2e, f and 4-fold higher power (0.08%), respectively. The mean dark noise of the detector (grey, 1023 counts) is independent of laser power and chosen to match that observed in the mEGFP-labelled measurements of Fig. 2e, f. Dashed lines are guides to the eye intended to clarify the inter-relationships between the calibration curves and the distributions in **a.ii** and **a.iii**. **a.ii** Intensity distributions expected from imaging a sample of fixed concentration (here 1.97 µM) at either low (red) or high (blue) power. Fluorescence detection is modelled as a Poisson process and simulated via the Monte Carlo method (${N}_{{draw}}={10}^{7}$). For each laser power, the mean value of the Poisson distribution is taken to be the intensity from the corresponding calibration curve at ${c}_{{dil}}^{{nom}}$ = 1.97 µM. Increasing laser power increases the magnitude of the simulated intensity above background. **a.iii** The distribution of concentrations inferred from the simulated intensity distributions (**a.ii**) using the calibration curves (**a.i**) at each power. The comparatively shallow slope of the low-power calibration curve maps the simulated low-power intensities to a comparatively wide range of concentrations relative to the high-power case. Crucially, the concentration distributions inferred for low and high power have identical mean values. This demonstrates that adjusting imaging parameters to increase signal magnitude relative to background does not influence the concentration value obtained as the average of many observations. This is because changes in the calibration curve between imaging conditions compensate perfectly for the resulting change in signal size. Instead, changing parameters like laser power alters the spread in a distribution of individual measurements, which impacts the certainty in the mean value for a given number of observations. **b**, Partition coefficient distributions calculated using a fixed dense-phase concentration estimate (${c}_{{cond}}^{{Est}}$ = 601 µM, estimated from mean of N = 107 droplets imaged at low power) and dilute-phase concentrations obtained from simulation with low (red) or high (blue) power. The distributions are both peaked near the nominal partition coefficient (${P}_{{nom}}={c}_{{cond}}^{{Est}}/{c}_{{dil}}^{{nom}}=305$) but skewed towards high values (inset). **c**, Because of the skew, the means of the simulated partition coefficient distributions (datapoints, left y-axis) slightly over-estimate the nominal value of the partition coefficient (grey). The inset shows the same data with error bars given by the standard deviation of the partition coefficient distributions (N = 10^7^ simulated values). The fractional deviation of the mean simulated values from ${P}_{{nom}}$ (right y-axis, main panel) decreases with increasing laser power. In particular, this shows that excess uncertainty in dilute-phase estimates will tend to systematically increase rather than decrease estimates of the partition coefficient. We conclude that noise from the small fluorescence signal above background is not responsible for the large difference we measure between partition coefficients by fluorescence and QPI. Source data

To test for environmentally sensitive fluorescence, we used scanning confocal microscopy to measure fluorescence in SNAP-TAF15(RBD) condensates doped with either mEGFP-tagged or Alexa Fluor 546-SNAP-tagged (AF546) constructs (Fig. 2e and Extended Data Figs. 2 and 3). The partition coefficient *P* ≡ *c*_cond_/*c*_dil_ of ∼350 obtained from mEGFP fluorescence suggests a condensed-phase concentration of only 43 mg ml^−1^, underestimating the 477 ± 14 mg ml^−1^ value we measure with QPI by over 10-fold (Fig. 2f and Supplementary Fig. 8). This indicates that the relationship between fluorescence intensity and concentration differs between phases. We suspect that enhanced quenching from the high protein concentration in the condensed phase is largely responsible for the decreased quantum yield we infer there^51^. Surprisingly, fluorescence-based assessment of partitioning using the more solvent-accessible AF546-labelled construct underestimates the concentration by around 3.6-fold (Fig. 2f). This lower magnitude of mismatch is similar to that reported for Tau-protein condensates labelled with another small organic dye, FITC^27^. The larger spread we observe in the *c*_cond_ estimate for the AF546-doped condensates relative to those with mEGFP probably results primarily from the smaller difference between the dilute-phase signal and the detector background for AF546 (Extended Data Fig. 3). However, the increased shot noise in the dilute-phase intensity estimate is unlikely to account for the shift in the distribution centre (Extended Data Fig. 3). The differential sensitivity we see with different fluorophores thus suggests that brightness may vary for each dye/condensate pair and be challenging to correct for a priori. Taken together, these data demonstrate that fluorescent labels compromise condensate composition measurements in two distinct ways, sometimes dramatically, underscoring the importance of label-free approaches such as QPI.

### Complex ageing dynamics in binary systems

**Fig. 3:**
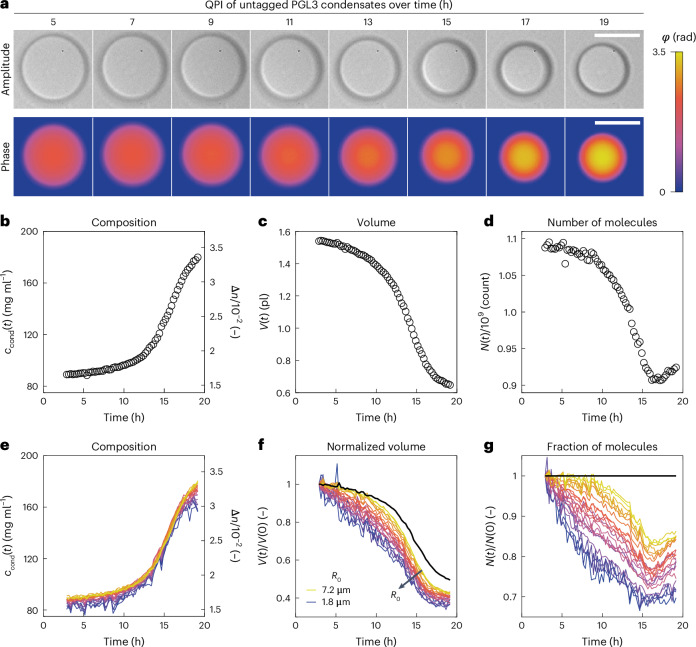
Time-resolved QPI reveals complex ageing dynamics in binary systems. **a**, Timelapse of untagged PGL-3 condensate in 75 mM KCl at 25 °C (top, QPI amplitude; bottom, QPI phase). Scale bar, 10 µm. **b**–**d**, Concentration and refractive index difference (**b**), volume (**c**) and number of protein molecules in the condensate (**d**) for the condensate shown in **a**. Time is relative to induction of phase separation. **e**, Time dependence of concentration and refractive index difference is similar for *N* = 23 differently sized condensates from the same field of view. **f**, Normalized volume varies continuously with initial condensate size *R*_0_. Initial shrinkage rate decreases with increasing *R*_0_. **g**, Fraction of molecules in 23 individual condensates over time. Thick black lines in **f**,**g** show volume and molecule count dynamics if protein mass were conserved inside condensates. Source data

Motivated by recent work demonstrating that the mechanical properties of many protein condensates undergo an ageing process^33,52–54^, we hypothesized that there may be a corresponding change in composition as condensates age. To this end, we used QPI to measure the composition of individual PGL-3 condensates over 20 h (Fig. 3 and Supplementary Video 1). During this time period, we observed droplets to shrink noticeably (Fig. 3a, top), as previously shown^33^. Although the shrinkage would be apparent by simple bright-field imaging, QPI indicated that the optical phase shift also increased with time, despite the reduction in droplet size (Fig. 3a, bottom, and Supplementary Video 1). By fitting the QPI data as before, we measured the composition (Fig. 3b) and volume (Fig. 3c) of individual condensates over time, revealing surprisingly coordinated dynamics. From these concentration and volume data, we calculated the number of proteins in the condensate (Fig. 3d) and found that the nearly 2-fold concentration increase was nearly balanced by volume decrease, such that the total number of protein molecules in the condensate decreased by only 15%. These observations indicate that the condensate necessarily expelled a significant amount of solvent while ageing.

To quantify whether this near-cancellation was serendipitous for this particular condensate, we analysed the dynamics of 22 additional condensates with a range of initial sizes over the same period (Fig. 3e–g and Supplementary Video 1). Strikingly, we found that the kinetics and extent of concentration increase were nearly identical for all condensates, independent of size (Fig. 3e and Supplementary Note 9 (refs. ^32,33,36,55–58^)). In contrast, the kinetics and extent of the volume decrease both showed systematic size dependencies, with smaller condensates losing volume faster and to greater extent than larger condensates (Fig. 3f). As a result, the fraction of molecules retained shows a marked dependence on condensate size, with larger condensates retaining more molecules (Fig. 3g and Supplementary Note 10 (refs. ^33,48,59–63^)). We speculate that the size dependence of the volume kinetics may be a signature of Ostwald ripening operating alongside the ageing process that drives the constriction and water expulsion. Taken together, these data demonstrate the suitability of QPI for monitoring the composition of many individual droplets in parallel, providing insight into the complex interplay between the physical processes driving ripening and ageing.

### Multicomponent composition from ATRI

**Fig. 4:**
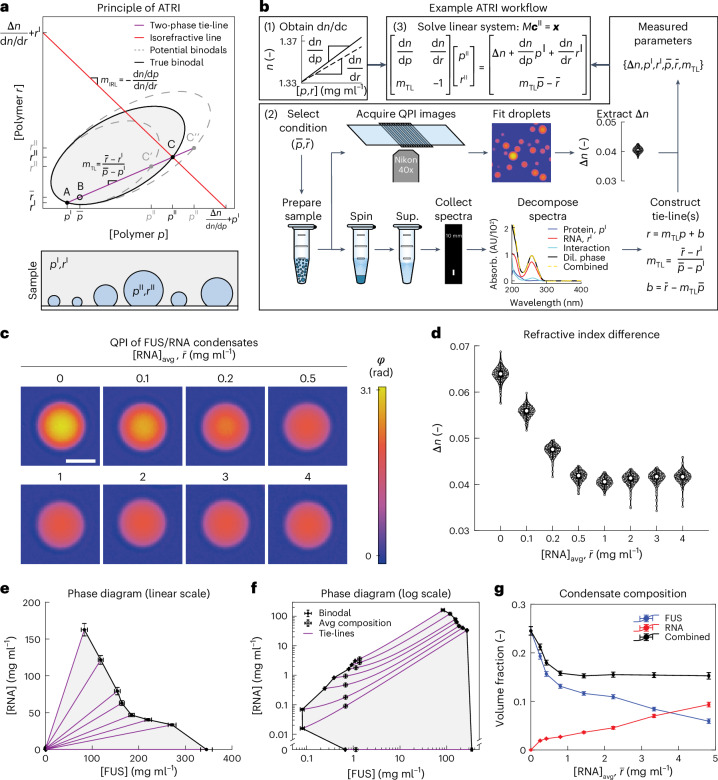
Composition determination of multicomponent FUS/RNA condensates by ATRI. **a**, Top: schematic of the multicomponent measurement approach illustrated for a model ternary system. Condensate composition (*p*^II^,*r*^II^) is determined by the intersection of the tie-line with the line of constant refractive index (isorefractive line). Bottom: schematic of coexisting multicomponent phases. **b**, Workflow to obtain all parameters in the system of linear equations. (1) Refractive index increments are determined once for each partitioning component. (2) For each tested composition $\left(\bar{p},\bar{r}\right)$ in the two-phase regime, Δ*n* is measured with QPI (example for the FUS/RNA sample prepared with $\bar{r}$ = 1 mg ml^−1^) and the dilute phase composition (*p*^I^,*r*^I^) is measured separately. For the ternary FUS/RNA mixture here, it is inferred from decomposition of UV–vis absorption spectra (example for the FUS/RNA sample prepared with $\bar{r}$ = 4 mg ml^−1^). The points $\left(\bar{p},\bar{r}\right)$ and (*p*^I^,*r*^I^) suffice to define the tie-line, whose slope *m*_TL_ is the final required parameter to solve the linear system, (3). **c**, Phase images of similarly sized FUS/RNA condensates for a range of RNA concentrations $\bar{r}$ in buffer with 150 mM KCl at 20 °C. Scale bar, 3 µm. **d**, Measured Δ*n* distributions for the samples in **c**. In order of increasing $\bar{r}$, the distributions include measurements of *N* = 153, 295, 351, 382, 257, 514, 429 or 322 individual condensates (grey). Condensates are from a single sample per condition. In the violin plots (here and in **b**), white circles denote medians, thick black bars are the interquartile range and whiskers extend 1.5× beyond the interquartile range. **e**,**f**, Experimentally determined ternary phase diagram on linear (**e**) and log–log axes (**f**). Error bars denote s.d. for dilute-phase (*N* = 3 replicates from a single sample per condition) and average concentrations (derived from *N* = 3 replicates of the stock solution). For condensed-phase concentrations, error bars denote Jacobian-based error propagation. **g**, Polymer volume fractions in the condensed phase as a function of $\bar{r}$ with $\bar{p}$ = 2 mg ml^−1^. Error bars in *y* correspond to the uncertainty in condensed-phase concentration rescaled by polymer partial specific volumes. Error bars in *x* represent standard propagation of (uncorrelated) errors from the phase diagram in **e**. Source data

Because condensates in cells are typically enriched in several distinct biomolecular species, we next asked whether the refractive index difference measured with QPI could be used to specify the composition of reconstituted condensates with multiple components. While knowledge of Δ*n* constrains Δ*c* to a single value in the binary systems studied above, the challenge for systems with multiple solutes is that Δ*n* constrains the Δ*c*_*i*_ only to an (*N* − 1)-dimensional manifold populated by compositions of equal refractive index. For a ternary system with *N* = 2 solutes and one solvent, for instance, this manifold can be visualized in the linear approximation as a line in the (*c*_1_,*c*_2_) plane (Fig. 4a). Because all compositions along this isorefractive line are compatible with the measured Δ*n*, additional relationships between the Δ*c*_*i*_ are required to uniquely specify condensate composition.

Here, we take advantage of the fact that a tie-line connects the total system composition averaged over both phases, $\left({\bar{c}}_{1},{\bar{c}}_{2},\ldots ,{\bar{c}}_{N}\right)\equiv \bar{{\boldsymbol{c}}}$, to the compositions of the coexisting phases **c**^I^ and **c**^II^ located on the (*N* − 1)-dimensional dilute and condensed binodal manifolds, respectively. For ternary systems, the binodals can be visualized as bounded curves in the (*c*_1_,*c*_2_) plane (Fig. 4a). Mass conservation guarantees that tie-lines are straight (Fig. 4a and Supplementary Note 11 (ref. ^64^)), providing *N* − 1 linearly independent constraints. This can be seen by noting that projections of the tie-line in each of the *N* − 1 (*c*_1_,*c*_i≠1_) planes must all be straight. In principle, tie-line constraints of the form ${c}_{i}={m}_{1i}{c}_{1}+{b}_{1i}$ where ${m}_{1i}=\left({\bar{c}}_{i}-{c}_{i}^{\mathrm{I}}\right)/\left({\bar{c}}_{1}-{c}_{1}^{\mathrm{I}}\right)$ and ${b}_{1i}={\bar{c}}_{i}-{m}_{1i}{\bar{c}}_{1}$ for *i* ≠ 1 can be obtained from knowledge of overall sample conditions, $\bar{{\boldsymbol{c}}}$, and the composition measurements of the abundant dilute-phase ${c}_{i}^{\mathrm{I}}$ using traditional approaches from analytic chemistry (Supplementary Notes 6 and 12). In the linear approximation of equation (2), these form a linear system of *N* equations of the form $M{{\boldsymbol{c}}}^{\mathrm{II}}={\boldsymbol{x}}$, where the matrix $M$ and vector ${\boldsymbol{x}}$ contain measured quantities (see Fig. 4b and Supplementary Note 12 for explicit expressions for ternary and (*N* + 1)-component systems, respectively). By solving this system of equations, the composition of the multicomponent condensate is written in terms of known optical quantities and concentrations as

4 $${c}_{i}^{\mathrm{II}}=\Delta n{M}_{i1}^{-1}+{c}_{i}^{\mathrm{I}},$$

where ${M}_{i1}^{-1}$ are elements of the system’s matrix inverse (Supplementary Note 12). We refer to this method of composition determination as ATRI and illustrate an extension to the non-linear case in Supplementary Note 13. By leveraging tie-line information, the ATRI method is, in principle, capable of resolving the composition of biomolecular condensates with a potentially large number of components.

### Phase diagram for a ternary system with RNA and full-length FUS

**Extended Data Fig. 4:**
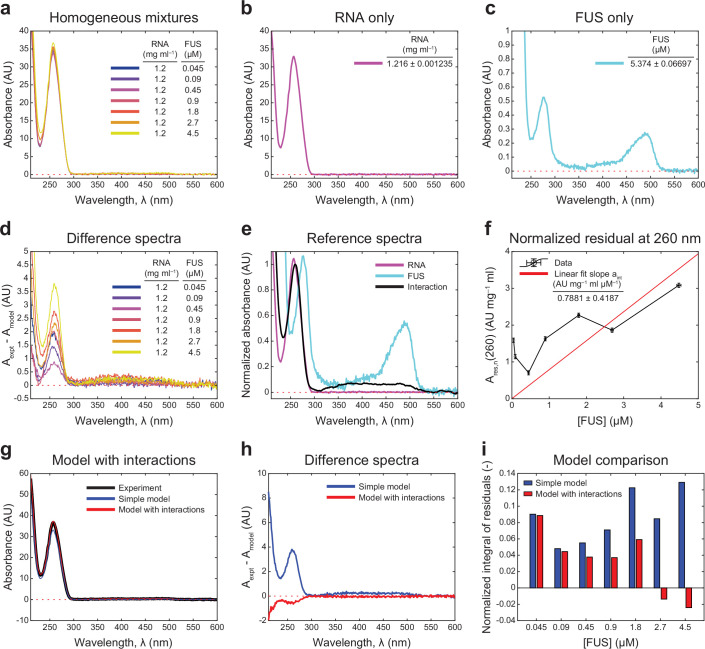
Inclusion of interaction term in UV-Vis absorption model improves agreement with experiment for homogeneous FUS-RNA mixtures. **a**, Absorption spectra of homogeneous mixtures containing 1.2 mg ml^−1^ poly(A) RNA and a range of FUS-mEGFP (concentrations indicated in legend) acquired with a 0.07-mm pathlength. These samples were prepared in the 1-phase regime to ensure homogeneity. All spectra presented in AU were rescaled to a pathlength of 10 mm. **b**, **c**, Reference absorption spectra of homogeneous solutions containing either 1.2 mg ml^−^^1^ poly(A) RNA acquired with a 0.07-mm pathlength (**b**) or 5.4 µM FUS-mEGFP acquired with a 10-mm pathlength (**c**). Different pathlengths were used to compensate for the large differences in extinction of the two samples. These single-species reference spectra were used to model the spectra of two-species mixtures. **d**, Difference spectra obtained by subtraction of a minimal mixture model from the spectra in **a**. For each trace, the minimal mixture model is the sum of the reference spectra in **b** and **c**, weighted by the concentrations of RNA and protein added to the solution. The difference spectra show a strong peak near 260 nm that varies with FUS concentration, as well as a low broad peak around 400 nm. Strong concentration-dependent spectral features indicate that the mixture spectra are not well-described by the minimal model of a sum with two independent terms. **e**, Comparison of normalized difference spectra (yellow trace from **d**) to normalized RNA and protein reference spectra from **b** and **c**, respectively. Owing to the similar 260-nm peaks in the difference and RNA reference spectra, attempts to infer unknown RNA and protein concentrations in a homogeneous mixture by linear decomposition with a two-term model will tend to overestimate the RNA concentration. **f**, Residual absorption normalized per unit RNA concentration, ${A}_{{res},n}\equiv \left({A}_{{mix}}-{A}_{{model}}\right)/{c}_{{RNA}}$, measured at 260 nm as a function of the FUS concentration added to the mixture. Error bars in ${A}_{{res},n}$ represent Jacobian-based propagation of uncorrelated uncertainties in the mixture spectra as well as in the two reference spectra in the model. Uncertainties for each spectrum are the standard deviation of at least N = 5 measurements. Error bars in the FUS concentration are proportional to the standard deviation of the FUS-only reference spectra at 280 nm. ${A}_{{res},n}$ at 260 nm is strongly correlated with FUS concentration (Pearson’s correlation coefficient r = 0.8652, p = 0.0119 is computed as a one-sided value from a simulation procedure, see Methods), though the exact concentration-dependence appears complex. We use a linear fit (red) constrained to pass through the origin as a simple approximation for this behaviour. The fitted slope, ${a}_{{Int}}=0.79\pm 0.42$ in units of ${AU}\times {\left(\frac{{mg}}{{ml}}{RNA}\right)}^{-1}\times {\left({\mu }{M\; FUS}\right)}^{-1}$, corresponds to the coefficient of the first cross-term in a series expansion and represents the lowest-order contribution of FUS-RNA interactions to the absorption spectra of the mixture. **g**, Absorption spectra of FUS-RNA mixture with 4.5 µM FUS (black), a minimal two-term absorbance model (blue), and an absorbance model including an interaction term with coefficient determined in **f** (red). **h**, Difference spectra calculated between the mixture with 4.5 µM FUS and each of the two models. Inclusion of the interaction term significantly reduces the residuals across the spectrum. **i**, Comparison of the integral of model residuals from 230 to 600 nm for each mixture in **a**. Residuals for the minimal two-term model (no interaction term) are in blue, and those for the three-term model (with interaction term) are in red. Integrated residuals are normalized by the integral of the spectrum of each mixture, and can be nearly thought of as the fraction of the integrated absorbance unaccounted for by the model. In all cases, inclusion of the interaction term improves agreement between the measured spectrum and the model. Source data

**Extended Data Fig. 5:**
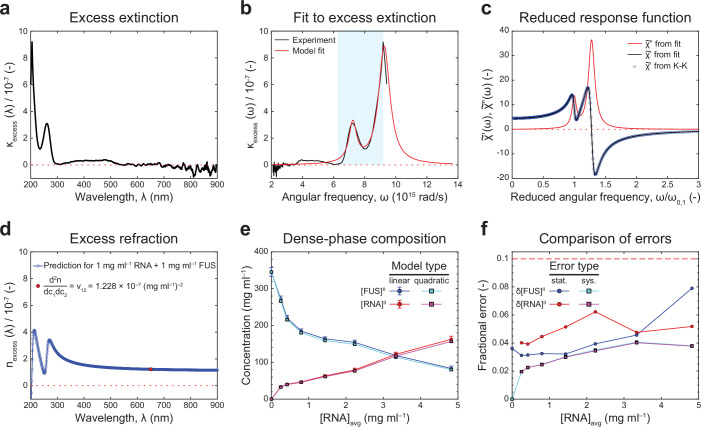
Estimating the FUS/RNA refractive index cross-term coefficient from excess absorption. **a**, Excess extinction of a homogeneous FUS/RNA mixture over the full range of wavelengths accessible by standard UV-VIS spectroscopy (200 nm to 900 nm). The excess extinction is calculated from the excess absorbance spectra as ${\kappa }_{{excess}}\left(\lambda \right)=\lambda {A}_{{excess}}\left(\lambda \right)/\left(4\pi L{\log }_{10}\left(e\right)\right)$ using ${A}_{{excess}}\left(\lambda \right)$ measured spectroscopically for 1.2 mg ml^−^^1^ RNA with 4.5 µM FUS (Extended Data Fig. 4), rescaled for 1 mg ml^−^^1^ RNA and 1 mg ml^−^^1^ FUS and a pathlength of L = 10 mm. The spectrum is dominated by two resonances, one near 260 nm a second near 200 nm. **b**, Excess extinction from **a** as a function of angular frequency (black) and fit to the dissipative component of a model for two driven linearly-damped linear oscillators (red, Adj. R^2^ = 0.9781) over the domain indicated in light blue (corresponding to $\lambda \in \left[\mathrm{205,300}\right]$ nm). The model equation is ${\chi }_{2-\text{osc}.}^{{\prime} {\prime} }\left(\omega \right)={Z}_{1}\frac{{\Gamma }_{1}\omega }{{\left({\omega }_{\mathrm{0,1}}^{2}-{\omega }^{2}\right)}^{2}+{\left({\Gamma }_{1}\omega \right)}^{2}}+{Z}_{2}\frac{{\Gamma }_{2}\omega }{{\left({\omega }_{\mathrm{0,2}}^{2}-{\omega }^{2}\right)}^{2}+{\left({\Gamma }_{2}\omega \right)}^{2}}$ and details are given in Supplementary Note 14. Though imperfect, this fit captures the essential spectral features well, including the strength and location of the two primary resonances. **c**, Reduced optical response function versus angular frequency normalized by the lower natural frequency of the two-oscillator model, ${\omega }_{\mathrm{0,1}}$. The imaginary component (red) comes from rescaling the fit in **b** according to ${\widetilde{\chi }}^{{\prime} {\prime} }={\kappa }_{{excess}}/{\widetilde{Z}}_{1}$, where ${\widetilde{Z}}_{1}={Z}_{1}/{\omega }_{\mathrm{0,1}}^{2}$ is the reduced amplitude of the first oscillator in the fit. The real component ${\widetilde{\chi }}^{{\prime} }$ is computed in two ways, by integrating ${\widetilde{\chi }}^{{\prime} {\prime} }$ in the Kramers-Kronig relation ${\chi }^{{\prime} }\left(\omega \right)=\frac{2}{\pi }{\mathcal{P}}{\int }_{0}^{+\infty }\frac{{\omega }^{{\prime} }{\chi }^{{\prime} {\prime} }\left({\omega }^{{\prime} }\right)}{{\omega }^{{\prime} 2}-{\omega }^{2}}d{\omega }^{{\prime} }$ (blue circles) and also as the corresponding elastic component of the two-oscillator model ${\chi }_{2-\text{osc}.}^{{\prime} }\left(\omega \right)={Z}_{1}\frac{\left({\omega }_{\mathrm{0,1}}^{2}-{\omega }^{2}\right)}{{\left({\omega }_{\mathrm{0,1}}^{2}-{\omega }^{2}\right)}^{2}+{\left({\Gamma }_{1}\omega \right)}^{2}}+{Z}_{2}\frac{\left({\omega }_{\mathrm{0,2}}^{2}-{\omega }^{2}\right)}{{\left({\omega }_{\mathrm{0,2}}^{2}-{\omega }^{2}\right)}^{2}+{\left({\Gamma }_{2}\omega \right)}^{2}}$ (black curve). Both methods agree quantitatively, as expected. **d**, Excess refraction as a function of wavelength, obtained from the real component of the reduced response function in **c**. The value of the refractive index cross-term coefficient relevant to QPI experiments at 650 nm is 1.228 × 10^−7^ (mg ml^−^^1^)^−2^ (red circle). **e**, Condensed-phase concentrations of FUS (blue, cyan) and RNA (red, magenta) as a function of system-average RNA concentration when the refractive index differences obtained by QPI are resolved using a linear model (blue, red), as in the main text, or a minimal quadratic model including the cross-term with coefficient obtained in **d** (cyan, magenta). Neglecting the cross-term results in a small systematic over-estimation of the condensed-phase concentrations. **f**, Comparison of the magnitude of the systematic error incurred by neglecting the refractive index cross-term (FUS in cyan, RNA in magenta) to the statistical uncertainty calculated by error propagation in the context of the linear model (FUS in blue, RNA in red). Fractional statistical error is computed as the ratio of statistical uncertainty to central estimate from the linear model using values from the phase diagram in Fig. 4e. Fractional systematic error is computed as the difference between the central estimates of dense-phase concentration from the linear and quadratic models normalized by the quadratic model estimate: $\delta {c}_{{sys}}^{{II}}=\frac{{c}_{{lin}}^{{II}}-{c}_{{quad}}^{{II}}}{{c}_{{quad}}^{{II}}}$. In all cases, the systematic error is smaller than the statical uncertainty. Individually, each source is typically much less than 10% (red dashed line). Source data

**Extended Data Fig. 6:**
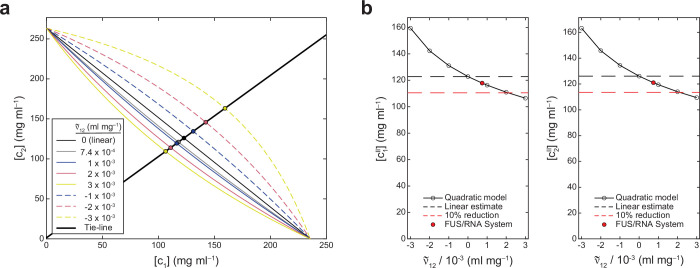
Composition estimation with a quadratic model of refractive index. **a**, Variation of the isorefractive curve in response to changes in the magnitude and sign of the reduced refractive cross-term coefficient ${\widetilde{\upsilon }}_{12}$. For each ${\widetilde{\upsilon }}_{12}$, the composition of the condensed phase is given by the intersection of the isorefractive curve with the tie-line (circles). The parameters for the linear model and tie-line correspond to those measured for the FUS/RNA mixture with the second-highest average RNA concentration in Fig. 4: ${m}_{{TL}}$ = 1.016, ${\bar{c}}_{1}$ = 1.366 mg ml^−^^1^ FUS, ${\bar{c}}_{2}$ = 2.693 mg ml^−^^1^ poly(A) RNA, ${c}_{1}^{I}$ = 0.9229 mg ml^−^^1^, ${c}_{2}^{I}$ = 2.243 mg ml^−^^1^, $\Delta n$ = 0.0415, ${dn}/d{c}_{1}$ = 1.85 × 10^−4^ (ml mg^−^^1^), and ${dn}/d{c}_{2}$ = 1.655 × 10^−4^ (ml mg^−^^1^). This sample was chosen to maximize the impact of a non-zero ${\widetilde{\upsilon }}_{12}$, as the deviation between linear and quadratic models is largest when the tie-line slope is close to 1. The isorefractive curve obtained for this system using the ${\widetilde{\upsilon }}_{12}$ calculated on the basis of excess UV absorption is shown in grey. **b**, Variation in condensed-phase concentrations ${c}_{1}^{{II}}$ (FUS, left) and ${c}_{1}^{{II}}$ (RNA, right) obtained from the intersection of the tie-line with the isorefractive curves in **a**. The condensed-phase value estimated with a linear model is indicated with a dashed black line, while the red dashed line denotes a 10% deviation from the linear estimate. A linear model underestimates the condensed-phase concentration obtained from a quadratic model when ${\widetilde{\upsilon }}_{12} < 0$ and overestimates it when ${\widetilde{\upsilon }}_{12} > 0$. For a given ${\widetilde{\upsilon }}_{12}$ magnitude, the severity of the systematic error is weaker for positive ${\widetilde{\upsilon }}_{12}$ than for negative ${\widetilde{\upsilon }}_{12}$. With our ${\widetilde{\upsilon }}_{12}$ estimate for FUS/RNA, the deviation between linear and quadratic models is substantially below 10%. Source data

To validate the ATRI approach, we used poly(A) RNA and full-length FUS-mEGFP, which localizes to RNA-rich stress granules in eukaryotes^10^, as solutes in a model ternary system (*N* = 2). Following the workflow in Fig. 4b, we prepared systems in the two-phase region for a range of total RNA concentrations. To obtain the dilute-phase composition for this system, we decomposed ultraviolet–visible (UV–vis) absorbance spectra of the dilute phase into protein and RNA contributions (Fig. 4b, Extended Data Fig. 4, Supplementary Fig. 9 and Methods). Combined with the system average compositions, the decomposed spectral data produce a set of physically compatible tie-lines (Supplementary Fig. 10). To obtain Δ*n*, we analysed QPI data (Fig. 4c), and found that Δ*n* decreases and saturates with increasing total RNA (Fig. 4d). Using these data as inputs in equation (4) (Fig. 4b), we calculated the corresponding points on the condensed binodal branch and plotted these together with the dilute binodal and tie-lines as a full phase diagram (Fig. 4e, f). This phase diagram captures the re-entrant behaviour with increasing RNA inferred previously in related systems without actually measuring the phase boundary^65,66^. Using a non-linear extension of ATRI to account for the contributions of FUS–RNA interactions to the sample’s optical response (Extended Data Fig. 4 and Supplementary Note 14 (refs. ^32,36,37,67–69^)), we find that using the linear approximation (as done in Fig. 4) overestimates dense-phase concentrations by 4% or less (Extended Data Figs. 5 and 6). In addition to connecting variation in system average composition directly to its consequences on the condensate, which was seldom accessible previously^21^, this phase diagram exposes surprising features in the condensed binodal branch, including a kink and a linear section (Fig. 4e). The ability of the ATRI method to resolve the molecular composition of multicomponent condensates reveals that increasing RNA concentrations are compensated by decreasing protein concentrations such that a constant polymer volume fraction is maintained near cytoplasmic levels (Fig. 4g), which would have been challenging to infer using conventional techniques.

### Composition of complex multicomponent condensates

**Extended Data Fig. 7:**
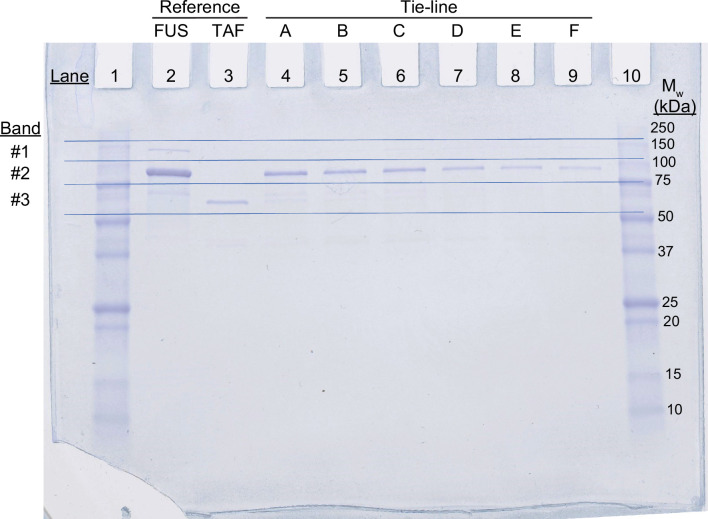
Electrophoresis gel of reference and dilute-phase samples prior to MS. Image of the Coomassie-stained SDS-PAGE gel following electrophoresis of samples in preparation for quantitative mass spectrometry analysis. Lanes 2 and 3 contain reference samples from FUS and TAF stock solutions, respectively, and were used to determine the composition of the stock solutions. Lanes 4-9 contain dilute-phase from samples of the 5-solute-system prepared on tie-lines A-F. Lanes 1 and 10 contain a molecular weight protein standard (BioRad, Cat. #1610363); corresponding molecular weights (in kDa) are given on the right-hand side. Three bands were excised from each of lanes 2-9 and independently digested and analysed by mass spectrometry. We refer to mEGFP-tagged protein in bands 1, 2 and 3 as MBP-FUS-mEGFP, FL-FUS-mEGFP, and shortFUS-mEGFP, respectively. Similarly, we refer to SNAP-tagged protein in band 3 as SNAP-TAF15(RBD). Note that the uncropped and unprocessed image for this gel supplied as source data was flipped along the horizontal axis for the presentation of this data in this figure. Source data

**Extended Data Fig. 8:**
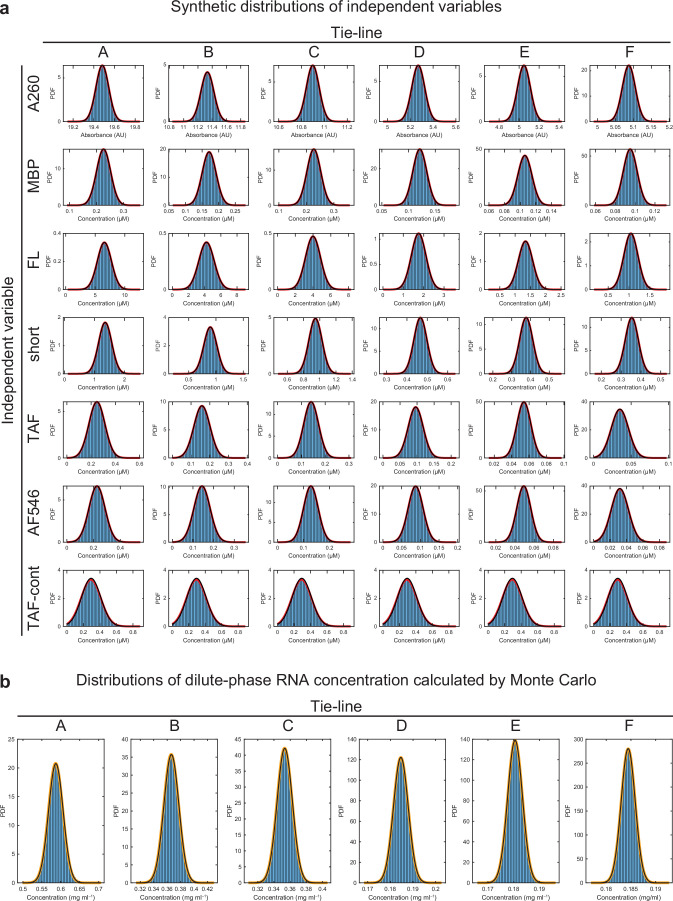
Estimation of $c_{RNA}^{I}$ via Monte Carlo simulation. **a**, Synthetic distributions of the 7 non-zero quantities allowed to vary in the calculations for each of the tie-lines A-F (columns). These quantities are the absorbance of the dilute-phase mixture at 260 nm (A260, top row) and the dilute-phase concentrations of MBP-FUS (MBP, 2^nd^ row), FL-FUS (FL, 3^rd^ row), shortFUS (short, 4^th^ row), SNAP-TAF15(RBD) (TAF, 5^th^ row), Alexa Fluor 546 (AF546, 6^th^ row), and contaminant from the TAF protein stock (TAF-cont, 7^th^ row). For all but AF546, synthetic distributions (blue) were generated via the Monte Carlo method by independently drawing ${N}_{{draw}}={10}^{7}$ values from Gaussian input distributions (red) subject to a positivity constraint. Black curves represent Gaussian fits to the synthetic data. For 6 of the 7 independent variables, parameters for the input distributions are the mean and standard deviations from dilute-phase absorbance measurements (A260), dilute-phase mass spectrometry measurements (MBP, FL, short, TAF), and estimates (TAF-cont; see Fig. S13) in which the contaminant partition coefficient is 1. AF546 is not allowed to vary independently. Instead, the synthetic data are taken as the product of the synthetic TAF data and the molar labelled fraction (${f}_{{AF}}=0.9096$). This is appropriate in the limit where the presence of the AF546 dye on the protein doesn’t influence partitioning appreciably. Input distributions for AF546 are analogously rescaled versions of the TAF input distributions. We find that the synthetic distributions are generally well-described as Gaussian, though the asymmetry introduced by the positivity constraint shifts the means of some low-abundance species (most noticeably TAF-cont) towards slightly higher values. **b**, Distributions of dilute-phase RNA concentration for each tie-line calculated using ${c}_{{RNA}}^{I}=\frac{{A}_{260}-\mathop{\sum}\nolimits_{i=1}^{6}{\varepsilon }_{i}{c}_{i}^{I}}{{\varepsilon }_{{RNA}}+{a}_{\mathrm{int}}\left({c}_{{MBP}}^{I}+{c}_{{FL}}^{I}+{c}_{{short}}^{I}+{c}_{{TAF}}^{I}\right)}$ and the synthetic distributions above. Black line is a Gaussian fit to the resulting distributions (blue), and generally describes the distributions very well. Orange curves show Gaussian distributions with means given by the equation for ${c}_{{RNA}}^{I}$ using central estimates for all relevant variables and standard deviations given by Jacobian-based error propagation (assuming AF546 and TAF are perfectly correlated and all other variables are uncorrelated). Distributions obtained by Monte Carlo (blue, black) are in excellent agreement with those derived from central estimates and error-propagation (orange), validating the Monte-Carlo-based analysis. Source data

To explicitly demonstrate that ATRI is not limited to systems with at most two chemically dissimilar solutes, we determined the composition of condensates containing poly(A) RNA along with the RNA-binding proteins FUS and TAF15(RBD). To measure the absolute abundance of each protein species in the dilute phase, we used quantitative mass spectrometry (MS)^70^. We then inferred the dilute-phase RNA concentration from decomposition of dilute-phase UV–vis spectra using the protein concentrations measured by MS (Extended Data Figs. 7 and 8, Supplementary Figs. 11–13, Supplementary Table 2 and Supplementary Note 15 (refs. ^70,71^)). Finally, we measured Δ*n* by QPI and used refractive index increments measured (RNA) or estimated from sequence (proteins) as before.

**Fig. 5:**
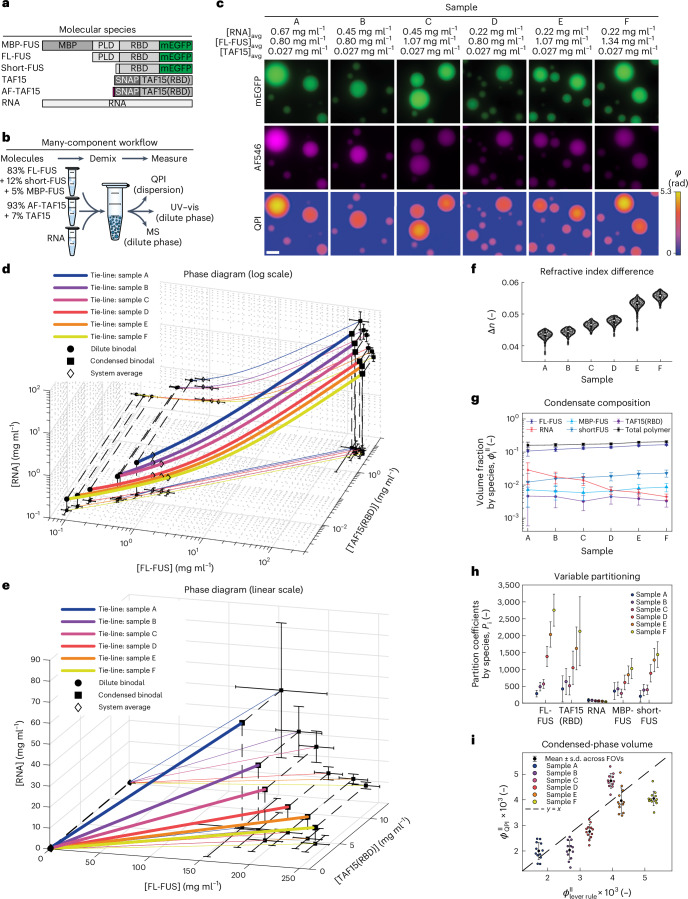
Composition determination for condensates with many components. **a**, Domain organization of the five major protein species (and poly(A) RNA) used to form many-component condensates in vitro. MBP, maltose-binding protein; FUS, fused in sarcoma; PLD, prion-like domain; RBD, RNA-binding domain; mEGFP, monomeric enhanced green fluorescent protein; FL, full-length; TAF15, TATA-box-binding-protein associated factor 15; AF, Alexa Fluor 546. **b**, Schematic of experimental workflow. Stock solutions of fixed stoichiometry (given as mass percentage) are combined to generate two-phase dispersions. Condensate composition is determined by combining MS and UV–vis absorption measurements of the dilute-phase with QPI measurements of the refractive index difference between phases in the dispersion. **c**, Samples A–F were prepared at the system-average concentrations indicated and imaged by widefield fluorescence and quantitative phase microscopy in buffer with 150 mM KCl at 20 °C. Scale bar, 6 µm. Here and in the following, TAF15 represents the sum of AF-labelled and unlabelled constructs. **d**, 3D projection of a six-dimensional phase diagram into the subspace spanned by FL-FUS, TAF15 and RNA on logarithmic scales. Dashed lines connect binodal and average composition points to their 2D projections. Error bars for dilute-phase protein and all system average concentrations represent the s.d. of multiple measurements (*N* = 2 repeats from the same sample for protein measurements by MS and *N* = 3 repeats of the RNA stock to determine the system average). Error bars for dilute-phase RNA and all condensed-phase concentrations were calculated by error propagation (Supplementary Note 12.4). **e**, Same data as in **d** plotted on linear scales. **f**, Distribution of refractive index differences measured for each sample, with *N* = 686, 601, 766, 782, 1,174 and 1,431 individual condensates (grey) from samples A–F, respectively. Condensates are from a single sample per condition. White circles denote medians, thick black bars are the interquartile range and whiskers extend 1.5× beyond the interquartile range. **g**, Fraction of condensate volume occupied by each species. Total polymer represents the sum of all five species. The central value and error bars are given by the dense-phase concentration and uncertainties in **d** (and Extended Data Fig. 10) rescaled by polymer partial specific volumes. **h**, Protein partition coefficients are computed directly from the binodal concentrations in **d** and vary strongly between tie-lines in this portion of the two-phase region. Error bars are estimated by simple error propagation assuming uncorrelated errors in the concentrations in each phase. **i**, Comparison of the total condensed-phase volume (as a fraction of the total system volume) calculated from the phase diagram in **d** via the lever rule (*x* axis) and from the sum of condensate volumes obtained in the QPI analysis (*y* axis). Individual coloured points represent different fields of view, whereas black circles and error bars represent the mean ± s.d. across the fields of view. Small within-sample scatter in *x* is added to aid visualization and does not represent uncertainty in *x*. The Pearson correlation coefficient between the phase volume fractions obtained by the lever rule and QPI is *r* = 0.827 (one-sided *P*-value is 0.022 ± 0.020 computed as mean and s.d. from 50 replicates of a simulation procedure; Methods). The good agreement between the phase volume predicted from the phase diagram and that measured by microscopy validates the compositional analysis. Source data

Gel electrophoresis performed prior to MS analysis indicated the presence of three populations with distinct molecular weights in our FUS stock solution, which we refer to as MBP-FUS, FL-FUS (full-length) and shortFUS (Extended Data Fig. 7 and Supplementary Fig. 11). We applied a modified MS western protocol^70^ to determine the absolute (molar) abundance of each population in the stock (83% FL, 12% short, 5% MBP by mass, Fig. 5a) and subsequently in the dilute phase of demixed samples.

**Extended Data Fig. 9:**
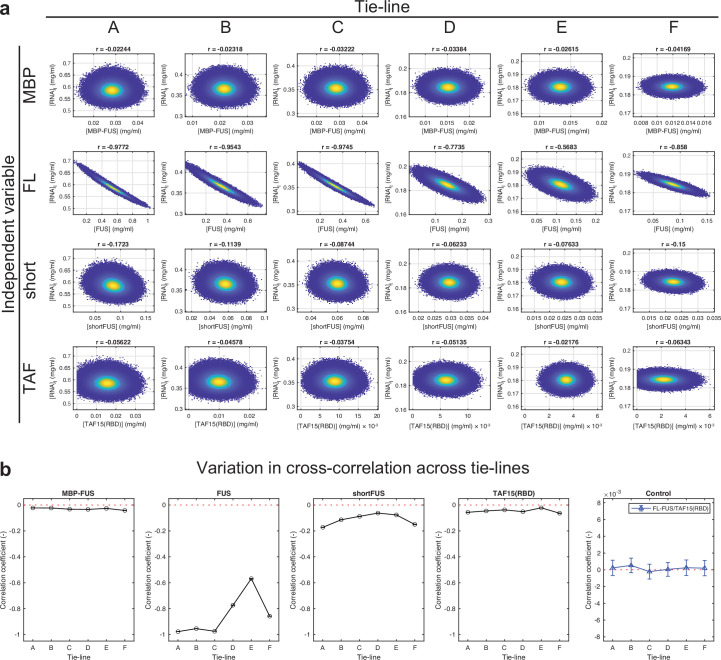
Cross-correlation estimates between $c_{RNA}^{I}$ and other dilute-phase concentrations. **a**, 2D histograms of dilute-phase RNA concentration (${c}_{{RNA}}^{I}$) obtained from Monte Carlo simulations as a function of the synthetic dilute-phase concentrations of MBP-FUS (MBP, top row), full-length FUS (FL, second row), shortFUS (short, third row), and SNAP-TAF15(RBD) (TAF, fourth row) for each of the tie-lines A-F (columns). Colour encodes relative likelihood, with white unobserved in simulations, blue observed but infrequent, and yellow observed most often. Pearson’s correlation coefficient between ${c}_{{RNA}}^{I}$ and the specified independent variable is shown above each histogram. These coefficients are used to account for cross-correlations in the error-propagation calculations for the dense-phase concentrations. **b**, Variation in Pearson’s correlation coefficient across tie-lines. Datapoints and error bars represent the mean and standard deviation, respectively, of cross-correlations calculated from 10 independent subsets of the synthetic data, each of size ${N}_{{sub}}={N}_{{draw}}/10={10}^{6}$ samples. In most cases, error bars are smaller than the size of the datapoints. Dashed red line represents a cross-correlation of zero. ${c}_{{RNA}}^{I}$ is strongly anticorrelated with the dilute-phase concentration of FL-FUS. For MBP-FUS, shortFUS, and TAF15(RBD), the cross-correlations are negative and weak, but always significantly different from zero. As a negative control, we also calculated the cross-correlation between the dilute-phase concentration of FL-FUS and TAF15(RBD) (blue, far right plot). As these molecular species were generated independently in the Monte Carlo simulation, their cross-correlation is expected to be zero. Consistent with this, we find FL-FUS/TAF15(RBD) cross-correlations to be zero, within error. This consistency check verifies our use of synthetic data from Monte Carlo simulations to estimate cross-correlations between ${c}_{{RNA}}^{I}$ and other dilute-phase concentrations for use in subsequent error propagation for dense-phase calculations. Source data

**Extended Data Fig. 10:**
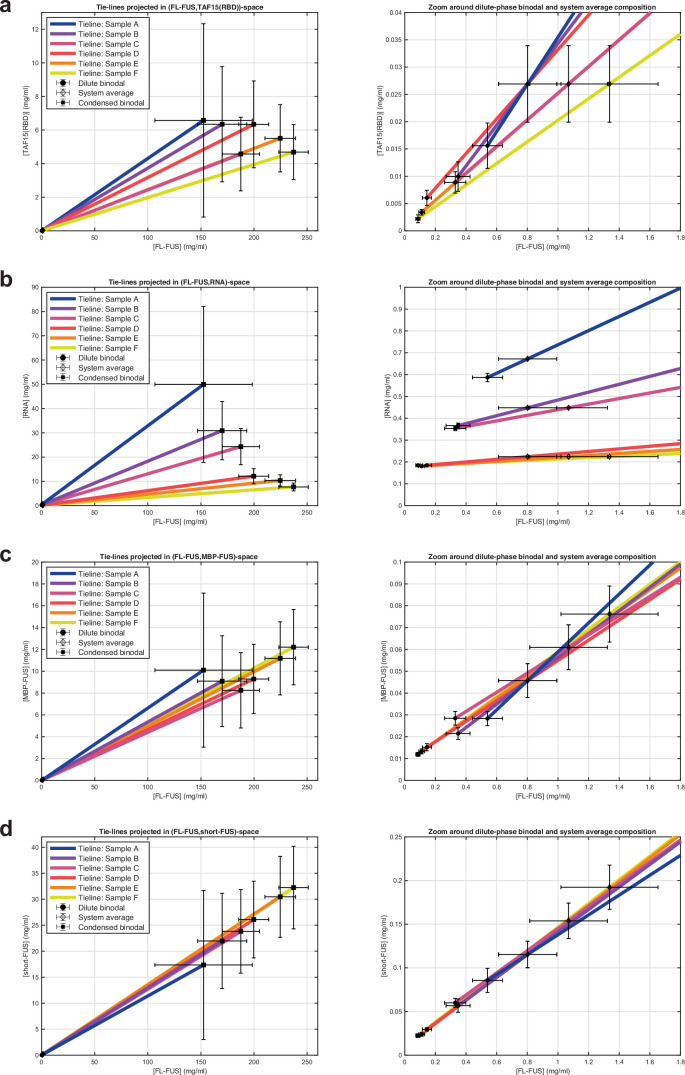
2D-projections of phase diagram for 5-solute system. Same data as in Fig. 5e, but now shown as two-dimensional projections in the planes spanned by FL-FUS and either SNAP-TAF15(RBD) (**a**), RNA (**b**), MBP-FUS (**c**), or shortFUS (**d**). Left column shows full diagram while right column zooms-in near the origin to resolve the compositions of the dilute phase (squares) and the average system compositions (diamonds). As in Fig. 5e, error bars for dilute-phase protein and all system average concentrations represent standard deviation of multiple measurements (N = 2 repeats from the same sample for protein measurements by MS and N = 3 repeats of the RNA stock to determine the system average). Error bars for dilute-phase RNA and all condensed-phase concentrations were calculated by error propagation (see Supplementary Note 12.4). Source data

In this way, we resolved the dense-phase concentrations of three closely related protein constructs (MBP-FUS, FL-FUS and shortFUS), a genetically distinct protein (TAF15(RBD)) and an RNA species for a total of five solutes. By varying the average concentration of RNA and the FUS constructs, we prepared systems on six different tie-lines in the region of two-phase coexistence and repeated the analysis (Fig. 5b–f and Extended Data Figs. 9 and 10). Despite exploring only a tiny fraction of the high-dimensional phase space, our measurements reveal significant compositional variation between tie-lines (Fig. 5g,h).

Three pieces of evidence support the validity of this high-dimensional phase diagram. First, none of the independently determined tie-lines cross (Fig. 5d; note that tie-lines which do not cross in a high-dimensional space may appear to cross when projected into a lower-dimensional space, as in Extended Data Fig. 10). Second, experimentally determined compositions of the coexisting phases vary smoothly between nearby tie-lines, consistent with the physical expectation that the binodal is a smooth manifold in regions with a constant number of coexisting phases. These two criteria establish the physical plausibility of our analysis at a qualitative level.

To validate our results quantitatively, we used our experimental phase diagram to predict the volume fraction of condensed phase present in a typical microscopy image and tested this against the total condensate volume fraction measured by QPI (Fig. 5i). We find a strong positive correlation (Pearson’s correlation coefficient *r* = 0.827, *P* = 0.022 ± 0.020) and reasonable quantitative agreement between the dense-phase volume fraction measured by QPI and that predicted from our experimental phase diagram via the lever rule. The only ‘free parameter’ in this comparison is the sample thickness inside the microscopy flowcell, which we set equal to the parafilm thickness of 130 µm quoted by the manufacturer. Taken together, these data indicate that the high-dimensional binodals and tie-lines constructed with ATRI for the five-solute system in Fig. 5 are accurate at a quantitative level.

## Discussion

Our results show that ATRI enables quantitative and label-free composition measurements of biomolecular condensates reconstituted with full-length proteins in binary and multicomponent mixtures. Here, we measured refractive index differences primarily with QPI. Our finding that a common fluorescent tag can significantly alter condensate composition (Supplementary Table 1) highlights the need for label-free measurement approaches. Microscopy methods like QPI and related tomographic techniques^27,46^ offer several advantages over label-free strategies based on bulk measurements. The first is the ~1,000-fold reduction in sample requirements, which removes the primary barrier to measurements on condensates reconstituted from native full-length proteins (Fig. 2a–c). Second, whereas time-resolved measurements of composition in response to changing solution conditions, temperature or intrinsic sample dynamics are challenging with bulk approaches, QPI’s compatibility with open samples and dry objectives makes it easy to record sample dynamics with second-scale time resolution (Fig. 3) and in response to environmental changes (Fig. 2d). Third, measuring composition of micrometre-sized condensates in situ provides access to information such as size-dependent composition (Fig. 1i), homogeneity (Fig. 1h) and dynamics (Fig. 3e–g) that are not available with bulk techniques. Finally, we note that the high precision demonstrated here with QPI not only enables the method to resolve subtle compositional differences (Supplementary Table 1) and unexpected binodal features (Fig. 4g) with high confidence, but could also serve to constrain competing thermodynamic models of condensate formation^7,22,30,31,72,73^.

The central innovation of the ATRI method is its ability to disentangle the composition of multicomponent biomolecular condensates. All endogenous condensates are expected to contain multiple components, with some of the best-characterized known to house dozens^8–10^. Knowledge of component identity and quantity will be essential for design and interpretation of increasingly faithful reconstitution experiments and for studies of chemical reactions in synthetic condensates that account for reacting species^74^. Here, we validated ATRI by measuring the full phase diagram for a ternary mixture of full-length FUS protein and RNA, including the dilute- and condensed-binodal branches and tie-lines (Figs. 4e,f and 5d,e). We note that three pieces of information, tie-lines along with both binodals, are strictly required to physically relate the compositions of coexisting phases to the average composition specified in experiments, and that no other methods suitable for low-yield proteins currently provide all three. Further, we proved mathematically that ATRI can, in principle, resolve the composition of condensates containing a potentially large number of distinct molecular components (Supplementary Note 12). Whether partitioning of a particular species can be resolved is determined primarily by the sensitivity and precision of the dilute-phase detection method used (Supplementary Fig. 14). We emphasize that ATRI is agnostic to the choice of dilute-phase detection method(s). This flexibility allows experimenters to select or combine established analytic approaches best suited for the molecules used. Although we found UV–vis spectroscopy useful for analysis of dilute protein–RNA mixtures in this work (Figs. 4 and 5), ATRI is not restricted to this mode of spectroscopy. In particular, liquid chromatography tandem mass spectrometry (LC–MS/MS) is well suited for quantifying proteins and PTMs in complex mixtures with many components^75^. By combining absolute quantification of proteins by MS western with UV–vis to characterize the composition of the dilute phase, we used ATRI to resolve the concentrations of five macromolecular solutes, including RNA and several similar protein constructs (Fig. 5). Although the practical limits to component number are to be established in future work, a preliminary scaling analysis (Supplementary Fig. 15 and Supplementary Note 16) suggests that ATRI is suitable for resolving the composition of condensates with as many as 40 components, potentially bringing reconstitution of cellular condensates with dozens of components within reach^8–10^.

In future applications of QPI and ATRI to condensates with optical contrast, there are three classes of practical considerations that must be kept in mind (see expanded discussion in Supplementary Note 17 (refs. ^32,36,37,42,55,56,76–82^)). The first concerns condensate size. Although QPI measures refractive index robustly for droplets over the size range most commonly encountered in reconstitution experiments, systematic errors are incurred due to scattering and gravitational settling for droplets smaller than a few micrometres or larger than a few tens of micrometres, respectively. Further development of fitting routines to account for these physical effects will probably extend the range for reliable measurements^76,83^. The second consideration involves treating the refractive index of a mixture as a linear sum of contributions from its components (equation (2) and Supplementary Note 4). This is a very good approximation for protein solutions over a wide range^36^, although higher-order terms can contribute for some molecules (Extended Data Fig. 5 and Supplementary Note 14). In the cases we checked, we found the linear sum to describe the refractive index of multicomponent mixtures accurately to within a few percent (Supplementary Fig. 4, Extended Data Fig. 6 and Supplementary Note 13). The third consideration is in regard to measurements in cells. Although the QPI analysis presented here is best suited for reconstituted condensates due to the spherical cap shape requirement, QPI and related techniques such as ODT can provide some information on cellular condensates when optical contrast is present^27,28,38,46,84^. However, label-free determination of individual component concentrations in cellular condensates by QPI or other means remains an open challenge. For condensates with sufficient optical contrast, reconstruction of binodals and tie-lines would require knowledge of molecular abundances in individual cells and knowing the coexisting phase. Absent this, phase-based imaging data can still provide a quantitative measure of the average macromolecular mass difference between phases in vivo^27,38,46^, which may be particularly informative in the context of perturbations.

Because composition ultimately influences all other condensate properties and associated cellular functions, we anticipate a central role for ATRI in addressing many pressing biological questions. Measuring condensate composition as the abundance of individual components is systematically varied will reveal the thermodynamic contributions of these molecules to the phase, potentially clarifying the biological function of individual components. We anticipate that correlating composition with other physical properties, such as viscoelasticity, interfacial tension and dielectric constant, will likewise provide insight into condensate function. By providing a ground-truth with which to calibrate fluorophore behaviour, we expect that this method will enable the use of dyes to both follow reactions localized to condensates and to quantitatively probe the chemical environment within condensates. The latter will probably be essential to understand and potentially tune the partitioning of therapeutic drugs into condensates in treatment of diseases such as cancer^26^.

## Methods

### Sample preparation

Recombinant protein constructs used in this work were purified and stored as described previously^6,7^. Constructs^6,7,85^ are given in Supplementary Table 3, and explicit sequences in Supplementary List 1. To induce phase separation, we mixed protein in high-salt storage buffer (300 mM KCl for PGL-3 constructs, 500 mM KCl for TAF15 and FUS constructs) with storage buffer lacking monovalent salt (‘Dilution Buffer’) to reach the desired final salt concentration. Generally, an aliquot of Dilution Buffer was supplemented to 1 mM with fresh dithiothreitol prior to each day’s experiments. For the ternary and five-solute systems, the dilution buffer was also supplemented with poly(A) RNA (#P9403, Sigma). After induction of phase separation, dilute phase was obtained by centrifugation at 20,800*g* for 30 min in a tabletop centrifuge (5417R, Eppendorf) pre-equilibrated at the desired temperature. For control measurements, 10-µm silica microspheres were purchased from Whitehouse Scientific, and glycerol–water mixtures were prepared by weight to the desired refractive index. Bead-containing dispersions were prepared by gently dipping a 10-µl pipette tip into a stock of dry beads, transferring the pipette tip to a 40-µl volume of glycerol–water mixture, and pipette mixing to disperse. Aqueous two-phase systems with PEG-35k (Sigma) and Dextran T500 (Pharmocosmos) were prepared as described previously^45^. BSA was purchased from Sigma and used without further purification.

### Quantitative phase imaging and analysis

QPI measurements were performed using a coherence-controlled digital holographic microscope (Q-Phase, Telight (formerly TESCAN)) based on the set-up in ref. ^35^. Most data were acquired on a Generation-1 (G1) instrument with a tungsten–halogen bulb as light source, although some data were acquired on a Generation-2 (G2) instrument with a 660-nm light-emitting diode as light source. In each case, the holography light source was filtered by a 10-nm bandwidth notch filter centred at 650 nm. All measurements were performed with 40× dry objectives (0.9 numerical aperture (NA), Nikon) except those for SNAP-TAF15(RBD) reported in Fig. 2f and Supplementary Fig. 8, for which 20× dry objectives were used. In all cases, the condenser aperture was set to an NA of 0.30. Immediately following phase separation, ~5 µl of sample was loaded into a temperature-controlled flowcell, sealed with two-component silicone glue Twinsil (Picodent), and allowed to settle under gravity for ~10 min prior to data collection. Flowcells were constructed with a 30 × 24 × 0.17 mm^3^ PEGylated coverslip and a 75 × 25 × 1 mm^3^ sapphire slide as bottom and top surfaces, respectively, using parafilm strips as spacers. Proportional-integral-derivative (PID)-controlled Peltier elements affixed to the sapphire slide enabled regulation of flowcell temperature, as previously described^48^. The sapphire, coverslip and spacers were adhered by heating the assembled flowcell to 50 °C for 2–5 min, then returning to the desired temperature for the first measurement, typically to 20 °C. For each sample, hologram *z* stacks (∆*z* = 0.2 µm, first plane typically near the coverglass surface) were acquired for several fields of view. SophiQ software (Telight) was used to construct amplitude and compensated phase images from the raw holograms. Pixels in 40× phase images are 0.157 µm per side for the G1 system and 0.1234 µm per side for the G2. To aid interpretation by persons with red/green colour perception deficiencies, phase images are displayed using the Ametrine colourmap^86^.

All phase images were analysed in MATLAB using custom code. In each image, the pixel intensity is taken as equal to the local value of the phase profile at the coverslip, Δ*φ*. For each *z* plane, compensated phase images were first segmented to identify individual droplets. To determine the background phase value, *φ*_0_, the image’s pixel intensity histogram was fitted to a Gaussian, and the Gaussian centre taken as *φ*_0_. Pixel intensities *φ* ≥ *n*_sig_*σ*_*φ*_ are considered above threshold, where *σ*_*φ*_ is the standard deviation extracted from the Gaussian fit. Typically, *n*_sig_ = 5. A binary mask was generated with this threshold and individual objects were identified using the MATLAB function bwconncomp.m. For each object, a region of interest slightly larger than the object’s bounding box was fitted twice to phase functions of the form given by equation (1). These phase functions incorporate geometric models for the local thickness of the droplet, defined as the vertical distance between the upper and lower droplet surfaces. First, we fit using the local thickness of a sphere,

$$\begin{array}{l}{H}_{\mathrm{sphere}}\left(x,{y|R},{x}_{c},{y}_{c}\right)\\=\varTheta \left({R}^{2}-{\left(x-{x}_{c}\right)}^{2}-{\left(\;y-{y}_{c}\right)}^{2}\right)\sqrt{{R}^{2}-{\left(x-{x}_{c}\right)}^{2}-{\left(y-{y}_{c}\right)}^{2}},\end{array}$$

to obtain estimates for the parameters Δ*n*, *R*, *x*_*c*_, *y*_*c*_, where *Θ*(*x*) is the Heaviside function. These estimates were then used to initialize a fit to a regularized version of equation (1),

$$\Delta{\varphi }_{\mathrm{reg}}\left(x,y\right)=\frac{2\uppi }{\lambda}\Delta\;n{H}_{\mathrm{cap}}\left(x,{y|R},{x}_{c},{y}_{c},{Z}_{\mathrm{eq}}\right)+{\varphi }_{0}+A\left({Z}_{\mathrm{eq}},R\right),$$

using the local thickness of a spherical cap,

$$\begin{array}{l}{H}_{\mathrm{cap}}\left(x,{y|R},{x}_{c},{y}_{c},{Z}_{\mathrm{eq}}\right)\;=\;\sqrt{{R}^{2}-{\left(x-{x}_{c}\right)}^{2}-{\left(y-{y}_{c}\right)}^{2}}\\\qquad\qquad\qquad\qquad\qquad\quad\left(1+\varTheta \left({Z}_{\mathrm{eq}}^{2}+{\left(x-{x}_{c}\right)}^{2}+{\left(y-{y}_{c}\right)}^{2}-{R}^{2}\right)\right)\\\qquad\qquad\qquad\qquad\qquad\quad\varTheta \left({R}^{2}-{\left(x-{x}_{c}\right)}^{2}-{\left(y-{y}_{c}\right)}^{2}\right)\\\qquad\qquad\qquad\qquad\qquad\quad+{Z}_{\mathrm{eq}}\varTheta \left({R}^{2}-{Z}_{\mathrm{eq}}^{2}-{\left(x-{x}_{c}\right)}^{2}-{\left(y-{y}_{c}\right)}^{2}\right).\end{array}$$

The regularization is given by $A\left({Z}_{\mathrm{eq}},R\right)={A}_{0}{\left({Z}_{\mathrm{eq}}-R\right)}^{2}\varTheta \left({Z}_{\mathrm{eq}}-R\right)$ with *A*_0_ = 10^5^ and *φ*_0_ fixed at the value obtained from the pixel intensity histogram fit. After all *z* planes were processed, the objects were tracked through *z* using track.m (https://site.physics.georgetown.edu/matlab/index.html). For each tracked object, the representative fit parameters are taken as those from the fit with the highest adjusted *R*^2^ value, which are typically in the plane acquired nearest to the equatorial plane of a given droplet (Extended Data Fig. 1). The particle list was then automatically filtered for fit quality (typically retaining only adjusted *R*^2^ values > 0.95) and overlap with dead pixels on the detector. Droplets with irregular wetting or that are not isolated in *z* (that is, are situated beneath other droplets in solution) are removed manually following inspection of the raw data.

### ODT

ODT measurements were performed using a custom-built microscope based on a Mach–Zehnder interferometer that enabled the acquisition of multiple complex optical fields from various incident angles, as previously described^87,88^. Briefly, a 532-nm solid-state laser beam (Torus, Laser Quantum) was divided into reference and sample beams by a 2 × 2 single-mode fibre-optic coupler. The sample beam illuminated the specimen mounted on a custom-made inverted microscope stage through a tube lens (focal length, 175 mm) and a high-NA objective lens (63×, water immersion, 1.2 NA, Carl Zeiss). A dual-axis Galvano mirror (GVS012/M, Thorlabs) placed in the conjugate plane of the sample was used to scan 150 different incident angles. The scattered light from the sample was collected by a high-NA objective lens (100×, oil immersion, 1.3 NA, Carl Zeiss). This scattered light interfered with the reference beam at the image plane to form spatially modulated holograms, which were recorded using a CMOS camera (FL3-U3-13Y3M-C, FLIR Systems).

To retrieve the complex optical fields, the resulting holograms were processed using a Fourier-transform-based field retrieval algorithm. These fields were then used to reconstruct the three-dimensional (3D) refractive index distribution of the samples via the Fourier diffraction theorem under the first-order Rytov approximation^89,90^. The refractive index we report for individual droplets is computed as the mean refractive index value of voxels at the droplet centre plane. Additional technical details on the reconstruction procedure are available in previous literature^10,91^.

### Confocal fluorescence microscopy and analysis

Confocal imaging was performed on an inverted Zeiss LSM 880 point-scanning confocal microscope with a 40× water-immersion objective (1.2 NA, C-Apochromat, Zeiss) at room temperature. We note that this highly corrected objective is designed for 3D imaging of aqueous samples and has the highest NA compatible with water immersion. We preferred water as an immersion medium despite the higher NA achievable with oil immersion to reduce imaging artefacts from a refractive-index mismatch between the immersion medium and the sample (primarily the dilute phase with a refractive index close to water). mEGFP was excited with a 488-nm argon laser and emission detected with a 32-channel GaAsP photomultiplier tube (PMT) array set to accept photon wavelengths between 499 and 569 nm. Alexa Fluor 546 (AF546) was excited with a 561-nm diode-pumped solid-state laser and emission detected between 570 and 624 nm with the same spectral PMT array. For both fluorophores, the confocal pinhole diameter was set to 39.4 µm, corresponding to 0.87 and 0.96 Airy units for mEGFP and AF546, respectively. For each field of view, scanning was performed with a lateral pixel size of 0.415 µm and *z* stacks acquired with a spacing of 0.482 µm. We note that the choice of a comparatively large lateral pixel size was made (1) to reduce the overall laser dosage, (2) to reduce the image acquisition time, and (3) to give a voxel aspect ratio close to 1 with our axial step-size (Extended Data Fig. 2).

All confocal fluorescence images were analysed in MATLAB using custom code. Partition coefficients of fluorescently labelled species into condensates are estimated on the basis of the fluorescence intensity along a line-scan through the droplet centre. The analysis pipeline begins with determining the location of each condensate and an appropriate line-scan orientation angle. To determine lateral positions of condensates in each field of view, a *z* plane was selected slightly above the coverglass such that even small droplets appeared bright. Following convolution with a 2D Gaussian (*σ*_*x*_ = *σ*_*y*_ = 0.5 pixels) to suppress shot noise, a threshold of *I*_thresh_ = max(*I*(*x*,*y*))/2 was applied to obtain a binary mask. The lateral positions and approximate sizes of objects were determined from the mask with bwconncomp.m. Only the largest ~120 objects for each condition were analysed further. For each object, partition coefficients were calculated using the *z* plane for which the mean intensity in a 5-pixel-radius disk concentric with the object was greatest. Line-scans were 51 pixels long, concentric with the object and averaged over a width of 3 pixels. Suitable line-scan orientations were determined in a semiautomated manner by superimposing reference lines rotated through 15° increments on each object and manually selecting an orientation that best avoided neighbouring objects. Objects for which no suitable line-scan orientation could be found were discarded.

Line-scans for each droplet were automatically subdivided into three domains, corresponding to pixels in the dilute phase, the condensed phase or the exclusion zone. The positions of the left and right dilute/condensed interfaces are estimated as those at which the intensity profile reaches its half-maximal value above detector background *I*_bkgd_ (see below). To reduce artefacts stemming from the finite point-spread function of the microscope, pixels within an exclusion zone, defined as the greater of 1 pixel or *l*_EZ_ = 1.22*λ*/(2NA) on either size of the half-maximum, were excluded from the analysis (Extended Data Fig. 2). We estimate the decay length scale of the axial point-spread function for light emitted at the peak fluorescence wavelength *λ*_em_ as^92^

$${l}_{z}=\frac{0.88{\lambda }_{\mathrm{em}}}{{n}_{\mathrm{immersion}}-\sqrt{{n}_{\mathrm{immersion}}^{2}-{\left({\mathrm{NA}}\right)}^{2}}},$$

where *n*_immersion_ = 1.333 is the refractive index of the immersion medium (water). Using *λ*_em_ = 510 nm for mEGFP (www.fpbase.org (ref. ^93^)) and NA = 1.2, this gives a theoretical axial PSF resolution of 596 nm. The remaining profile pixels outside the droplet were averaged to give *I*_dil_, while the profile pixels inside are averaged to give *I*_cond_. The partition coefficient for each object was calculated according to

$$P=\frac{{I}_{\mathrm{cond}}-{I}_{\mathrm{bkgd}}}{{I}_{\mathrm{dil}}-{I}_{\mathrm{bkgd}}},$$

where *I*_bkgd_ is the average of all pixels in a background image acquired immediately following the fluorescence *z* stack. Background images were acquired with the light source blocked to measure the contribution of detector noise to the signal.

### Bulk refractometry

The data in Fig. 1k were acquired at *λ* = 653.3 nm and 21 °C with a DSR-L multiwavelength refractometer (Schmidt + Haensch) using a 200-µl sample volume. All other bulk refractive index measurements were acquired at *λ* = 589.3 nm with a J457 refractometer (Rudolph Research Analytical). The refractive index of glycerol/water mixtures was adjusted from *λ* = 589.3 to 650 nm using empirical dispersion relations for distilled water, *n*_water_(*λ*) (ref. ^94^), and glycerol, *n*_g__lycerol_(*λ*) (ref. ^95^) according to

$${n}_{\mathrm{mix}}\left(\lambda \right)={w}_{\mathrm{glycerol}}{n}_{\mathrm{glycerol}}\left(\lambda \right)+\left(1-{w}_{\mathrm{glycerol}}\right){n}_{\mathrm{water}}\left(\lambda \right).$$

The glycerol weight fraction in the mixture was calculated from the refractive index measurement of the mixture at 589.3 nm as

$${w}_{\mathrm{glycerol}}={\left.\left({n}_{\mathrm{mix}}\left(\lambda \right)-{n}_{\mathrm{water}}\left(\lambda \right)\right)/\left({n}_{\mathrm{glycerol}}\left(\lambda \right)-{n}_{\mathrm{water}}\left(\lambda \right)\right)\right|}_{\lambda =589.3}.$$

### Bead porosity models

The two models used to account for the porosity *p* of the silica microspheres (Fig. 1j and Supplementary Fig. 6) are a weighted linear sum, $\Delta n=p{n}_{\mathrm{silica}}+\left(1-p\right){n}_{\mathrm{water}}$ (simple model), and a more detailed model,

$$\Delta n={\left(\frac{1+2\left(1-p\right)f\left({n}_{\mathrm{silica}}\right)+2{pf}\left({n}_{\mathrm{mix}}\right)}{1-\left(1-p\right)f\left({n}_{\mathrm{silica}}\right)+{pf}\left({n}_{\mathrm{mix}}\right)}\right)}^{1/2}-{n}_{\mathrm{mix}}$$

based on the Lorentz–Lorenz relation^96^, wherein

$${\rm{p}}=\frac{f\left({n}_{\mathrm{silica}}\right)-f\left({n}_{\mathrm{microsphere}}\right)}{f\left({n}_{\mathrm{silica}}\right)-f\left({n}_{\mathrm{glycerol{-}water\,mixture}}\right)}$$

with $f\left(n\right)\equiv \left({n}^{2}-1\right)/\left({n}^{2}+2\right)$. For comparison, reference values for fused silica were taken from ref. ^97^.

### Calculation of d*n*/d*c*, $\bar{{\boldsymbol{v}}}$ and polymer volume fraction

The refractive index increment and partial specific volume were estimated for each protein construct using the calculator tool within SEDFIT^32^ and the protein sequences listed in the Supplementary Information. The partial specific volume of 0.5773 ml g^−1^ for poly(A) RNA was estimated using consensus volumes per base from ref. ^98^ and assuming a typical chain length of 500 bases. The partial specific volumes of 0.8321 and 0.6374 ml g^−1^ for PEG-35k and Dextran-500k, respectively, were taken from ref. ^45^. The polymer volume fraction in the dense phase (Figs. 4g and 5g) for polymer *i* is given by ${\phi }_{i}^{II}={c}_{i}^{II}\bar{v}_{i}$.

### Estimation of capillary length

We estimate the capillary length for a droplet-forming system as ${l}_{c}={\left(\gamma /\Delta \rho g\right)}^{1/2}$, where *γ* and Δ*ρ* are the interfacial tension and mass density differences between the two phases, respectively, and *g* is the acceleration due to gravity. The range of *γ* values we explored was informed by the values measured in ref. ^99^.

### Calculations for ageing systems

The volumes of individual ageing condensates (Fig. 3c,f) were calculated assuming the shape of a spherical caps as $V={\frac{4}{3}\uppi}R^{3}[1-\frac{1}{4}\left(2+\frac{{Z}_{\mathrm{eq}}}{R}\right){\left(1-\frac{{Z}_{\mathrm{eq}}}{R}\right)}^{2}]$. The number of molecules in each condensate (Fig. 3d,g) was calculated as $N\left(t\right)=c\left(t\right)V\left(t\right)$. Given *c*(*t*), the relative volume change expected if $N\left(t\right)=N\left(0\right)$ is given by $V\left(t\right)/V\left(0\right)=c\left(0\right)/c\left(t\right)$ (Fig. 3f, black line).

### UV–vis spectroscopy

Absorption spectra of dilute-phase and reference samples were collected on an NP-80 spectrophotometer (IMPLEN). All spectra were acquired at room temperature over *λ* ∈ [200 nm, 900 nm]. Depending on signal strength, spectra were acquired with a pathlength of 0.07 mm, 0.67 mm or 10 mm. All absorption spectra were converted to absorbance units (AU) appropriate to a 10-mm pathlength prior to analysis. For each raw spectra $\widetilde{S}\left(\lambda \right)$, a linear fit on *λ* ∈ [550 nm, 750 nm] was used to determine a baseline correction, *S*_BL_(*λ*). Corrected spectra are given by $S\left(\lambda \right)=\widetilde{S}\left(\lambda \right)-{S}_{\mathrm{BL}}\left(\lambda \right)$. At least three replicate spectra were acquired for each condition and averaged following baseline correction to give the final representative spectra. The uncertainty in the spectra at each wavelength was estimated as the standard deviation of the corrected replicates.

For FUS/RNA ternary mixtures, dilute-phase spectra were demixed (Fig. 4b) into a weighted sum of three contributions

$${S}_{\mathrm{dil}}\left(\lambda \right)={p}^{\mathrm{I}}{S}_{\mathrm{p}}\left(\lambda \right)+{r}^{\mathrm{I}}{S}_{\mathrm{r}}\left(\lambda \right)+{p}^{\mathrm{I}}{r}^{\mathrm{I}}{a}_{\mathrm{int}}{S}_{\mathrm{int}}\left(\lambda \right),$$

where *p*^I^ and *r*^I^ are the protein and RNA concentrations in the dilute phase. *S*_p_ and *S*_r_ are reference spectra for protein and RNA, respectively. The final term captures the effect of protein–RNA interactions on the absorbance of a mixture, which could physically stem from binding-induced changes in extinction coefficients. A reference spectrum for the interaction was calculated from the spectrum of a protein–RNA mixture of known composition (*p*,*r*) in the one-phase regime according to ${S}_{\mathrm{int}}=S\left(p,r\right)-p{S}_{\mathrm{p}}-r{S}_{\mathrm{r}}$. The parameter *α*_int_ captures the approximately linear increase of *S*_int_ with *p* (Extended Data Fig. 4). The same value of *α*_int_ was used to demix all dilute-phase spectra. The dilute-phase concentrations *p*^I^,*r*^I^ were therefore the only free parameters for the demixing.

### Refractive index cross-term calculation

The relevant theory and the calculation are presented in detail in Supplementary Note 14. Briefly, the calculation began with the construction of the excess absorption spectra of a ‘homogeneous test mixture’ of FUS and RNA with *c*_FUS_ = *c*_RNA_ = 1 mg ml^−1^ using the interaction term and normalized interaction spectra determined by UV–vis spectroscopy. This excess absorption spectrum was converted to excess extinction and transformed from wavelength space to frequency space. The frequency-dependent excess extinction was then fitted to a model for the dissipation of two driven linearly damped linear oscillators (two-oscillator model) to give an analytic approximation to the spectrum over all frequencies. The real component of the excess optical response function was calculated from a Kramers–Kronig relation via symbolic integration of a normalized version of the fitted two-oscillator model spectrum in MATLAB using int.m with the ‘PrincipalValue’ flag set to true. After rescaling back to the original units and converting back to wavelength space, the value of the refractive cross-term coefficient at the 650-nm wavelength used for QPI measurements was calculated from the excess refractive index as ${\nu }_{{ij}}\left(650\,\text{nm}\right)\equiv \frac{\mathrm{d}^{2}n}{\mathrm{d}{c}_{i}{\mathrm{d}}{c}_{j}}\left(650\,\text{nm}\right)=\frac{{n}_{\mathrm{excess}}\left(650\,\text{nm}\right)}{{c}_{\mathrm{FUS}}{c}_{\mathrm{RNA}}}$.

### Composition calculation for ternary mixtures with a quadratic refractive index model

A derivation of the ATRI theory for a quadratic refractive index model is presented in Supplementary Note 13. To calculate the composition of FUS/RNA condensates using a quadratic refractive index model in Extended Data Fig. 6, the dense-phase composition was calculated by solving the system of equations given by $${c}_{2}^{\mathrm{II}}={m}_{\mathrm{TL}}{c}_{1}^{\mathrm{II}}+{b}_{\mathrm{TL}}$$ together with $${c}_{2}^{\mathrm{II}}=\frac{{c}_{2}^{\mathrm{II},\max }-r{c}_{1}^{\mathrm{II}}}{1+{\widetilde{\upsilon }}_{12}{c}_{1}^{\mathrm{II}}}$$, where $${m}_{\mathrm{TL}}=\frac{{\bar{c}}_{2}-{c}_{2}^{\mathrm{I}}}{{\bar{c}}_{1}-{c}_{1}^{\mathrm{I}}}$$ and $${b}_{\mathrm{TL}}={\bar{c}}_{2}-{m}_{\mathrm{TL}}{\bar{c}}_{1}$$ are the slope and *y*-intercept of the tie-line, respectively, and $${c}_{2}^{\mathrm{II},\max }\equiv \Delta n+{c}_{1}^{\mathrm{I}}\frac{{\mathrm{d}n}}{\mathrm{d}{c}_{1}}$$$$+{c}_{2}^{\mathrm{I}}\frac{{\mathrm{d}n}}{\mathrm{d}{c}_{2}}+{c}_{1}^{\mathrm{I}}{c}_{2}^{\mathrm{I}}\frac{\mathrm{d}^{2}n}{\mathrm{d}{c}_{1}\mathrm{d}{c}_{2}}$$, $$r\equiv \frac{{\mathrm{d}n}}{\mathrm{d}{c}_{1}}/\frac{{\mathrm{d}n}}{\mathrm{d}{c}_{2}}$$ and $${\widetilde{\upsilon }}_{12}\equiv \frac{\mathrm{d}^{2}n}{\mathrm{d}{c}_{1}\mathrm{d}{c}_{2}}/\frac{{\mathrm{d}n}}{\mathrm{d}{c}_{2}}$$. As the second equation for $${c}_{2}^{\mathrm{II}}$$ is non-linear, the system was solved numerically in MATLAB. For each of the tie-lines in Fig. 4, $${c}_{1}^{\mathrm{II}}$$ was calculated first by solving $$\frac{{c}_{2}^{\mathrm{II},\max }-r{c}_{1}^{\mathrm{II}}}{1+{\widetilde{\upsilon }}_{12}{c}_{1}^{\mathrm{II}}}={m}_{\mathrm{TL}}{c}_{1}^{\mathrm{II}}+{b}_{\mathrm{TL}}$$ using the non-linear solver fsolve.m. Subsequently, $${c}_{2}^{\mathrm{II}}$$ was determined through the tie-line equation.

The magnitude of the systematic error incurred when assessing FUS/RNA condensate composition in terms of a linear refractive index model relative to a quadratic one was calculated as $\delta {c}_{i}^{\mathrm{sys}}=\left|\frac{{c}_{i}^{\mathrm{quad}}-{c}_{i}^{\mathrm{lin}}}{{c}_{i}^{\mathrm{quad}}}\right|$, where the superscripts ‘quad’ and ‘lin’ specify the refractive index model used to obtain the concentration value.

### Dilute-phase RNA concentration for a five-solute system

Here, the RNA concentration in the dilute phase was determined from UV–vis absorption spectra *S*^I^(*λ*) of the dilute phase at 260 nm after accounting for contributions from protein solutes, the AF546 dye on the TAF15 construct, a UV-active contaminant population in the TAF15 stock, and interactions between the RNA and the RNA-binding proteins present according to ${c}_{\mathrm{RNA}}^\mathrm{I}=\frac{{S}^\mathrm{I}\left(260\,\text{nm}\right)-{\sum }_{i=1}^{6}{\varepsilon }_{i}\left(260\,\text{nm}\right){c}_{i}^\mathrm{I}}{{\varepsilon }_{\mathrm{RNA}}\left(260\,\text{nm}\right)+{\sum }_{i=1}^{4}{a}_{\mathrm{int}}{c}_{i}^\mathrm{I}}$, where *ε*_*i*_ is the extinction coefficient of species *i*, ${c}_{i}^{\mathrm{I}}$ is the dilute-phase concentration of species *i*, and *i* indexes the non-RNA components considered according to 1 = FL-FUS-mEGFP, 2 = MBP-FUS-mEGFP, 3 = shortFUS-mEGFP, 4 = SNAP-TAF15(RBD) (with and without AF546 dye), 5 = AF546 dye, 6 = effective UV-active contaminant in the SNAP-TAF15(RBD) stock. The central estimate of ${c}_{\mathrm{RNA}}^{\mathrm{I}}$ and its uncertainty were computed as the mean and standard deviation of the population of estimates obtained through Monte Carlo simulations^100^ of the preceding equation using experimentally constrained Gaussian distributions for the measured quantities *S*^I^ and ${c}_{i}^{\mathrm{I}}$ (Extended Data Fig. 8).

For $i\in \left\{\mathrm{1,2,3,4}\right\}$, the ${c}_{i}^{\mathrm{I}}$ of the corresponding population was determined from MS. For the AF546 dye, we set ${c}_{5}^{\mathrm{I}}={f}_{\mathrm{AF}}{c}_{4}^{\mathrm{I}}$, where *f*_AF_ is the fraction of dye-bound SNAP-TAF15(RBD) molecules in the stock solution. This expression is valid in the limit that covalent attachment of the AF546 dye does not significantly perturb the partitioning of SNAP-TAF15(RBD) relative to the construct without dye. *f*_AF_ was determined from the ratio of the dye concentration ${c}_{\mathrm{AF}}^{\mathrm{ref}}$ measured by UV–vis in a homogeneous reference sample formed by dilution of the SNAP-TAF15(RBD) stock solution to the protein concentration ${c}_{\mathrm{TAF}}^{\mathrm{ref}}$ in the SNAP-TAF15(RBD) population measured by MS of the same reference sample. We define the effective concentration of UV-active contaminants in a stock solution as the difference between the protein concentration inferred from absorbance at 280 nm in a reference sample to that measured by MS. For the SNAP-TAF15(RBD), the equation used is ${c}_{\mathrm{TAF}-{\mathrm{cont}}}^{\mathrm{ref}}=\frac{{S}_{280}-{\varepsilon }_{\mathrm{TAF}}{c}_{\mathrm{TAF}}^{\mathrm{ref}}-{\varepsilon }_{\mathrm{AF}546}{c}_{\mathrm{AF}546}^{\mathrm{ref}}}{{\varepsilon }_{\mathrm{TAF}-{\mathrm{cont}}}}$. We set ${\varepsilon }_{\mathrm{TAF}-{\mathrm{cont}}}={\varepsilon }_{\mathrm{TAF}}$ for convenience and note that the exact choice is ultimately irrelevant because ${c}_{\mathrm{TAF}-{\mathrm{cont}}}^{\mathrm{I}}$ appears in the expression for ${c}_{\mathrm{RNA}}^{\mathrm{I}}$ only as the product ${\varepsilon }_{\mathrm{TAF}-{\mathrm{cont}}}{c}_{\mathrm{TAF}-{\mathrm{cont}}}^{\mathrm{I}}$, which is uniquely determined. Within the uncertainty of the measurements, this difference was zero for the reference sample from the FUS stock solution but non-zero for the reference sample from the SNAP-TAF15(RBD) stock solution (Supplementary Fig. 13). For the effective contaminants from the SNAP-TAF15(RBD) stock, the concentration in the dilute phase was estimated as ${c}_{6}^{\mathrm{I}}={\bar{c}}_{6}$, where ${\bar{c}}_{6}$ is the average concentration in the demixed system. This expression is valid in the limit where the partition coefficient of the effective contaminant species is close to 1.

### MS

Proteins were enzymatically digested and analysed by LC–MS/MS on an RSLC system UltiMate 3000 series coupled with a Q Exactive HF mass spectrometer (Thermo Fisher Scientific). Molar abundances of quantotypic peptide were calculated as described previously^70^. Each proteotypic peptide was quantified independently. To calculate the molar abundance of proteins, the molar abundances of the corresponding peptides were averaged. Further details can be found in Supplementary Note 15.

### Statistics and reproducibility

Individual droplets were excluded from quantitative analysis when they did not meet the physical assumptions of the analysis. As described above and in Supplementary Note 17, droplets were excluded from QPI analysis if the shape inferred from the phase image was not well described by a spherical cap, for instance due to irregular wetting of the coverslip or the presence of an additional object in solution directly above the droplet of interest. Similarly, droplets were excluded from ODT analysis if their tomographic reconstructions were strongly impacted by artefacts due to their position near the edge of the field of view. Droplets with volumes smaller than 0.1194 µm^3^ (equivalent to fewer than 100 voxels) were also excluded from ODT analysis to avoid misclassification of small segmented regions of background noise as droplets. As samples G and H in Supplementary Fig. 8 were both prepared in the presence of a small number of polystyrene microspheres with radii near 2 µm, we additionally restricted the ODT analysis of these samples to objects with volumes smaller than 15 µm^3^ to ensure that the few microspheres present in our tomograms were excluded. Droplets were excluded from final fluorescence analysis when they were too small relative to the axial point-spread function for the fluorescence intensity estimated from interior pixels to reach the level observed in larger droplets (see also Extended Data Fig. 2). Otherwise, data were not excluded from the analyses.

To assess the statistical significance of a correlation coefficient *r*_obs_ computed from a set of *N*_obs_ paired experimental measurements, we determined one-sided *P* values from Monte Carlo simulations^100^. Briefly, we used rand.m in MATLAB to randomly draw *N*_obs_ *x* and *y* values from a uniform distribution. From this set of simulated data, we computed a correlation coefficient *r*_sim_ between the randomly paired *x* and *y* values. We repeated this procedure *N*_iter_ = 100,000 times to generate a probability density distribution $\rho \left({r}_{\mathrm{sim}}|{N}_{\mathrm{obs}}\right)$ of *r*_sim_ values given the sample size *N*_obs_. We then calculated the probability of encountering by chance a correlation at least as large as the correlation found experimentally. We compute this as the fraction of the simulations for which *r*_sim_ ≥ *r*_obs_. In the limit that *N*_iter_ → ∞, this fraction is equivalent to

$$P=\frac{{\int }_{{r}_{\mathrm{obs}}}^{1}\rho \left({r}_{\mathrm{sim}}|{N}_{\mathrm{obs}}\right){\mathrm{d}}{r}_{\mathrm{sim}}}{{\int }_{-1}^{1}\rho \left({r}_{\mathrm{sim}}|{N}_{\mathrm{obs}}\right){\mathrm{d}}{r}_{\mathrm{sim}}}$$

that is, a one-sided *P* value. To assess the uncertainty incurred by using a finite number of simulations, we generated a *P*-value distribution by repeating the p-value calculation procedure 50 times. The *P* value we report is the mean from this *P*-value distribution. For cases where the standard deviation of the distribution is larger than 10% of the mean, we report the standard deviation as well.

No statistical method was used to predetermine sample size. The experiments were not randomized. The investigators were not blinded to allocation during experiments and outcome assessment.

### Reporting summary

Further information on research design is available in the Nature Portfolio Reporting Summary linked to this article.

## Online content

Any methods, additional references, Nature Portfolio reporting summaries, source data, extended data, supplementary information, acknowledgements, peer review information; details of author contributions and competing interests; and statements of data and code availability are available at 10.1038/s41557-025-01928-3.

## Supplementary information

Supplementary Information Supplementary Video 1 caption, Notes 1–17, Figs. 1–15, Tables 1–3, List 1 and Refs. 1–56.

Reporting Summary

Supplementary Video 1 **Aging timecourse of untagged PGL-3 condensates**. Quantitative phase imaging of aging timecourse for untagged PGL-3 condensates reconstituted in 75 mM KCl at 25 °C.

Supplementary Table 1 **Compositions of biomolecular condensates in vitro**. ^a^ Uncertainty represents standard deviation from a population of at least 100 individual condensates. ^b^ Fraction of the condensed phase volume occupied by protein, $\phi \equiv {c}_{{Cond}}\bar{v}$; $\bar{v}\approx 0.75$ mL/g for PGL3 constructs and $\bar{v}\approx 0.71$ mL/g for TAF15 and FUS constructs. ^c^ c_Dil_ = 1.97 ± 0.09 µM, M_w_ = 62.92 kDa^55^; ^d^ c_Dil_ = 4.87 ± 0.48 µM, M_w_ = 80.38 kDa^55^.

Supplementary Table 2 **Peptides for MS Western quantification by the method of PRM**.

Supplementary Table 3 **Protein expression constructs used**.

## Source data

Source Data Fig. 1 Statistical source data.

Source Data Fig. 2 Statistical source data.

Source Data Fig. 3 Statistical source data.

Source Data Fig. 4 Statistical source data.

Source Data Fig. 5 Statistical source data.

Source Data Extended Data Fig. 1 Statistical source data.

Source Data Extended Data Fig. 2 Statistical source data.

Source Data Extended Data Fig. 3 Statistical source data.

Source Data Extended Data Fig. 4 Statistical source data.

Source Data Extended Data Fig. 5 Statistical source data.

Source Data Extended Data Fig. 6 Statistical source data.

Source Data Extended Data Fig. 7 Unprocessed gel image.

Source Data Extended Data Fig. 8 Statistical source data.

Source Data Extended Data Fig. 9 Statistical source data.

Source Data Extended Data Fig. 10 Statistical source data.

## Data Availability

Sequences for the proteins used in this work are provided in the Supplementary Information. MS data are publicly available on the Edmond data repository (10.17617/3.PCTHKT)^101^ hosted by the Max Planck Society. Source data, including the dense-phase and dilute-phase compositions measured for the multicomponent protein/RNA systems described in this work, are provided with this paper. Further materials, such as raw microscopy images, are available upon reasonable request to the corresponding authors. Source data are provided with this paper.

## Code availability

In this study, QPI data were analysed with custom code written in MATLAB. An archived copy of the relevant MATLAB code (v1.0.0) is available from Zenodo via 10.5281/zenodo.16287552 (ref. ^102^). For testing the code, a minimal sample dataset of PEG/dextran droplets imaged by QPI is available from Zenodo via 10.5281/zenodo.15866525 (ref. ^103^). The MATLAB code used for ODT reconstruction (v.1.0.0) is available from Zenondo via 10.5281/zenodo.8341299 (ref. ^104^).
