# Supplementary material for: A label-free method for measuring the composition of multicomponent biomolecular condensates
Source: Nat Chem. 2025 Sep 3;17(12):1891–902. doi: 10.1038/s41557-025-01928-3 (PMC12669041; doi:10.1038/s41557-025-01928-3)
Supplement: Supplementary file 1 — Supplementary Video 1 caption, Notes 1–17, Figs. 1–15, Tables 1–3, List 1 and Refs. 1–56. [file 41557_2025_1928_MOESM1_ESM.pdf]

nature chemistry

Supplementary information

https://doi.org/10.1038/s41557-025-01928-3

A label-free method for measuring the composition of multicomponent biomolecular condensates

In the format provided by the authors and unedited

TABLE OF CONTENTS

- **Supplementary Videos** (1)
  1. Aging untagged PGL-3 condensates monitored by QPI

- **Supplementary Notes** (17)
  1. Limitations of existing label-free approaches to condensate composition determination
  2. Requirements of bulk measurements
  3. Straight-line optical path approximation for QPI
  4. Linear approximation to refractive index of mixtures
  5. Measuring droplet shape on flat surfaces
  6. Measurement of c_{DiI}
  7. Conversion from Δn to concentration
  8. Temperature-dependent phase behavior
  9. Alternative interpretations of the refractive index increase during aging
  10. Implications of protein ejection from condensates during aging
  11. Requirement that tie-lines are straight
  12. Derivation of condensate composition for multi-component systems (linear ATRI)
  13. Condensate composition for multi-component systems with a quadratic model for refractive index (non-linear ATRI)
  14. Calculation of excess extinction in multi-component mixtures
  15. Extended description of absolute protein quantification by mass spectrometry
  16. Compositional uncertainty in multi-component condensates
  17. Physical requirements and associated limitations of QPI and ATRI

- **Supplementary Figures** (15)
  1. Straight-line optical path approximation for QPI Analysis
  2. Impact of solute concentration scale on assessment of refractive index linearity
  3. Refractive index increment of poly(A) RNA at 589 nm
  4. Validation of the linear sum approximation for homogeneous PEG/dextran mixtures
  5. Estimate of capillary length for biomolecular condensates
  6. Silica bead measurements and porosity
  7. Temperature-dependent optical constants
  8. Impact of environmental conditions on Δn for SNAP-TAF15(RBD) condensates
  9. RNA absorbance is sensitive to solution conditions
  10. Examples of physically-permissible tie-lines for an associative ternary mixture
  11. Identification of protein species by mass spectrometry
  12. Contribution of dye to AF546-SNAP-TAF15(RBD) absorption spectra
  13. Estimation of UV-active contaminant abundance in SNAP-TAF15(RBD) stock solution via Monte Carlo simulation
  14. Minimum partition coefficient resolvable by ATRI
  15. Scaling analysis of dense-phase uncertainty in multi-component systems

- **Supplementary Tables** (3)
  1. Compositions of biomolecular condensates reconstituted as binary mixtures
  2. Peptides for MS Western quantification by the method of PRM
  3. Protein expression constructs used

- **Supplementary Lists** (1)
  1. List of protein sequences used

- **Supplementary References** (56)
  Works cited in Supplementary Information

SUPPLEMENTARY VIDEOS

Video S1: Aging timecourse of untagged PGL-3 condensates. Quantitative phase imaging of aging timecourse for untagged PGL-3 condensates reconstituted in 75 mM KCl at 25 °C.

SUPPLEMENTARY NOTES

Supplementary Note 1: Limitations of existing label-free approaches to condensates composition determination

Here we briefly expand upon limitations to existing label-free approaches by which the composition of condensates may be measured. For example, traditional bulk approaches like ultra-violet (UV) absorption[1,2] and thermogravimetric analysis[3] require condensate dissolution or destruction and impose sample requirements that are often inaccessible with the modest yields obtained by recombinant expression and purification of endogenous cellular condensate components (**Supplementary Note 2**). Though confocal Raman spectroscopy enables measurements of intact condensates[4,5], the experimental requirements of high laser exposure or nanoparticles for surface-enhancements may alter condensate dynamics, and a calibration sufficient to resolve multi-component composition has yet to be demonstrated. Microscopy-based volume measurements of dense-phase encapsulated in water-in-oil emulsion droplets have recently been used to measure dense-phase concentrations by applying the lever rule for binary mixtures[6,7] and are possible without fluorescent labels but cannot resolve multiple components. Quantitative phase imaging (QPI) is an attractive label-free alternative, as the refractive index difference Δn it measures between a micron-sized droplet and the surrounding solution is directly related to compositional differences[8–10]. While Δn can be converted to a concentration difference for binary systems[8,9], ambiguity in how to distribute Δn between different molecules has so far made it impossible to distinguish concentrations of multiple species.

Supplementary Note 2: Requirements of bulk measurements

Ideally, one would measure dn/dc and validate linearity over the entire range of $c \in [0, c_{Cond}]$. However, for proteins that readily form biomolecular condensates, a large subset of this range lies in the miscibility gap, $c \in (c_{Dil}, c_{Cond})$, and so homogeneous solutions with these concentrations *cannot be prepared*, at least not at the desired salt, pH, temp, etc. This leaves the formal possibility of assessing dn/dc on $c \in [0, c_{Dil}]$ or for $c > c_{Cond}$. We first discuss issues for measurements above c_{Cond} and subsequently for those below c_{Dil}.

Supplementary Note 2.1: Measurements above c_{Cond}

Excessive material requirements make the high concentration range impractical for many purified full-length proteins. To see this, we estimate the mass of purified protein required for a potential measurement.

To prepare samples of volume V_{sample} at N concentrations spaced Δc apart starting at c_1 would require a total mass of

$$m_{tot} = V_{sample}\left[c_1 N + \Delta c \frac{(N-1)N}{2}\right] = V_{sample} c_1 N \left[1 + \frac{\Delta c}{c_1}\frac{(N-1)}{2}\right] \quad (2.1)$$

For $V_{sample} = 0.1$ mL, $c_1 = c_{Cond} \approx 300$ mg/ml, $\Delta c = 10$ mg/ml, and $N = 3$ measurements, $m_{tot} = 93$ mg. For comparison, the yield of FUS-mEGFP following a typical purification is ~2 mg per liter of insect cell culture (24 aliquots × 60 µM × 80 kDa = 2.3 mg). While perhaps not impossible, it is highly impractical and expensive to work with on the order of 93/2.3 = 40 L of culture. Also, typical columns are not designed for such a high material load, and purification would have to proceed in batches in series, introducing potential batch effects. The low yield and batch effects introduced by serial purification make preparation of protein samples at the high concentrations and large volumes required for bulk measurements of refractive index expensive and highly impractical in most cases.

These issues have been overcome in some limited cases. Specifically, improved yield through overexpression in bacteria and purification under denaturing conditions have enabled isolation of intrinsically disordered protein regions (IDRs) at or near the 100-mg-scale [11–13]. However, these approaches are not generically applicable to full-length proteins, particularly those containing folded domains or post-translational modifications (PTMs). Bacteria lack the machinery required for adding eukaryotic PTMs as well as the chaperones required for proper folding of many larger eukaryotic proteins. Bacterial expression systems are thus not appropriate for proteins with PTMs or that require chaperones. Further, recovery of native-state protein conformations following denaturation during purification is far from guaranteed.

Following removal of chemical denaturants, proteins containing folded domains are prone to form aggregates which may not be readily reversible. Though it appears to be commonly assumed that IDRs recover a native conformational ensemble upon denaturant removal, we are not aware of a study where this equivalence has been demonstrated. The use of denaturing conditions during purification thus requires significant structural characterization and potentially extensive trial-and-error in order to attain pure protein with a native conformational ensemble, particularly when a folded domain is present. Given these limitations, measuring solution refractive index with bulk techniques at concentrations of full-length eukaryotic proteins above c_{Cond} is generally not practical.

Supplementary Note 2.2: Measurements below c_{Dil}

The primary issue with measuring dn/dc from measurements of the refractive index $n(c)$ with $c \in [0, c_{Dil}]$ is that c_{Dil} is often so low that $n(c_{Dil})$ may not be measurably different from $n(0)$. As an example, c_{Dil} for PGL3-mEGFP is below 500 nM [14], corresponding to a mass concentration of ~ 0.05 mg/mL. Using the sequence-based prediction [15] of $dn/dc = 0.1886$ ml/g for this protein at $\lambda = 589$ nm and $T = 25$ °C, the maximal increase in solution refractive index over this range is $n(c_{Dil}) - n(0) = \frac{dn}{dc} c_{Dil} \approx 9 \times 10^{-6}$. This is smaller than the sensitivity of our research-grade digital refractometer, which (only) measures out to five digits.

Given the impossibility of measuring dn/dc at protein concentrations within the miscibility gap, the exceptional sensitivity required to measure it at low concentrations, and the impracticality of measuring it at high concentrations, we find the sequence-based estimates adequate for current purposes.

Supplementary Note 3: Straight-line optical path approximation for QPI

In the context of geometric optics, the path of a light ray transmitted across a material interface deviates by an angle $\delta\theta$ from the path of the incident ray owing to refraction (**Fig. S1a**). Let H be the non-refracted pathlength and $H' = H + \delta H$ be the refracted pathlength of the transmitted ray. We are interested in how the fractional deviation $\delta H/H$ depends on the incident angle θ_1 and the refractive index difference (mismatch) $\Delta n = n_2 - n_1$ between the droplet and the outer phase. Intuitively, we expect the angular deviation $\delta\theta$ to increase with Δn. For concreteness, we assume $n_2 > n_1$ and let θ_2 denote the angle between the refracted path and the droplet surface normal such that $\delta\theta = \theta_1 - \theta_2$.

For the geometry of a sessile droplet on a planar substrate (coverglass) illuminated from above, the refracted ray represents the hypotenuse of a right triangle with the non-refracted path and planar substrate making the legs. Correspondingly, the lengths of the refracted and non-refracted paths are related by the cosine of the angle between them

$$H'\cos\delta\theta = H. \tag{3.1}$$

From this the fractional deviation $\delta H/H$ in the length of the refracted path relative to the non-refracted path can be written as

$$\frac{\delta H}{H} = \frac{1}{\cos\delta\theta} - 1. \tag{3.2}$$

To relate the angular deviation of the refracted path to the refractive indices inside and outside the droplet, we make use of Snell's law

$$n_1\sin\theta_1 = n_2\sin\theta_2. \tag{3.3}$$

Replacing $\theta_2 = \theta_1 - \delta\theta$ and applying a trigonometric identity for the difference of angles, Eq. (3.3) becomes

$$\cos\delta\theta = \frac{n_1}{n_2} + \cot\theta_1\sin\delta\theta. \tag{3.4}$$

Replacing $\sin\delta\theta = \sqrt{1-\cos^2\delta\theta}$ reveals a quadratic equation in $\cos\delta\theta$

$$0 = [1+\tan^2\theta_1]\cos^2\delta\theta - \left[\frac{2n_1}{n_2}\tan^2\theta_1\right]\cos\delta\theta + \left[\left(\frac{n_1}{n_2}\right)^2\tan^2\theta_1 - 1\right]. \tag{3.5}$$

Using the quadratic formula and taking the positive root to ensure that the transmitted ray is not refracted for normal incidence (i.e. $\delta\theta = 0$ for $\theta_1 = 0$), we find that

$$\cos\delta\theta = \frac{\frac{n_1}{n_2}\tan^2\theta_1 + \sqrt{1+\tan^2\theta_1\left(\frac{\Delta n}{n_2}\right)^2}}{1+\tan^2\theta_1}. \tag{3.6}$$

Substituting Eq. (3.6) into Eq. (3.2) and defining $\delta n \equiv \Delta n/n_2$ gives

$$\frac{\delta H}{H} = \frac{1+\delta n\tan^2\theta_1 - \sqrt{1+\tan^2\theta_1(\delta n)^2}}{(1-\delta n)\tan^2\theta_1 + \sqrt{1+\tan^2\theta_1(\delta n)^2}}. \tag{3.7}$$

As desired, Eq. (3.7) expresses the fractional deviation in pathlength in terms of the incident angle and a reduced refractive index difference δn.

While Eq. (3.7) has the benefit of being exact (in the context of geometric optics), its form is relatively complex. To develop intuition, we explore the limit where $u = \tan^2\theta_1(\delta n)^2 \ll 1$. For $\Delta n \approx 0.065$ (similar to FUS) and $n_1 \approx 1.333$ for water near room temperature at visible wavelengths, $u \leq 0.1$ for incident angles between 0° and 65°. In this limit, we approximate $\sqrt{1+u} \approx 1 + \frac{1}{2}u$ and Eq. (3.7) simplifies significantly to

$$\frac{\delta H}{H} \approx \frac{x(\delta n)}{\csc^2\theta_1 + x(\delta n)} \tag{3.8}$$

where

$$x(\delta n) = \delta n - \tfrac{1}{2}(\delta n)^2 = \frac{\Delta n}{n_2} - \frac{1}{2}\left(\frac{\Delta n}{n_2}\right)^2. \tag{3.9}$$

Eq. (3.8) helps to clarify that the excess pathlength accumulated due to refraction is small when $x(\delta n) \ll \csc^2\theta_1$. This suggests that errors incurred by neglecting refraction in the QPI analysis will be small in this limit.

Plots of the exact deviation (Eq. (3.7)) as a function of incident angle for representative values of Δn show that the fractional deviation in pathlength initially grows slowly from zero and stays well below 10% for all incident angles (**Fig. S1b**). Further, we find that the approximate deviation (Eq. (3.8)) agrees with the exact expression over this range of refractive index mismatch for nearly all incident angles ($\theta_1 < 85°$). For droplets with a contact angle of 90° or more, which is typical on passivated substrates, half of the droplet's projected surface area is illuminated at incident angles at or below 45°. In the case of FUS-like condensates with $\Delta n \approx 0.065$, the fractional pathlength deviation increases over this range from 0 at normal incidence to only 2.3% at an incident angle of 45°.

This deviation in optical pathlength is one of two primary distortions induced by diffraction. The second is a shift in the apparent position of the incident ray by an amount $H' \sin(\delta\theta) = (H + \delta H)\sin(\delta\theta)$. We note that the angular deviation $\delta\theta$ is small for the relevant range of refractive index mismatches for all but the most oblique incident angles (Eq. 3.6). Since $\sin(\delta\theta)$ vanishes as $\delta\theta$ in the limit $\delta\theta \to 0$, the refraction-induced lateral shift in ray position will also tend towards zero for most incident angles. Further, because oblique incidence occurs only as $H \to 0$ (i.e. far from the droplet center), the absolute magnitude of lateral shifts will be small for large incident angles as well.

In summary, refraction introduces deviations in the phase profile of a sessile droplet through alterations in optical pathlength as well as shifts in apparent ray position. While we

anticipate that refraction-induced distortions will degrade Δn estimates for very high index mismatches, we find that our approach recovers Δn accurately over the range typical of biomolecular condensates (**Fig. 1j**).

Supplementary Note 4: Linear approximation to refractive index of mixtures

There is an extensive literature regarding appropriate "mixing rules" for calculating the refractive index of mixtures [16–19], which in general depend non-linearly on concentration. Recognizing this, we make no claim regarding the universality of the approximation used in this paper, namely that the refractive index of a mixture is given by a linear sum of contributions from the non-solvent components. Rather, we emphasize that the refractive index of aqueous solutions of biomolecules often shows a linear concentration dependence over a wide range of concentrations [20–23].

As checks for the validity of the approximation in the current work, we demonstrate linearity explicitly for the protein BSA, the linear polymer PEG, the branched polymer dextran, and the nucleic acid polyA RNA (**Fig. 1k, Fig. S3**). As a check on the linear sum approximation for multi-component mixtures, we compared the refractive index estimated from the linear sum approximation to measurements of homogeneous PEG/dextran mixtures and found only small deviations between the two (**Fig. S4**). Further, in the case of a ternary mixture of RNA and the RNA-binding protein FUS, we show that a more rigorous non-linear analysis accounting for a FUS/RNA cross-term in the context of a quadratic model for the refractive index produces revised dense-phase concentrations that deviate by 4% or less from those obtained by the linear analysis (**Extended Data Fig. 5**, **Extended Data Fig. 6**). These data thus support the use of the linear sum approximation (Main Text **Eq. 2**) for the systems studied in this work.

Supplementary Note 5: Measuring droplet shape on flat surfaces

To characterize the shape of condensates typical of *in vitro* reconstitution experiments**,** we model them physically as sessile fluid droplets on a flat substrate (**Fig. 1c)**. In this context, droplet shape is determined at equilibrium by interfacial tension opposing gravitational settling of the denser fluid[24]. For droplets smaller than the capillary length $l_c = (\gamma/\Delta\rho g)^{1/2}$, the interfacial tension γ is sufficiently strong to suppress expansion of the interfacial area driven by gravitational effects and the resulting shape is described to an excellent approximation as a spherical cap[24] (**Fig. 1d, Fig. S5**). This approximation is valid for most reconstituted condensates, which are typically smaller than our capillary length estimate of $\gtrsim 30$ µm (**Fig. S5**). In this limit, four parameters suffice to fully characterize droplet shape: droplet radius R, the position of the droplet center (x_0, y_0), and the height of the equatorial plane above the substrate, Z_{eq} (**Fig. 1d, Methods**). Importantly, the corresponding function for the local thickness $H(x, y|R, x_0, y_0, Z_{eq})$ has a closed analytic form that we use to determine the refractive index and shape parameters of droplets from QPI images by fitting (**Fig. 1e**, **Methods, Extended Data Fig. 1**).

Supplementary Note 6: Measurement of c_{Dil}

The final parameter in Eq. (3) from the Main Text is c_{Dil}. If sufficiently large, c_{Dil} can be determined from the refractive index of the dilute phase, $n(c_{Dil})$. Since the dilute phase accounts for > 99 % of the sample volume in typical biomolecular condensate reconstitutions, it is typically possible to produce (e.g. via centrifugation) the ~ 100 μL of pure dilute phase required to measure $n(c_{Dil})$ with bulk techniques like digital refractometry. The concentration threshold for c_{Dil} measurements at 2-digits of precision with a typical digital refractometer is about 0.5 mg/ml (see also **Supplementary Note 2.2**). For $c_{Dil} < c_{thresh} \approx$ 0.5 mg/mL, standard analytic chemistry techniques (e.g. UV-Vis spectroscopy, mass spectrometry, or Western blotting) may be required to determine c_{Dil}. However, as we find c_{Cond} is typically $\gtrsim$ 100 mg/ml, the contribution of c_{Dil} to c_{Cond} is typically negligible, particularly when c_{Dil} is too low to detect by refractometery. Therefore, the contribution of c_{Dil} in Eq. (3) from the Main Text may often be neglected.

Supplementary Note 7: Conversion from Δn to concentration

Conversion from Δn to compositional differences requires knowledge of the refractive index increments, *dn/dc*, for each partitioning component. Using bulk refractometry, we determined *dn/dc* from the concentration-dependence of solution refractive index for several representative (bio)polymers (**Fig. 1k, Fig. S3**). In each case, we found excellent linearity over the entire range probed. Importantly, the measured dn/dc value for BSA is consistent with estimates from amino acid sequence[15]. This validates the use of sequence-based dn/dc estimates in the following, particularly given the impracticality of direct measurement for many recombinant proteins (**Supplementary Note 2**). With *dn/dc* estimated from protein sequence, Δn measured by QPI, and c_{Dil} measured by standard analytical methods or neglected (**Supplementary Note 6**), Eq. 3 in the Main Text enables calculation of condensed-phase protein concentrations in binary systems.

Supplementary Note 8: Temperature-dependent phase behavior

Biomolecular condensates are intrinsically temperature-dependent as thermodynamic phases, making temperature an important control parameter subject to evolutionary selection[25]. To test whether we could detect temperature-induced composition variation with QPI, we analyzed phase images of condensates containing either FUS or TAF15(RBD) acquired at temperatures between 5 and 50 ºC set with a custom temperature stage (**Methods**,[26]). After accounting for the temperature-dependence of optical constants in Eq. 1 of the Main Text (**Fig. S7**), we find that the condensed-phase protein concentration decreases significantly with increasing temperature in both cases (**Fig. 2d**). This is indicative of upper-critical solution temperatures in both cases (compare to **Fig. 1a**), as has been reported for several other[11,27], though not all[25], RNA-binding proteins. Interestingly, FUS condensates display a steeper temperature dependence than is seen for the evolutionarily-related TAF15(RBD). This suggests that, in addition to an ~ 100 mg/ml difference in internal concentration near room temperature, the physical properties of FUS condensates are relatively responsive to temperature changes while TAF15(RBD) condensates are comparatively insensitive. Finally, we note that, in contrast to the immersion objectives typical of tomographic phase imaging[9,28], the dry objective lenses used for QPI here enable fast temperature equilibration by avoiding direct coupling of their thermal mass to the temperature stage.

Supplementary Note 9: Alternative interpretations of the refractive index increase during aging

While we interpret the time-dependent increase in condensate refractive index difference Δn we observe for aging PGL-3 condensates as corresponding to an increase in dense-phase concentration c_{Cond} (**Fig. 3**), other interpretations are possible. In particular, we note that since $\Delta n \approx c_{Cond}\left(\frac{dn}{dc}\right)^{-1}$ for binary systems, a conformation-driven decrease in the refractive index increment dn/dc could also drive an increase in Δn. In this Supplementary Note, we discuss the potential contribution of changes in protein conformation to the refractive index increase and justify our interpretation that the majority of the aging-associated refractive index change comes from a concentration increase.

The refractive index increment of a protein generally depends on conformation. However, existing reports shown only a very small impact of 3D structure for proteins [15,20,29,30]. Ref. [29] found that digestion of a typical protein like BSA into small fragments with pepsin increases the refractive index of a 4-mg/ml BSA solution by 8×10^{-6}, corresponding to a dn/dc increase of only 1%. Structure exerts a larger influence for proteins of the eye lens owing to their evolution under a selective pressure for high refraction [30], but even in this case experimental dn/dc values exceed sequence-derived predictions by less than 10%. For structural rearrangements to fully account for the refractive index increase we observe during the aging timecourse of **Fig. 3**, dn/dc would need to double. The available data imply this is improbable.

In contrast, several factors favor our interpretation that the refractive index increase corresponds to a concentration increase. First, an increase in concentration is consistent with the material contraction observed previously in aging PGL-3 droplets [31]. Second, an increase in concentration is consistent with simple thermodynamic expectations for aging condensates in a rugged free-energy landscape. Whether through the gradual formation of stable secondary structures [32], clustering of stickers [33], or some other microscopic mechanism, the typical interaction energy per polymer chain (averaged over all residues) in an aging condensate increases with time. In a mean-field Flory-Huggins-like picture, this corresponds to an interaction strength that increases with time. All other things being equal, strengthening interactions widens the binodal, resulting in an increase in dense-phase concentration. Third, since the viscosity of polymer solutions increases strongly with concentration, an age-dependent concentration would help to explain previous reports of increasing PGL-3 viscosity during aging [31]. Given the weak influence of protein structure on dn/dc relative to the strong

increase we observe in refractive index during aging, the most parsimonious interpretation is that the majority of the refractive index change comes from a concentration increase.

Supplementary Note S10: Implications of protein ejection from condensates during aging

All other things being equal, the transfer of protein from the dense phase to the dilute phase we report in **Fig. 3** would result in an increase in dilute-phase protein concentration. In this Supplementary Note, we discuss the possibility that the protein mass loss we report is driven by a shift in a global system parameter (e.g. pH, ionic strength or temperature) as well as the likely role of protein adsorption to non-passivated surfaces of the imaging chamber.

Time-dependent variation of environmental parameters like pH, ionic strength or temperature is a plausible explanation for the mass ejection in principle, as such changes could shift the binodal towards an equilibrium with higher dilute-phase concentration. However, several lines of evidence suggest that this is not the dominant effect in our PGL-3 experiments. First, the samples are sealed in the flow-cell with a two-component glue and we typically see no evidence of evaporation or seal breakage even after multiple days. Second, the sample was maintained near 25 °C during the aging experiments with two Peltier elements and a feedback controller [26]. With logging at 1-second intervals, the largest deviation in the temperature trace was less than 0.02 °C. Third, our imaging conditions (one acquisition with low-power incoherent 650-nm light every 15 min with no fluorophores) are unlikely to generate significant reactive oxygen species or other free radicals associated with fluorescence imaging that might alter the composition of the sample over time [34–38]. Fourth, one expects changes to a global environmental parameter to alter both the dilute and dense binodal branches; for molecules like PGL-3 with an upper critical salt concentration, increasing ionic strength raises the (equilibrium) dilute-phase concentration while lowering the dense-phase concentration. Such an environmentally-driven binodal shift thereby accommodates the efflux of PGL-3 molecules we see into the dilute phase, but predicts the dense-phase concentration to change in the opposite direction to what we observe.

Instead, we suspect the dominant driver is adsorption of protein to the non-passivated surfaces of the imaging chamber. While the coverglass upon which the condensates sit is passivated with PEG, the chamber sides (parafilm) and top surface (sapphire) are untreated and likely somewhat sticky for protein. These surfaces are also in direct contact with the dilute phase. In the absence of condensates, adsorption to these surfaces would slowly deplete protein from solution. In the presence of condensates, however, losses from adsorption are offset by partial condensate dissolution, essentially buffering dilute-phase protein concentration. While the exact dynamics in the PGL-3 system are complicated further by the aging known to occur at long times [31], we suspect that adsorption likely drives the majority of the protein mass ejection we observe.

Supplementary Note 11: Requirement that tie-lines are straight

The goal of this section is to demonstrate that thermodynamic tie-lines in multi-component systems are necessarily linear relationships between component concentrations, and therefore are represented in phase diagrams by straight lines (when plotted on linear axes). We proceed by introducing necessary definitions, and then showing that this linear relationship follows directly from mass conservation and incompressibility.

Consider a closed system of total volume V^{tot} filled with molecules of N distinct chemical species. Let c_i, $M_{W,i}$, and $\bar{v}_i$ be the molar concentration (mole/liter), molar mass (grams/mole), and partial specific volume (liter/gram) of component i. The average system composition is uniquely specified by $\bar{\phi} = \{\bar{\phi}_1, \dots, \bar{\phi}_i, \dots, \bar{\phi}_N\}$, where $\bar{\phi}_1 = c_i M_{W,i} \bar{v}_i$ is the average volume fraction of species i.

Without loss of generality, we assume that the chemical species are not all mutually miscible at all proportions. That is, we assume that there is a contiguous region in the N-dimensional composition-space spanned by $\bar{\phi}$ in which the system demixes into two or more phases. For simplicity, we restrict ourselves here to the case where demixing results in exactly two phases. In this case, the region of the miscibility gap is bounded by an $(N-1)$-dimensional manifold called the binodal. Since this manifold is either closed or bounded by a subset of the (ϕ_i, ϕ_j)-planes defining the outer edges of composition space, the notions of "inside" and "outside" the binodal are unambiguously defined. For $\bar{\phi}$ values outside the binodal, the system exists in equilibrium as a single homogeneous phase whose composition is given by $\bar{\phi}$. For $\bar{\phi}$ inside the binodal, however, the system consists, in equilibrium, of two co-existing phases labeled I and II, the compositions of which are given by points ϕ^I and ϕ^{II} on the binodal. The collection of points $\bar{\phi}$ in the immiscible region which map to the same two points ϕ^I and ϕ^{II} on the binodal form a contiguous curve called a tie-line.

To show that there is a mathematical relationship between the points $\bar{\phi}$, ϕ^I, and ϕ^{II} on a tie-line, we start by noting that mass conservation requires that the total mass of component i in the system equal the sum of the mass of i in each of the phases

$$M_i^{tot} = M_i^I + M_i^{II}. \qquad (11.1)$$

Since the mass of component i in a volume V is given by $M_i = c_i M_{W,i} V = \phi_i V / \bar{v}_i$, Eq. (11.1) becomes

$$\bar{\phi}_i V^{tot} = \phi_i^I V^I + \phi_i^{II} V^{II}, \qquad (11.2)$$

where V^I and V^{II} are the volumes of the two phases. Eq. (11.2) represents a relationship between the amount of a single component in the entire system and the amounts in each phase.

Before we can demonstrate a relationship between these values for multiple components simultaneously, it is useful to first simplify Eq. (11.2) by removing the dependence on V^{II}. To this end, we assume that the system is incompressible, such that

$$V^{tot} = V^{I} + V^{II}. \tag{11.3}$$

With this constraint, Eq. (11.2) can be rewritten as

$$V^{I} = \frac{\phi_i^{II} - \bar{\phi}_i}{\phi_i^{II} - \phi_i^{I}} V^{tot}, \tag{11.4}$$

a statement of the well-known lever-rule [39] that gives the volume of a phase in terms of the amounts of a component in each phase as well as the system as a whole.

To obtain a relationship between compositions of two different components, we note that applications of Eq. (11.4) with components $i \neq j$ must give the same phase volume

$$\frac{\phi_i^{II} - \bar{\phi}_i}{\phi_i^{II} - \phi_i^{I}} V^{tot} = V^{I} = \frac{\phi_j^{II} - \bar{\phi}_j}{\phi_j^{II} - \phi_j^{I}} V^{tot}. \tag{11.5}$$

Eq. (11.5) can be rearranged to obtain an expression for the average volume fraction of one component in the immiscible region in terms of the average volume fraction of a second component

$$\bar{\phi}_j = \left[\frac{\phi_j^{II} - \phi_j^{I}}{\phi_i^{II} - \phi_i^{I}}\right] \bar{\phi}_i + \left[\phi_j^{II} - \left(\frac{\phi_j^{II} - \phi_j^{I}}{\phi_i^{II} - \phi_i^{I}}\right) \phi_i^{II}\right]. \tag{11.6}$$

Importantly, Eq. (11.6) provides a linear relationship between $\bar{\phi}_j$ and $\bar{\phi}_i$, written here in the form of a straight line in the (ϕ_i, ϕ_j)-plane with slope

$$m = \frac{\phi_j^{II} - \phi_j^{I}}{\phi_i^{II} - \phi_i^{I}} \tag{11.7}$$

and y-intercept

$$b = \phi_j^{II} - \left(\frac{\phi_j^{II} - \phi_j^{I}}{\phi_i^{II} - \phi_i^{I}}\right) \phi_i^{II}. \tag{11.8}$$

Note that both the slope and y-intercept depend only on the coordinates of the points on the binodal, and are thus constant for all $\bar{\phi}$ on the same tie-line. This indicates that the projection of the tie-line into the (ϕ_i, ϕ_j)-plane is linear. Since Eq. (11.6) is linear for any choice of two distinct components i and j, it follows that the tie-line must appear linear following projection into any plane spanned by a pair of distinct components. Furthermore, since the only curve for which every projection is linear is itself linear, we conclude that the tie-line must be a linear (i.e. a straight line) function embedded in the N-dimensional space spanned by ϕ, as claimed.

12 Supplementary Note: Derivation of condensate composition for multi-component systems (linear ATRI)

In this Supplementary Note, we derive **Eq. 4** of the main text for Analysis of Tie-lines and Refractive Index (ATRI) in the limit of a linear model for the refractive index of a mixture. In the first section, we explicitly consider a ternary mixture composed of one solvent and two solutes. We construct a linear system of constraint equations, invert them, and present the solution. In the second section, we repeat this procedure in the context of a quaternary system (1 solvent + 3 solutes). We provide expressions in forms that readily generalize to systems with more components, and comment on selection of tie-line projections and requirements for the matrix representing the linear system to be invertible. In the third section, we briefly present the the calculation for a mixture with an arbitrary number of components (1 solvent + N solutes). In the fourth section, we outline the procedure used to calculate the uncertainty in the condensed-phase concentrations obtained with this method, using the FUS/RNA ternary system as an explicit example. An extension of ATRI to the non-linear case of a quadratic model for the refractive index of a mixture is presented in **Supplementary Note 13**.

12.1 Ternary systems

In this section, we study the regime of two-phase coexistence of a ternary mixture at constant temperature. Specifically, we derive an explicit expression for the composition of one phase in terms of the composition of the other, the system average composition, and the refractive index increments of the solutes.

Consider a closed system of total volume V^{tot} containing three distinct chemical species, a solvent and $N = 2$ solutes, indexed by $i \in \{0, 1, 2\}$. In the context of the experiments of **Fig. 4** in the main text, these species would be an effective solvent representing the aqueous buffer, the protein FUS-GFP and polyA RNA. Similar to **Supplementary Note 11** above, let $M_{W,i}$ and $\overline{\nu}_i$ be the molar mass (grams/mole), and partial specific volume (liter/gram) of species i, but with c_i now the average mass concentration (grams/liter) of species i. The average system composition is uniquely specified by $\bar{\mathbf{c}} = \{\bar{c}_0, \bar{c}_1, \bar{c}_2\}$.

We start by assuming the existence of a two-phase coexistence region in the composition space spanned by $\bar{\mathbf{c}}$, i.e. that the three components are not mutually miscible in all proportions. Though the Gibbs Phase Rule permits the existence of a three-phase coexistence region, we restrict ourselves in the following to consider $\bar{\mathbf{c}}$ only in the two-phase region. We label the phases with Roman numerals, indexed by α. Without loss of generality, we assume that the concentration of component 1 is higher in phase II than in phase I, i.e. $c_1^I < \bar{c}_1 < c_1^{II}$. Relative to component 1, phase I thus corresponds to the dilute phase, while phase II corresponds to the condensed phase.

Given an average composition $\bar{\mathbf{c}} = \mathbf{c^B}$ in the two-phase regime and the corresponding dilute-phase composition $\mathbf{c^I} = \mathbf{c^A}$, our goal is to calculate the composition of the coexisting condensed phase, $\mathbf{c^{II}}$. To uniquely determine the concentrations of all three components at $\mathbf{c^{II}} \equiv \{c_0^{II}, c_1^{II}, c_2^{II}\}$, we require three

linearly independent equations relating these concentrations to other known values. The first equation come from assuming both phases to be incompressible, such that Eq. (7.3) holds. Specifically, incompressibility implies that the sum of component volume fractions in each phase α sum to unity,

$$\sum_i \phi_i^\alpha = \sum_i c_i^\alpha \overline{\nu}_i = 1. \tag{12.1}$$

From this relationship, the concentration of component i in phase α can be calculated immediately if the other two are known:

$$c_i^\alpha = \overline{\nu}_i^{-1}(1 - \sum_{j \neq i} c_j^\alpha \overline{\nu}_j). \tag{12.2}$$

In the following, we assume component 0 to be the (effective) solvent, and use Eq. 12.2 to determine c_0 in each phase. The problem of calculating $\mathbf{c^{II}}$ in a ternary system is thus reduced to determining c_1^{II} and c_2^{II}. This is the situation described graphically in **Fig. 4a** of the main text in the context of a phase diagram in which species 1 and 2 are both enriched in phase II. We note, however, that the approach does not require that species 2 also be enriched in phase II.

As indicated above, we require two additional constraints to proceed. Physically, these constraints come from measurements of the refractive index difference between the two co-existing phases by QPI, and the thermodynamic tie-line connecting $\mathbf{c^I}$ to $\mathbf{c^{II}}$. Approximating the refractive index difference as a linear combination of contributions from concentration differences in each non-solvent component, we can express it as

$$\Delta n = \frac{dn}{dc_1}(c_1^{II} - c_1^I) + \frac{dn}{dc_2}(c_2^{II} - c_2^I), \tag{12.3}$$

where $\frac{dn}{dc_i}$ is the refractive index increment of component i. By rearranging Eq. 12.3 slightly and replacing (c_1^{II}, c_2^{II}) with the generic point (c_1, c_2), we arrive at the form of a straight line in the (c_1, c_2)-plane:

$$\begin{aligned} c_2 &= -\left(\frac{\frac{dn}{dc_1}}{\frac{dn}{dc_2}}\right) c_1 + \left(\frac{1}{\frac{dn}{dc_2}}\left(\Delta n + \frac{dn}{dc_1} c_1^I\right) + c_2^I\right) \\ &= m_{IRL} c_1 + b_{IRL}. \end{aligned} \tag{12.4}$$

Physically, Eq. 12.4 represents a line of constant refractive index difference (isorefractive line) in the (c_1, c_2)-plane. This can be seen by noting that each point (c_1, c_2) satisfying Eq. 12.4 specifies a potential condensed-phase composition for which the refractive index difference between it and the dilute phase (c_1^I, c_2^I) is consistent with the measured value Δn.

For the final constraint, we construct the thermodynamic tie-line connecting $\mathbf{c^I}$ to $\mathbf{c^{II}}$ in the (c_1, c_2)-plane. The tie-line slope can be calculated directly from the coordinates of $\mathbf{c^I}$ and $\overline{\mathbf{c}}$ as

$$m_{TL} = \frac{\overline{c}_2 - c_2^I}{\overline{c}_1 - c_1^I}, \tag{12.5}$$

while the y-intercept is given by

$$b_{TL} = \overline{c}_2 - m_{TL}\overline{c}_1. \tag{12.6}$$

The equation for the tie-line in the (c_1, c_2)-plane may therefore be written as

$$c_2 = m_{TL} c_1 + (\bar{c}_2 - m_{TL} \bar{c}_1) \,. \tag{12.7}$$

Since the point (c_1^{II}, c_2^{II}) must lie on the tie-line by definition, our final constraint is thus

$$c_2^{II} = m_{TL} c_1^{II} + (\bar{c}_2 - m_{TL} \bar{c}_1) \,. \tag{12.8}$$

Taken together, equations 12.3 and 12.8 suffice to fully specify a system of linear equations in the unknowns (c_1^{II}, c_2^{II}). We now cast this in matrix form as

$$\begin{bmatrix} \frac{dn}{dc_1} & \frac{dn}{dc_2} \\ m_{TL} & -1 \end{bmatrix} \begin{bmatrix} c_1^{II} \\ c_2^{II} \end{bmatrix} = \begin{bmatrix} \Delta n + \frac{dn}{dc_1} c_1^I + \frac{dn}{dc_2} c_2^I \\ m_{TL} \bar{c}_1 - \bar{c}_2 \end{bmatrix} \tag{12.9}$$

$$M \mathbf{c^{II}} = \mathbf{x}. \tag{12.10}$$

With the identification $(c_1^{II}, c_2^{II}) \rightarrow (p^{II}, r^{II})$, Eq. 12.9 is equivalent to that presented in **Fig. 4b** in the main text. To assess invertability of the matrix M, we next calculate its determinant,

$$det(M) = -\frac{dn}{dc_1} - m_{TL} \frac{dn}{dc_2}. \tag{12.11}$$

Noting that the refractive index increments for most biomolecules in water are positive constants, the matrix M is singular (non-invertible) only if $m_{TL} = -\frac{dn}{dc_1} / \frac{dn}{dc_2} = m_{IRL}$. The indeterminacy stems from the fact that, in this special case, the isorefractive line and the tie-line would be coincident; Equations 12.3 and 12.8 would describe the same line and thus not provide linearly independent information. Such a special case would correspond strictly to segregative phase separation (species 1 and 2 are enriched in opposite phases), and then only in regions of the phase diagram that happen to have the misfortune of containing tie-lines with the same slope as the isorefractive line. In practice, we expect this situation to arise only rarely. In cases where matrix singularity is an issue, a change in system temperature may be sufficient to shift $det(M)$ away from the singularity by altering the tie-line slopes relative to the isorefractive line.

For the typical case of non-singular M, the system inverse is given by

$$M^{-1} = \frac{1}{det(M)} \begin{bmatrix} -1 & -\frac{dn}{dc_2} \\ -m_{TL} & \frac{dn}{dc_1} \end{bmatrix}. \tag{12.12}$$

With this, the condensed-phase composition is given by

$$\mathbf{c^{II}} = M^{-1} \mathbf{x}. \tag{12.13}$$

After some algebra, and noting that

$$c_2^I - \bar{c}_2 + \bar{c}_1 m_{TL} = c_1^I m_{TL} \tag{12.14}$$

$$c_1^I - \bar{c}_1 + \frac{\bar{c}_2}{m_{TL}} = \frac{c_2^I}{m_{TL}}, \tag{12.15}$$

Eq. 12.13 simplifies to

$$\begin{bmatrix} c_1^{II} \\ c_2^{II} \end{bmatrix} = \begin{bmatrix} \frac{\Delta n}{-det(M)} + c_1^I \\ \frac{\Delta n m_{TL}}{-det(M)} + c_2^I \end{bmatrix}. \tag{12.16}$$

Equation 12.16 was used to compute the condensed-phase concentrations of FUS-GFP and polyA RNA reported in **Fig. 4**. With c_1^{II} and c_2^{II} determined by Eq. 12.17, the solvent concentration c_0^{II} can be calculated from Eq. 12.2. The condensed-phase composition is thus fully specified.

We note that, from comparison to Eq. 12.12, the first column of M^{-1} appears on the right-hand side of Eq. 12.16. We can thus rewrite Eq. 12.16 in for the individual components c_i^{II} as

$$c_i^{II} = \Delta n M_{i1}^{-1} + c_i^I, \tag{12.17}$$

where M_{i1}^{-1} is the ith matrix element of the first column of M^{-1}. Equation 12.17 is identical to Eq. 4 given in the main text, and, as we'll see below, is the form of the general solution for the condensed-phase composition of the N solutes in an $N+1$-component mixture.

12.2 Quaternary systems

In a quaternary system (4 species), we have 1 solvent and $N = 3$ solutes indexed by $i \in \{0, 1, 2, 3\}$. As for the ternary system, we restrict ourselves to the region of the space spanned by $\bar{\mathbf{c}}$ corresponding to two-phase coexistence. We also assume, without loss of generality, that species 1 is enriched in phase II. We'll again use Eq. 12.2 to determine c_0^{II}, so we need three constraints to determine the condensed-phase concentrations of the other three components. The first constraint is the quaternary analog of Eq. 12.3 for the refractive index difference between the coexisting phases,

$$\Delta n = \frac{dn}{dc_1}(c_1^{II} - c_1^I) + \frac{dn}{dc_2}(c_2^{II} - c_2^I) + \frac{dn}{dc_3}(c_3^{II} - c_3^I), \tag{12.18}$$

which now corresponds to an isorefractive plane in the space spanned by $\langle c_1, c_2, c_3 \rangle$.

The other two constraints come from projections of the thermodynamic tie-line into 2-dimensional subspaces. In a quaternary system with $N = 3$ solutes, there are $\binom{N}{2} = 3$ such projections possible: the (c_1, c_2)-plane, the (c_1, c_3)-plane, and the (c_2, c_3)-plane. However, only $N - 1 = 2$ of these are linearly independent. We could therefore choose any two. We select here the projections into the (c_1, c_2)-plane and the (c_1, c_3)-plane, as the assumption that component 1 is enriched in phase II guarantees that the slopes of the projections are finite.

Following the procedure for ternary systems above, the slope for the tie-line projected into the (c_i, c_j)-plane is

$$m_{ij} = \frac{\bar{c}_j - c_j^I}{\bar{c}_i - c_i^I}. \tag{12.19}$$

Along with the y-intercept

$$b_{ij} = \bar{c}_j - m_{ij}\bar{c}_i, \tag{12.20}$$

the equation for the projection of the thermodynamic tie-line into the (c_i, c_j)-plane is given by

$$c_j = m_{ij} c_i + b_{ij}. \tag{12.21}$$

We next combine the constraints from the isorefractive plane (Eq. 12.18) and the tie-line projections into the (c_1, c_2)- and (c_1, c_3)-planes into a matrix equation of the form $M\mathbf{c^{II}} = \mathbf{x}$ as

$$\begin{bmatrix} \frac{dn}{dc_1} & \frac{dn}{dc_2} & \frac{dn}{dc_3} \\ m_{12} & -1 & 0 \\ m_{13} & 0 & -1 \end{bmatrix} \begin{bmatrix} c_1^{II} \\ c_2^{II} \\ c_3^{II} \end{bmatrix} = \begin{bmatrix} \Delta n + \sum\limits_{i \neq 0} \frac{dn}{dc_i} c_i^I \\ -b_{12} \\ -b_{13} \end{bmatrix}. \tag{12.22}$$

To assess whether the system can be solved, we again calculate the determinant of M, which is given by

$$det(M) = \frac{dn}{dc_1} + \frac{dn}{dc_2} m_{12} + \frac{dn}{dc_3} m_{13} = \frac{dn}{dc_1} + \sum_{i>1} \frac{dn}{dc_i} m_{1i}. \tag{12.23}$$

As always, $det(M)$ must be finite and non-zero for M to be invertible. Setting Eq. 12.23 equal to zero yields a condition on m_{13} for which M is singular and non-invertible, namely

$$m_{13} = -\left(\frac{\frac{dn}{dc_1}}{\frac{dn}{dc_3}} + \frac{\frac{dn}{dc_2}}{\frac{dn}{dc_3}} m_{12} \right). \tag{12.24}$$

Similar to the ternary case, this condition corresponds to the tie-line lying in the isorefractive plane defined by Eq. 12.18. Since the m_{ij} are generally independent of each other and also physically independent of the refractive index increments, there is no physical reason to expect this to occur often. Therefore, so long as the tie-line slopes do not by chance happen to satisfy the relationship given in Eq. 12.24, M is invertible.

We briefly mention a few specific and potentially common cases. First, we note that if the m_{ij} are both positive, $det(M)$ is positive and the system is always invertible. This corresponds to components 2 and 3 also being enriched in phase II. Second, if $m_{ij} = 0$ for one of the components j, then the concentration of that component in each phase is given by $c_j^{II} = c_j^I = \bar{c}_j$ (i.e. its partition coefficient is 1) and the situation simplifies to that of the ternary case discussed above. Third, if $m_{ij} = 0$ for both components 2 and 3, then the situation reduces to an effective binary system and can be solved from Eq. 12.18 alone.

Assuming the tie-line is such that M is non-singular, its inverse is given by

$$M^{-1} = \frac{1}{det(M)} \begin{bmatrix} 1 & \frac{dn}{dc_2} & \frac{dn}{dc_3} \\ m_{12} & -\frac{dn}{dc_1} - \frac{dn}{dc_3} m_{13} & \frac{dn}{dc_3} m_{12} \\ m_{13} & \frac{dc}{dc_2} m_{13} & -\frac{dn}{dc_1} - \frac{dn}{dc_2} m_{12} \end{bmatrix}. \tag{12.25}$$

The condensed-phase composition can thus be computed from $\mathbf{c^{II}} = M^{-1}\mathbf{x}$. After some algebra, this simplifies to

$$\begin{bmatrix} c_1^{II} \\ c_2^{II} \\ c_3^{II} \end{bmatrix} = \begin{bmatrix} \frac{\Delta n}{det(M)} + c_1^I \\ \frac{\Delta n m_{12}}{det(M)} + c_2^I \\ \frac{\Delta n m_{13}}{det(M)} + c_3^I \end{bmatrix}. \tag{12.26}$$

As in the Ternary case, comparison of Eq. 12.26 to Eq. 12.25 shows that the first column of M^{-1} again shows up in the solution. We thus find that

$$c_i^{II} = \Delta n M_{i1}^{-1} + c_i^{I} \tag{12.27}$$

for Quaternary systems as well. Physically, the matrix elements of the first column of M^{-1} denote the contribution of each component to the refractive index difference between the two phases.

12.3 General solution for (N+1)-component systems

The procedure for a solution with 1 solvent and N solute species follows directly from the results for the quaternary mixtures of the previous section. The refractive index difference between phases

$$\Delta n = \sum_{i \neq 0} \frac{dn}{dc_i} \left(c_i^{II} - c_i^{II} \right) \tag{12.28}$$

represents a hyperplane in the N-dimensional space spanned by the solute concentrations. Generating tie-line constraints from projections of the thermodynamic tie-line into the (c_1, c_j)-plane for all $N-1$ choices of j, the constraints can be written together in a matrix expression of the form $M\mathbf{c^{II}} = \mathbf{x}$ as

$$\begin{bmatrix} \frac{dn}{dc_1} & \frac{dn}{dc_2} & \frac{dn}{dc_3} & \dots & \frac{dn}{dc_i} & \dots & \frac{dn}{dc_N} \\ m_{12} & -1 & 0 & \dots & 0 & \dots & 0 \\ m_{13} & 0 & -1 & \dots & 0 & \dots & 0 \\ \vdots & \vdots & \vdots & \ddots & \vdots & \vdots & \vdots \\ m_{1i} & 0 & 0 & \dots & -1 & \dots & 0 \\ \vdots & \vdots & \vdots & \vdots & \vdots & \ddots & \vdots \\ m_{1N} & 0 & 0 & \dots & 0 & \dots & -1 \end{bmatrix} \begin{bmatrix} c_1^{II} \\ c_2^{II} \\ c_3^{II} \\ \vdots \\ c_i^{II} \\ \vdots \\ c_N^{II} \end{bmatrix} = \begin{bmatrix} \Delta n + \sum\limits_{i \neq 0} \frac{dn}{dc_i} c_i^{I} \\ -b_{12} \\ -b_{13} \\ \vdots \\ -b_{1i} \\ \vdots \\ -b_{1N} \end{bmatrix}. \tag{12.29}$$

The determinant of M is given again by

$$det(M) = \frac{dn}{dc_1} + \sum_{i>1} \frac{dn}{dc_i} m_{1i}, \tag{12.30}$$

and is zero if and only if the thermodynamic tie-line lies in the isorefractive hyperplane. As the number of solutes N increases, the likelihood of this happening by chance becomes progressively smaller.

The inverse of M in the general case is given by

$$M^{-1} = \frac{1}{det(M)} \times$$
$$\begin{bmatrix} 1 & \frac{dn}{dc_2} & \frac{dn}{dc_3} & \cdots & \frac{dn}{dc_i} & \cdots & \frac{dn}{dc_N} \\ m_{12} & -\frac{dn}{dc_1} - \sum\limits_{1<k\neq 2} \frac{dn}{dc_k} m_{1k} & \frac{dn}{dc_3} m_{12} & \cdots & \frac{dn}{dc_i} m_{12} & \cdots & \frac{dn}{dc_N} m_{12} \\ m_{13} & \frac{dn}{dc_2} m_{13} & -\frac{dn}{dc_1} - \sum\limits_{1<k\neq 3} \frac{dn}{dc_k} m_{1k} & \cdots & \frac{dn}{dc_i} m_{13} & \cdots & \frac{dn}{dc_N} m_{13} \\ \vdots & \vdots & \vdots & \ddots & \vdots & \vdots & \vdots \\ m_{1i} & \frac{dn}{dc_2} m_{1i} & \frac{dn}{dc_3} m_{1i} & \cdots & -\frac{dn}{dc_1} - \sum\limits_{1<k\neq i} \frac{dn}{dc_k} m_{1k} & \cdots & \frac{dn}{dc_N} m_{1i} \\ \vdots & \vdots & \vdots & \vdots & \vdots & \ddots & \vdots \\ m_{1N} & \frac{dn}{dc_2} m_{1N} & \frac{dn}{dc_3} m_{1N} & \cdots & \frac{dn}{dc_i} m_{1N} & \cdots & -\frac{dn}{dc_1} - \sum\limits_{1<k\neq N} \frac{dn}{dc_k} m_{1k} \end{bmatrix} . \quad (12.31)$$

Using this expression for M^{-1} in $\mathbf{c^{II}} = M^{-1}\mathbf{x}$, the condensed-phase composition for the general case simplifies (after much algebra) to

$$\begin{bmatrix} c_1^{II} \\ c_2^{II} \\ c_3^{II} \\ \vdots \\ c_i^{II} \\ \vdots \\ c_N^{II} \end{bmatrix} = \begin{bmatrix} \frac{\Delta n}{det(M)} + c_1^I \\ \frac{\Delta n m_{12}}{det(M)} + c_2^I \\ \frac{\Delta n m_{13}}{det(M)} + c_3^I \\ \vdots \\ \frac{\Delta n m_{1i}}{det(M)} + c_i^I \\ \vdots \\ \frac{\Delta n m_{1N}}{det(M)} + c_N^I \end{bmatrix}, \quad (12.32)$$

or, more compactly, as the now familiar expression

$$c_i^{II} = \Delta n M_{i1}^{-1} + c_i^I. \quad (12.33)$$

As the number of solute components N is arbitrary throughout this section, Eq. 12.33 may be used to calculate the species concentrations in phase II for mixtures with an arbitrary number of components, as claimed in the main text.

12.4 Error propagation with Jacobians

In this section we document how we calculated the uncertainty on the condensed-phase concentrations we report for multi-component systems, using the ternary FUS/RNA system in **Fig. 4** as an explicit example. The case of the 5-solute system in **Fig. 5** is handled analogously, with two exceptions. First, the uncertainty in the dilute-phase RNA concentration is taken to be equal to the standard deviation of the concentration distribution determined by Monte Carlo simulation **Extended Data Fig. 8**. Second, cross-correlations between the dilute-phase RNA concentration and other dilute-phase concentrations are estimated from the synthetic distributions determined from Monte Carlo simulations **Extended Data Fig. 9**.

For multi-component systems, the condensed-phase concentrations are calculated from Eq. 12.33. For each component i, Eq. 12.33 prescribes a function of seven variables $f_i\left(\Delta n, \frac{dn}{dc_1}, \frac{dn}{dc_2}, \overline{c}_1, \overline{c}_2, c_1^I, c_2^I,\right)$. Measurement or estimation of each variable x comes with an associated uncertainty σ_x. We estimate the variance of f_i as

$$(\delta f_i)^2 = J_i \Sigma^x J_i^T, \tag{12.34}$$

where J_i is the Jacobian of f_i. J_i is a row vector given by

$$J_i = \begin{bmatrix} \frac{\partial f_i}{\partial \Delta n} & \frac{\partial f_i}{\partial\left(\frac{dn}{dc_1}\right)} & \frac{\partial f_i}{\partial\left(\frac{dn}{dc_2}\right)} & \frac{\partial f_i}{\partial \overline{c}_1} & \frac{\partial f_i}{\partial \overline{c}_2} & \frac{\partial f_i}{\partial c_1^I} & \frac{\partial f_i}{\partial c_2^I} \end{bmatrix}, \tag{12.35}$$

and J^T is its transpose. Σ^x is a square matrix containing the cross-correlations ρ_{jk} between the uncertainties of the variables x_j and x_k weighted by the product of their respective uncertainties $\sigma_j \sigma_k$. The matrix elements of Σ^x are thus given by

$$\Sigma^x_{jk} = \rho_{jk}\sigma_j\sigma_k. \tag{12.36}$$

Final error bars on f_i would represent the standard deviation δf_i.

Since each variable x_j was determined independently for the FUS/RNA measurements in **Fig. 4**, we used a diagonal cross-correlation matrix where $\rho_{jj} = 1$ and $\rho_{jk} = 0$ for $j \neq k$. We next list how the uncertainties σ_x in each variable x were determined. For $\sigma_{\Delta n}$, we used the standard deviation of the Δn measurements from the corresponding condition. For σ_{dn/dc_2}, we used the 95 %-confidence interval from the linear fit in **Fig. S3**. Since $\frac{dn}{dc_1}$ was estimated from sequence rather than measured directly, we estimated σ_{dn/dc_1} as $\left(\frac{dn/dc_1}{dn/dc_2}\right)\sigma_{dn/dc_2}$. $\sigma_{\overline{c}_1}$ and $\sigma_{\overline{c}_1}$ represent the standard deviations of repeat spectrophotometric measurements of the stock concentrations, rescaled by the ratio $\overline{c}_i/c_i^{stock}$. Finally, $\sigma_{c_1^I}$ and $\sigma_{c_1^I}$ are proportional to standard deviations from repeat measurements of the dilute-phase UV-Vis spectra.

For calculating the variance of the condensed-phase protein concentration $\left(\delta c_1^{II}\right)^2$ from Eq. 12.34, we used

$$\begin{aligned} f_1 &= \frac{\Delta n}{-det(M)} + c_1^I \\ &= \frac{\Delta n}{\frac{dn}{dc_1} + \frac{dn}{dc_2}\frac{\overline{c}_2 - c_2^I}{\overline{c}_1 - c_1^I}} + c_1^I, \end{aligned} \tag{12.37}$$

for which the Jacobian reads

$$J_1 = -\frac{\Delta n}{(det(M))^2}\begin{bmatrix} \frac{det(M)}{\Delta n} & 1 & m_{TL} & \frac{m_{TL}\frac{dn}{dc_2}}{\left(\overline{c}_1 - c_1^I\right)} & -\frac{\frac{dn}{dc_2}}{\left(\overline{c}_1 - c_1^I\right)} & \frac{m_{TL}\frac{dn}{dc_2}}{\left(\overline{c}_1 - c_1^I\right)} - 1 & -\frac{\frac{dn}{dc_2}}{\left(\overline{c}_1 - c_1^I\right)} \end{bmatrix}, \tag{12.38}$$

with

$$det(M) = -\frac{dn}{dc_1} - \frac{dn}{dc_2}\frac{\overline{c}_2 - c_2^I}{\overline{c}_1 - c_1^I}, \tag{12.39}$$

and

$$m_{TL} = \frac{\overline{c}_2 - c_2^I}{\overline{c}_1 - c_1^I}. \tag{12.40}$$

For calculating the variance of the condensed-phase RNA concentration $\left(\delta c_2^{II}\right)^2$ from Eq. 12.34, we used

$$f_2 = \frac{\Delta n m_{TL}}{-det(M)} + c_2^I = \frac{\Delta n \left(\frac{\bar{c}_2 - c_2^I}{\bar{c}_1 - c_1^I}\right)}{\frac{dn}{dc_1} + \frac{dn}{dc_2}\frac{\bar{c}_2 - c_2^I}{\bar{c}_1 - c_1^I}} + c_2^I = \frac{\Delta n}{\frac{dn}{dc_1}\left(\frac{\bar{c}_1 - c_1^I}{\bar{c}_2 - c_2^I}\right) + \frac{dn}{dc_2}} + c_2^I = \frac{\Delta n}{\Omega} + c_2^I, \tag{12.41}$$

with

$$\Omega \equiv \frac{dn}{dc_1}\left(\frac{\bar{c}_1 - c_1^I}{\bar{c}_2 - c_2^I}\right) + \frac{dn}{dc_2}. \tag{12.42}$$

In this case, the Jacobian reads

$$J_2 = \frac{\Delta n}{\Omega^2}\begin{bmatrix} \frac{\Omega}{\Delta n} & -\frac{1}{m_{TL}} & -1 & -\frac{\frac{dn}{dc_1}}{\left(\bar{c}_2 - c_2^I\right)} & \frac{\frac{dn}{dc_1}}{m_{TL}\left(\bar{c}_2 - c_2^I\right)} & \frac{\frac{dn}{dc_1}}{\left(\bar{c}_2 - c_2^I\right)} & 1 - \frac{\frac{dn}{dc_1}}{m_{TL}\left(\bar{c}_2 - c_2^I\right)} \end{bmatrix}. \tag{12.43}$$

The final error bars on the condensed-phase protein concentrations c_1^{II} in **Fig. 4g** represent the standard deviation δc_1^{II} calculated according to

$$\delta c_1^{II} = \sqrt{J_1 \Sigma^x J_1^T}. \tag{12.44}$$

Similarly, the final error bars on the condensed-phase RNA concentrations c_2^{II} in **Fig. 4g** represent the standard deviation δc_2^{II} calculated according to

$$\delta c_2^{II} = \sqrt{J_2 \Sigma^x J_2^T}. \tag{12.45}$$

Supplementary Note 13: Condensate composition for multi-component systems with a quadratic model for refractive index (non-linear ATRI)

To calculate the composition of multi-component condensates formed in an effective ternary mixture in the context of a quadratic model for the refractive index, we briefly extend the linear ATRI theory presented in **Supplementary Note 12** to this non-linear case. This extension was used to generate the results presented in **Extended Data Fig. 5** and **Extended Data Fig. 6**.

We consider an effective ternary mixture ($N = 2$ solutes with an effective solvent) prepared at average concentrations $(\bar{c}_1, \bar{c}_2)$ in the two-phase region, such that the system is composed of two coexisting phases in equilibrium. These phases are characterized by compositions (c_1^I, c_2^I) and (c_1^{II}, c_2^{II}). Without loss of generality, we assume that component 1 is enriched in phase II and depleted in phase I, such that $c_1^I < \bar{c}_1 < c_1^{II}$. As before, the system is situated on a tie-line given by the linear relationship

$$c_2 = m_{TL}c_1 + b_{TL}, \tag{13.1}$$

where

$$m_{TL} = \frac{\bar{c}_2 - c_2^I}{\bar{c}_1 - c_1^I} \tag{13.2}$$

and

$$b_{TL} = \bar{c}_2 - m_{TL}\bar{c}_1. \tag{13.3}$$

In the quadratic approximation, the refractive index of phase α is given as

$$n^\alpha = n_{solvent} + c_1^\alpha \frac{dn}{dc_1} + c_2^\alpha \frac{dn}{dc_2} + c_1^\alpha c_2^\alpha \frac{d^2n}{dc_1 dc_2}. \tag{13.4}$$

The refractive index difference between phases measurable by QPI, $\Delta n = n^{II} - n^I$, is then

$$\Delta n = c_1^{II} \frac{dn}{dc_1} + c_2^{II} \frac{dn}{dc_2} + c_1^{II} c_2^{II} \frac{d^2n}{dc_1 dc_2} - c_1^I \frac{dn}{dc_1} - c_2^I \frac{dn}{dc_2} - c_1^I c_2^I \frac{d^2n}{dc_1 dc_2}. \tag{13.5}$$

Upon rearranging Eq. (13.5) to solve for c_2^{II}, we obtain a compact expression for the isorefractive curve in the (c_1, c_2)-plane

$$c_2^{II} = \frac{c_2^{II,max} - r c_1^{II}}{1 + \tilde{v}_{12} c_1^{II}}, \tag{13.6}$$

where

$$c_2^{II,max} \equiv \Delta n + c_1^I \frac{dn}{dc_1} + c_2^I \frac{dn}{dc_2} + c_1^I c_2^I \frac{d^2n}{dc_1 dc_2} \tag{13.7}$$

is the maximum value of c_2^{II} consistent with Δn (attained in the case where $c_1^{II} = 0$),

$$r \equiv \frac{dn}{dc_1} / \frac{dn}{dc_2} = -m_{IRL} \tag{13.8}$$

characterizes the slope of the isorefractive line from the linear case (but defined with opposite sign), and

$$\tilde{v}_{12} \equiv \frac{d^2n}{dc_1dc_2} \Big/ \frac{dn}{dc_2} \tag{13.9}$$

is a reduced refractive cross-term coefficient that controls the deviation of the isorefractive curve from the simple line obtained in the linear case.

Isorefractive curves are shown in **Extended Data Fig. 6a** for a range of $\tilde{v}_{12}$ values. For $\tilde{v}_{12} > 0$, the isorefractive curve sits below the isorefractive line from the linear case. This indicates that a linear refractive index model results in an overestimate of condensed-phase concentrations (**Extended Data Fig. 6b**) if $\tilde{v}_{12} > 0$. For $\tilde{v}_{12} < 0$, the isorefractive curve lies above the isorefractive line from the linear case. Correspondingly, use of a linear refractive index model will underestimate condensed-phase concentrations if $\tilde{v}_{12} < 0$. In both cases, the magnitude of the refractive cross-term coefficient relative to $\frac{dn}{dc_2}$ controls the severity of the over- or under-estimation. For the FUS/RNA mixtures studied in this work, we find $\tilde{v}_{12} \cong 7.4 \times 10^{-4}$ is positive and small (**Extended Data Fig. 5**), such that accounting for it yields only a minor downward adjustment of condensed-phase concentrations obtained with a linear refractive index model (**Extended Data Fig. 5e,f**).

Supplementary Note 14: Calculation of excess extinction in multi-component mixtures

This supplementary note includes three sections. In the first section, we derive a relationship between the excess optical extinction and a cross-term in a quadratic model of the refractive index of a mixture. In the second section, we discuss how buffer components may be accounted for in the analysis. In the third section, we detail the calculation of the FUS/RNA cross-term coefficient.

Supplementary Note 14.1 Definitions and theory for cross-term analysis

In this section, we present the theoretical basis supporting our quantification of a refractive index cross-term at λ = 650 nm from measurements of cross-terms in the absorption spectra at shorter wavelengths. Briefly, the theory has four conceptually-distinct steps. In Step 1, we relate the optical absorption of a sample to the imaginary component of its complex-valued refractive index. In Step 2, we define a physical response function in terms of excess quantities, and calculate the real component from the imaginary one via a Kramers-Kronig relation [40]. In Step 3, we construct a quadratic model for the optical response of a mixture in terms of the concentrations c_i of its solute species. In Step 4, we use the real component of the excess response function to quantify the refractive index cross-term $\frac{d^2n}{dc_idc_j}c_ic_j$, where $\frac{d^2n}{dc_idc_j}$ is the unknown cross-term coefficient, focusing on the case of a ternary FUS/RNA mixture.

Step 1

Let $A(\lambda)$ denote the wavelength-dependent absorbance measured by UV-Vis spectroscopy for a sample of thickness L. This absorbance is directly related to the sample's optical extinction κ, which is defined as the imaginary-component of a sample's complex-valued refractive index: $n^* = n + i\kappa$. The extinction can be expressed in terms of the absorbance as

$$\kappa(\lambda) = \lambda A(\lambda)/\left(4\pi L\log_{10}(e)\right). \quad (14.1)$$

This may be converted to angular frequency by substituting $\lambda = \frac{c_0}{v} = \frac{c_0}{2\pi\omega}$, where c_0 is the speed of light in vacuum, yielding

$$\kappa(\omega) = c_0A(\omega)/\left(2\log_{10}(e)L\omega\right). \quad (14.2)$$

Step 2

In general, the optical responses of mixtures may deviate from the predictions of simple mixing rules expressed as a linear combination (**Supplementary Note 4**). To characterize this

deviation, we introduce certain “excess” quantities as the difference between the measured value and that predicted by a linear model for the concentration-dependence of the solutes. Specifically, we define the frequency-dependent “excess extinction” as

$$\kappa_{excess}(\omega) = \kappa_{mix}(\omega) - \kappa_{Linear}(\omega), \quad (14.3)$$

where

$$\kappa_{Linear}(\omega) \equiv \kappa_{solvent}(\omega) + \sum_i c_i \varepsilon_i(\omega), \quad (14.4)$$

with the concentration and extinction coefficients of species i given by c_i and ε_i, respectively. Note that this extinction coefficient is related to that from the linear model of absorbance known as Beer's law [17] by a factor of $c_0/(2\log_{10}(e)\omega)$. Similarly, we define the excess refractive index as

$$n_{excess}(\omega) = n_{mix}(\omega) - n_{Linear}(\omega), \quad (14.5)$$

with

$$n_{Linear}(\omega) \equiv n_{solvent}(\omega) + \sum_i c_i \frac{dn}{dc_i}(\omega). \quad (14.6)$$

Together, a mixture's excess complex-valued refractive index is the response function given by

$$n^*_{excess}(\omega) = n_{excess}(\omega) + i\kappa_{excess}(\omega). \quad (14.7)$$

Since $n^*_{excess}(\omega)$ as defined above is a physical response function of the form $\chi^*(\omega) = \chi'(\omega) + i\chi''(\omega)$ [40], we can calculate $n_{excess}(\omega) = \chi'(\omega)$ from $\kappa_{excess}(\omega) = \chi''(\omega)$ using the Kramers-Kronig relation [40]

$$\chi'(\omega) = \frac{2}{\pi}\mathcal{P}\int_0^{+\infty} \frac{\omega'\chi''(\omega')}{\omega'^2 - \omega^2} d\omega', \quad (14.8)$$

where $\mathcal{P}$ denotes the Cauchy principal value of the integral. Note that Eq. (14.8) is modified from the typical formulation by multiplying the integrand by $(\omega' + \omega)/(\omega' + \omega)$ and using the fact that $\omega'\chi''(\omega')$ is even while $\omega\chi''(\omega')$ is odd to adjust the integration limits to the positive half-line. The deviation of the refractive index relative to the prediction of a linear mixture rule is thus given by $n_{excess}(\omega)$ obtained from absorbance measurements via Eqs. (14.2), (14.3), and (14.8) above.

Step 3

Deviations of the refractive index from linearity can also be described in terms of an explicitly non-linear mixing model. One generic approach is to express the refractive index as a power series in the solute concentrations. A convenient aspect of a power-series expansion is that it reduces to a linear model when the coefficients are set to zero for all higher-than-first-order

terms. The simplest non-linear power-series expansion is a quadratic model. In its most generic form, the quadratic model for the refractive index of a mixture is given by

$$n_{Quadratic}(\omega) \equiv n_{solvent}(\omega) + \sum_i c_i \frac{dn}{dc_i}(\omega) + \sum_{i,j\geq i} c_i c_j \frac{d^2n}{dc_i dc_j}(\omega). \quad (14.9)$$

For a mixture with N solutes, this general expression contains $N(N+1)/2$ unique 2nd-order terms, of which N are (diagonal) "self-terms" and $N(N-1)/2$ are cross-terms.

Step 4

For concreteness, we now consider FUS/RNA mixtures. Treated as effective ternary mixture containing $N = 2$ solutes and 1 effective solvent, there are $2(2+1)/2 = 3$ 2nd-order terms: two self-terms and one cross-term. The linearity seen in solutions of protein alone ([20], **Fig. 1k**) and RNA alone (**Fig. S3**) suggests that the quadratic self-terms for protein and RNA are very close to zero and may therefore be neglected. If the two self-terms are negligible, then the only remaining source for an appreciable non-linearity in a quadratic model of a ternary mixture is the one cross-term. For this reason, the focus of our non-linear modeling for FUS/RNA mixtures in this work is on the cross-term $c_{FUS}c_{RNA}\frac{d^2n}{dc_{FUS}dc_{RNA}}$.

With the self-terms set to zero, a quadratic model for the refractive index of a FUS/RNA ternary mixture then takes the form

$$n_{Ternary}(\omega) = n_{solvent}(\omega) + c_1\frac{dn}{dc_1}(\omega) + c_2\frac{dn}{dc_2}(\omega) + c_1c_2\frac{d^2n}{dc_1dc_2}(\omega), \quad (14.10)$$

where the indices 1 and 2 correspond to FUS and RNA, respectively, and $n_{solvent}$ is understood as the refractive index of the effective solvent. In the effective ternary approximation, the excess refractive index for the mixture then becomes

$$n_{excess}(\omega) \cong n_{Ternary}(\omega) - n_{Linear}(\omega) = c_1c_2\frac{d^2n}{dc_1dc_2}(\omega), \quad (14.11)$$

or equivalently as a function of wavelength

$$n_{excess}(\lambda) \cong c_1c_2\frac{d^2n}{dc_1dc_2}(\lambda). \quad (14.12)$$

In this manner, the cross-term coefficient $\frac{d^2n}{dc_{FUS}dc_{RNA}}$ at the imaging wavelength $\lambda = 650$ nm may be determined by transformation and integration of the excess absorbance measured by UV-Vis spectroscopy (**Extended Data Fig. 4, Extended Data Fig. 5**).

Supplementary Note 14.2: Explicit incorporation of buffer components

Here we demonstrate that a quadratic model for FUS/RNA mixtures that accounts explicitly for individual buffer components (i.e. Tris, KCl, DTT, and glycerol) can also be written in the form of Eq. (14.10). This follows directly by regrouping the terms in Eq. (14.9) into those proportional to c_{FUS} but not c_{RNA}, those proportional to c_{RNA} but not to c_{FUS}, those proportional to neither, and the one cross-term proportional to both. To show this, we start by arranging the coefficients of the 21 2nd-order terms of the $N = 6$-solute system in a square matrix whose matrix elements ν_{ij} are defined as

$$\nu_{ij} \equiv \frac{d^2n}{dc_i dc_j} = \begin{bmatrix} \nu_{11} & \nu_{12} & \nu_{13} & \nu_{14} & \nu_{15} & \nu_{16} \\ 0 & \nu_{22} & \nu_{23} & \nu_{24} & \nu_{25} & \nu_{26} \\ 0 & 0 & \nu_{33} & \nu_{34} & \nu_{35} & \nu_{36} \\ 0 & 0 & 0 & \nu_{44} & \nu_{45} & \nu_{46} \\ 0 & 0 & 0 & 0 & \nu_{55} & \nu_{56} \\ 0 & 0 & 0 & 0 & 0 & \nu_{66} \end{bmatrix}. \tag{14.13}$$

For concreteness, let the solutes FUS, RNA, Tris, KCl, DTT, and glycerol be indexed in order by 1-6. As before, linearity of FUS solutions and RNA solutions allows the self-terms containing ν_{11} and ν_{22} to be set to zero. The ν_{12} term is proportional to both c_{FUS} and c_{RNA}, and is identified as the FUS/RNA cross-term. The remaining terms in the first row are all proportional to c_{FUS} but not to c_{RNA}, and may be combined with the linear $c_{FUS}\frac{dn}{dc_{FUS}}$ term to represent a buffer-specific refractive index increment for FUS as

$$\frac{\widehat{dn}}{dc_{FUS}} \equiv \frac{dn}{dc_1} + \sum_{j=3}^{N=6} c_j \nu_{1j}, \tag{14.14}$$

Similarly, the remaining terms in the second row are all proportional to c_{RNA} but not to c_{FUS}, and may be combined with the linear $c_{RNA}\frac{dn}{dc_{RNA}}$ term to represent a buffer-specific refractive index increment for RNA as

$$\frac{\widehat{dn}}{dc_{RNA}} \equiv \frac{dn}{dc_2} + \sum_{j=3}^{N=6} c_j \nu_{2j}. \tag{14.15}$$

This accounts for the first eleven 2nd-order terms. The remaining ten are proportional to neither c_{FUS} nor c_{RNA}, and may be combined with the refractive index of the solvent and the linear terms for the buffer components to represent the refractive index of the buffer itself as

$$n_{buffer} \equiv n_{solvent} + \sum_{i=3}^{N=6} c_i \frac{dn}{dc_i} + \sum_{i=3}^{N=6} \sum_{j \geq i} c_i c_j \nu_{ij}. \tag{14.16}$$

Combining the FUS/RNA cross-term with Eqs. (14.14-14.16), the quadratic model for the FUS/RNA mixture with explicit buffer components becomes

$$n_{mix} = n_{buffer} + c_{FUS}\frac{\widehat{dn}}{dc_{FUS}} + c_{RNA}\frac{\widehat{dn}}{dc_{RNA}} + c_{FUS}c_{RNA}\frac{d^2n}{dc_{FUS}dc_{RNA}}, \tag{14.17}$$

which has the same form as Eq. (14.10) above, as claimed.

This equivalence demonstrates that describing homogeneous FUS/RNA mixtures in buffer as effective ternary mixtures for analysis of the FUS/RNA cross-term does not impose additional strong assumptions on the optical behavior of the buffer components. In particular, it does not require assuming that the eighteen buffer-related 2nd-order terms are zero. In this context, modeling the system as an effective ternary mixture is intended primarily to simplify communication rather than to further simplify the description of the underlying physics.

Supplementary Note 14.3: Cross-term coefficient calculation

In this section, we describe the procedure used to quantify the cross-term coefficient $\frac{d^2n}{dc_{FUS}dc_{RNA}}$ and generate the plots presented in **Extended Data Fig. 5**.

First, we constructed a quadratic model for the excess absorbance of a hypothetical homogeneous "test mixture" of FUS and RNA with c_{FUS} = c_{RNA} = 1 mg/ml over an L = 10 mm pathlength as

$$A_{excess}(\lambda) = a_{Int}c_{FUS}c_{RNA}S_{Int}(\lambda). \tag{14.18}$$

Here, a_{Int} is the fitted slope of the residual absorbance at 260 nm (**Extended Data Fig. 4**) rescaled by the molecular weight of FUS to be in units of AU × $(\mathrm{mg/mL\,FUS})^{-1}$ × $(\mathrm{mg/mL\,RNA})^{-1}$, and $S_{Int}(\lambda)$ is a normalized reference trace for the interaction spectra. $S_{Int}(\lambda)$ was obtained from the absorption spectra of a homogeneous mixture of 1.2 mg/ml RNA with 5.4 µM FUS following subtraction of a linear model and normalizing the difference such that $S_{Int}(260\text{ nm})$ = 1. We converted $A_{excess}(\lambda)$ to $\kappa_{excess}(\lambda)$ and $\kappa_{excess}(\omega)$ using Eqs. (14.1-14.2) above (**Extended Data Fig. 5a,b**).

To avoid potential issues in calculating the Cauchy principal value from spectra acquired at discrete frequencies [41], we approximate $\kappa_{excess}(\omega)$ as a continuous function by fitting the excess extinction to an analytic model for the frequency-dependent dissipation of two driven linearly-damped linear oscillators (2-oscillator model):

$$\chi''_{\text{2-osc.}}(\omega) = Z_1\frac{\Gamma_1\omega}{(\omega_{0,1}^2-\omega^2)^2+(\Gamma_1\omega)^2} + Z_2\frac{\Gamma_2\omega}{(\omega_{0,2}^2-\omega^2)^2+(\Gamma_2\omega)^2}, \tag{14.19}$$

where $\omega_{0,i}$ and Γ_i are the natural frequency and damping coefficients for the ith oscillator. Z_i characterizes the strength of the ith oscillator and, in the context of a classical mechanical oscillator, is given by the ratio of driving force to oscillator mass. We note that while the physical origin of the resonances in the current context are quantum mechanical rather than classical, simple oscillator models still provide a reasonably accurate quantitative description

of optical dispersion and are the basis of Cauchy and Sellmeier dispersion formulas [42] still in use today [15]. We also note that, due to the disparate scales of κ_{excess} and ω, x- and y-values were rescaled by 1×10^{14} and 3×10^{-7}, respectively, for the fitting. Fitted coefficients were then carefully rescaled afterwards to return to the original units (**Extended Data Fig. 5b**). To avoid bias from spectral features not captured by the 2-oscillator model, fitting was restricted to frequencies corresponding to 205-300 nm. To determine reasonable parameter values with which to initialize the 2-oscillator model for fitting, the rescaled κ_{excess} was first fit to single oscillator models on frequencies corresponding to 250-300 nm (oscillator 1) and 200-230 nm (oscillator 2).

The real component of the excess optical response function (**Extended Data Fig. 5c**) was computed in two ways. First, $\chi''_{\text{2-osc.}}$ and ω were rescaled by $\tilde{Z}_1 = Z_1/\omega_{0,1}^2$ and $\omega_{0,1}$, respectively, to yield the imaginary component of the "reduced" excess optical response function, $\tilde{\chi}''(\omega/\omega_{0,1})$. The real component, $\tilde{\chi}'(\omega/\omega_{0,1})$, was calculated from Eq. (14.8) via symbolic integration in MATLAB using int.m with the 'PrincipalValue' flag set to 'true' followed by conversion of the symbolic output to a numerical result via variable-precision arithmetic using vpa.m. As a second approach, we used the fitted parameter values for the dissipative component of the 2-oscillator model to calculate the frequency-dependent elastic component of the 2-oscillator model

$$\chi'_{\text{2-osc.}}(\omega) = Z_1 \frac{(\omega_{0,1}^2-\omega^2)}{(\omega_{0,1}^2-\omega^2)^2+(\Gamma_1\omega)^2} + Z_2 \frac{(\omega_{0,2}^2-\omega^2)}{(\omega_{0,2}^2-\omega^2)^2+(\Gamma_2\omega)^2}, \quad (14.20)$$

which is shown in reduced form following rescaling in **Extended Data Fig. 5c**. The results of both calculations agree well (**Extended Data Fig. 5c**). The frequency-dependent excess extinction in nominal units was then calculated from $\tilde{\chi}'$ by undoing the previous rescaling of according to

$$n_{excess}(\omega) = \frac{Z_1}{\omega_{0,1}^2}\tilde{\chi}'(\omega). \quad (14.21)$$

This is plotted following conversion from angular frequency back to wavelength in **Extended Data Fig. 5d**. Finally, the value of the refractive cross-term coefficient at the wavelength used for QPI measurements (650 nm) was calculated from the excess refractive index in Eq. (14.21) as $\nu_{ij}(650\text{ nm}) \equiv \frac{d^2n}{dc_i dc_j}(650\text{ nm}) = \frac{n_{excess}(650\text{ nm})}{c_{FUS}c_{RNA}}$.

Supplementary Note 15: Extended description of absolute protein quantification by mass spectrometry

Aliquots were diluted (1:1 v/v) with 2x Laemmli sample buffer (Serva Electrophoresis GmbH, Catalog No. 42526.01) and subjected to 1D SDS polyacrylamide gel electrophoresis using a Mini-PROTEAN system, (BioRad Laboratories). Proteins were visualized by Coomassie staining and the gel regions corresponding to their expected relative molar mass M_r were excised and *in-gel* digested with trypsin (Promega, Germany) [43]. Proteins were quantified by the method of MS Western [44] using an isotopically-labelled chimeric protein standard comprising quantotypic peptides from mEGFP (2 peptides), SNAP (3 peptides) and from the reference protein BSA (5 peptides).

LC-MS/MS was performed on an RSLC system UltiMate 3000-series coupled with a Q Exactive HF mass spectrometer (Thermo Fisher Scientific, Bremen). Peptides were separated on a 75 µm × 2 cm Acclam PepMap 100 trapping column and a 1mm × 50 cm µPAC analytical column (Thermo Fisher Scientific). After loading of the sample onto the trapping column, elution was performed over 75 min at a 400-nL/min flowrate using a gradient of 0.1% aqueous formic acid (solvent A) and 0.1 % formic acid in acetonitrile (solvent B) as follows: 0 to 30% B in "A plus B mixture" for 45 min; 30% to 50% B for 13 min; 50% to 100% B for 2 min and 100% B for 5 min. The column was then re-equilibrated by delivering 100% A for 10 min. Mass spectra were acquired in parallel reaction monitoring (PRM) mode using a full MS1 scan in the range of *m/z* 350-1700 at the targeted mass resolution $R_{s\ m/z=200}$ of 240 000; Automatic gain control (AGC) set to 3×10^6; maximum injection time (IT) set to 150 ms. MS2 spectra were acquired using a precursor isolation window of 3.0 Th; target mass resolution $R_{s\ m/z=200}$ of 120 000; AGC 1×10^5 and IT 200 ms. Spectra acquisition was navigated by the inclusion list containing *m/z* and retention times of precursor ions of (^{13}C,^{15}N)-labeled and native proteotypic peptides from mEGFP, SNAP and BSA proteins (**Table S2**). Spectra were processed by FragPipe software suite v.17.1 (https://fragpipe.nesvilab.org). Molar abundances of quantotypic peptide were calculated as described [44]. Briefly, peak areas on extracted ion chromatograms (XIC) of precursor *m/z* of native peptides were compared to the areas of corresponding isotopically-labelled peptides produced by digestion of the known amount of chimera protein standard, which in turn was referenced to the known amount of the Pierce BSA standard (catalog number 23209, Thermo Fisher Scientific). Each proteotypic peptide was quantified independently. To calculate the molar abundance of proteins, the molar abundances of the corresponding peptides were averaged.

For identification of protein species, tryptic digests of the corresponding gel regions of the reference samples (**Extended Data Fig. 7**) were analysed by LC-MS/MS in Data Dependent Acquisition (DDA) mode. The acquired spectra were matched with MASCOT software (v.2.2.04, Matrix Science, UK) against FUS and TAF fusion sequences with 5ppm and 0.025Da mass accuracy for precursor and fragments respectively.

Supplementary Note 16: Compositional uncertainty in multi-component condensates

To estimate the number of components potentially resolvable in experiments with ATRI, we analyzed how compositional uncertainty varies with system complexity. From measurements of FUS-based condensates in which we resolve N = 1, 2, and 5 solutes, the relative uncertainty in the dense-phase concentration decreases as the distance $\bar{c} - c^{I}$ into the two-phase region increases (**Fig. S15**). This is consistent with the intuition that uncertainty in the tie-line slope is limiting for systems prepared close to the binodal. For a given distance into the two-phase region, the relative dense-phase uncertainty increases with the number of solutes resolved. This is consistent with the intuition that the total uncertainty grows as the number of measurements used in the decomposition increases. Based on these observations, we make the scaling ansatz that the relative dense-phase uncertainty is approximately proportional to the number of resolved solutes and inversely proportional to the distance from the system average composition to the dilute binodal: $\frac{\delta c^{II}}{c^{II}} \approx \frac{aN}{\bar{c}-c^{I}}$. While other factors certainly contribute as well, a global fit to these data captures the trends reasonably well and yields a value for the proportionality constant of a = 0.0125. By inverting this fitted scaling relationship, we can ask how many components can be resolved with a given level of uncertainty. For an uncertainty threshold of 50%, the scaling ansatz predicts that the number of resolvable components with this methodology at a distance of 1 mg/ml from the binodal is 40. As the distance from the binodal increases, this analysis suggests that the maximum number of resolvable components also increases.

Supplementary Note 17: Physical requirements and associated limitations of QPI and ATRI

A natural question upon encountering a new technique with relevance to one's own research is "Will this work for my system of interest?" The purpose of this Supplementary Note is to help readers assess this question. We do this in two parts. First we state the primary physical requirements of the quantitative phase imaging (QPI) and Analysis of Tie-line and Refractive Index (ATRI) methods described in this work. Second, we elaborate on several practical limitations which arise from these physical requirements. We hope that this note will aid implementation of these methods by others.

The QPI and ATRI methods described in this work to determine the molecular composition of biomolecular condensates rely on the following four physical requirements:

1. there exists optical contrast between the condensate and coexisting dilute phase,
2. the condensate's optical response, as measured by QPI, is well-described by a spherical cap of homogeneous fluid,
3. the condensate's refractive index is well-described by a linear sum of contributions from the relevant solutes, each with known refractive index increment (dn/dc), and
4. the dilute-phase composition of all relevant solutes is known to adequate precision.

Each of these physical requirements gives rise to practical limitations, which we discuss in turn below. We note that while all four requirements apply to multi-component condensates, the first three requirements are sufficient for condensates in effective binary mixtures (e.g. one protein species in buffer). In this sense, the fourth requirement is specific to the ATRI method and multi-component systems.

First, the requirement of optical contrast means that the refractive index difference between the two co-existing phases must be non-zero. If a condensate is visible via QPI, then this requirement is satisfied. The smallest Δn measured in this work is $\sim 3 \times 10^{-3}$ (**Fig. 1i**), though we anticipate that a Δn of 1×10^{-4} and perhaps smaller is measurable for a 10-µm non-wetting droplet. In our experience, this requirement is not likely to limit application to reconstituted binary systems where we typically observe $\Delta n > 1 \times 10^{-2}$. As we discuss in the derivations of **Supplementary Note 12**, however, it can lead to limitations for certain regions of segregative multi-component systems as well as some cellular condensates. Specifically, the refractive index difference vanishes in these systems if the tie-line and isorefractive line are

coincident. In such cases, the system of linear equations is singular and the solution given by Eq. (4) in the Main Text does not apply (see **Supplementary Note 12** for further details). When present at all, this indeterminacy is restricted to only a portion of the phase diagram as the slopes of tie-lines typically vary across a phase diagram while the slopes of isorefractive lines are fixed for a given system. Moving the system to a tie-line with a different slope is thus sufficient to restore optical contrast and, with it, the ability to resolve condensate composition.

The second requirement is that the phase image measured by QPI is well-described by a homogeneous spherical cap. In practice, this requirement can lead to restrictions on condensate size, morphology, architecture, molecular ordering, optical absorption, and magnitude of refractive index difference at the imaging wavelength. Deviations due to gravitational settling set the maximum condensate size, which depends on density and interfacial tension (**Fig. S5**), while optical scattering limits accuracy at sizes comparable to the imaging wavelength [45]. We typically have good success with condensate radii in the range of ∼2 – 15 μm, which is compatible with typical reconstitution experiments. Irregular wetting of the substrate, contact with neighboring objects, and incomplete fusion can lead to deviations in condensate shape from that of a spherical cap. Individual condensates with non-circular morphologies (as viewed in 2D images) are best excluded from compositional analysis. Condensates forming core-shell architectures are inhomogeneous and thus not suitable for the QPI analysis presented in this work. Caution is advised for analysis of condensates with liquid-crystalline ordering [46,47] or containing molecules (e.g. dyes) which absorb light strongly near the imaging wavelength as birefringence and the inter-relation of absorption with refraction via Kramers-Kronig relations (**Extended Data Fig. 5, Supplementary Note 14**) could complicate interpretation. Finally, refraction may distort condensate shape in a phase image, particularly for large Δn (**Supplementary Note 3**). When imaged near the equatorial plane, we find that these distortions are negligible for Δn at least as large ∼0.085 (**Fig. 1j**).

The third requirement is that the refractive index of the condensate is well-described by a linear sum of contributions from each of the relevant solutes and that the refractive index increments for those solutes are known. Together with previous work [15,17,20,21,29,30], our observations suggest that the most prominent condensate components, namely proteins and RNA, typically meet this requirement (**Fig. 1k, Fig. S2, Fig. S3, Fig. S4, Extended Data Fig. 5, Extended Data Fig. 6**). If a solute doesn't meet this requirement, condensates containing it may not be suitable for the linear analysis by QPI and ATRI described in the Main Text. In such cases, we anticipate that a non-linear analysis will typically be possible (**Extended Data**

Fig. 5, Extended Data Fig. 6, Supplementary Note 13, Supplementary Note 14) and would likely reduce systematic errors relative to a linear analysis.

As we discuss in **Supplementary Note 4**, a solution's refractive index depends non-linearly on solute concentration in the general case. Some solutes may therefore contribute a non-linear response over the relevant regime. In such cases, application of Eq. (4) of the Main Text could lead to significant errors. In the context of a quadratic model for the refractive index of a multi-component mixture (**Supplementary Note 13**), there are two potential sources. The first would be a large intrinsic non-linearity (i.e. a large d^2n/dc^2 term). We are not aware of data demonstrating a significant contribution from this term for a biological molecule. We caution that the choice of concentration scale matters when assessing linearity, and that the refractive index of a solution that varies non-linearly as a function of solute mass fraction can be well-described as a linear function of solute concentration (**Fig. S2**). Solute concentration in mg/ml was used for all refractive index analysis in this work. In addition to proteins and RNA, we find a linear concentration dependence for the crowding agents PEG and dextran (**Fig. 1k**). We note that dn/dc has been measured previously for many common salts [48], and can be determined for several other solutes from data in the CRC Handbook of Chemistry and Physics [49].

Alternatively, the non-linearity could stem from a large cross-term coefficient (i.e. large $d^2n/dc_i dc_j$) owing to interactions between two solutes. We observe a modest cross-term coefficient for the RNA-binding protein FUS and polyA RNA (**Extended Data Fig. 5**), though find that neglecting it introduces a systematic error of 4% or less (**Extended Data Fig. 5, Extended Data Fig. 6**). In such cases, the solute in question can either be avoided, the systematic error tolerated, or the non-linearity accounted for, as in the non-linear ATRI analysis associated with **Extended Data Fig. 6**.

In principle, the accurate determination of dn/dc for a solute of interest can also represent a limitation related to the third requirement. For solutes with large miscibility gaps like the protein PGL-3, it may be impossible to prepare homogeneous solutions over the concentration range necessary to measure dn/dc by titration (**Supplementary Note 2**). For miscible but particularly precious reagents, such measurements may simply be costly. For proteins, we note that the refractive index increment depends only weakly on structure (see also **Supplementary Note 9**) and may be estimated to high accuracy directly from sequence [15]. Further, the standard deviation of dn/dc across all sequences in the human proteome is below 2 % [15], suggesting that the error incurred by approximating the dn/dc of a particular protein with the proteome

average (e.g. 0.1899 ml/g at 589.3 nm and 25 °C) is typically small. Systematic errors may be reduced by applying approximate corrections for the temperature- and wavelength-dependence (see **Extended Data Fig. 5**, **Fig. S7**, and [15]). For proteins with post-translational modifications, the extra atoms on a modified residue will alter the refractivity relative to the unmodified amino acid. As the molar refraction tends to increase with the molar mass [15,50], we predict that PTMs will typically lead to an increase in protein dn/dc. For heavily-modified proteins, it may be desirable to account for the modifications when calculating dn/dc. We anticipate that adequate knowledge of dn/dc will only rarely pose limitations in practice.

The fourth requirement, which applies specifically to multi-component systems, is that the dilute-phase composition of all relevant solutes is known to adequate precision. The magnitude of solute partition coefficients and choice of dilute-phase detection strategies can contribute to systematic errors and limit the number and identity of resolvable components in a condensate.

All solutes which partition unequally between coexisting phases contribute to the refractive index difference measured by QPI. For solutes at very low concentrations and/or with partition coefficients near 1 (e.g. buffer components), the optical contribution may be negligible. For the remaining solutes, users must select (potentially a combination of) dilute-phase detection strategies than can distinguish between the them. While UV-vis spectroscopy can be sufficient to distinguish between protein and RNA (**Fig. 4**), it is not well-suited to distinguish between two unlabeled protein species. Modern analytic methods such as mass spectrometry (MS) and nuclear magnetic resonance (NMR) are powerful tools in this regard. By combining UV-vis spectroscopy and quantitative MS, we resolved 4 related proteins and an RNA for a total of 5 solutes (**Fig. 5**, **Extended Data Fig. 8**, **Extended Data Fig. 10**). For in vitro studies, we anticipate that existing analytic chemistry technologies will suffice to distinguish most biological solutes of interest. To enable ATRI for those cellular condensates with optical contrast, further development of MS and related technologies is likely needed in order achieve adequate sensitivity for concentration measurements of low-abundance solutes in the cyto- and/or nucleoplasm at the single-cell level [51].

As the number of relevant solutes increases, so too does the number of measurements and the cumulative uncertainty in dense-phase composition. The greater the precision in the dilute-phase measurements, the smaller the resolvable partition coefficient of a solute and the greater the number of components which may be meaningfully resolved (**Fig. S14**, **Fig. S15**). While an upper limit on the number of practically resolvable components is unclear, a preliminary

scaling analysis (**Fig. S15**) suggests that composition measurements are likely possible for reconstituted condensates with 40+ components.

SUPPLEMENTARY FIGURES

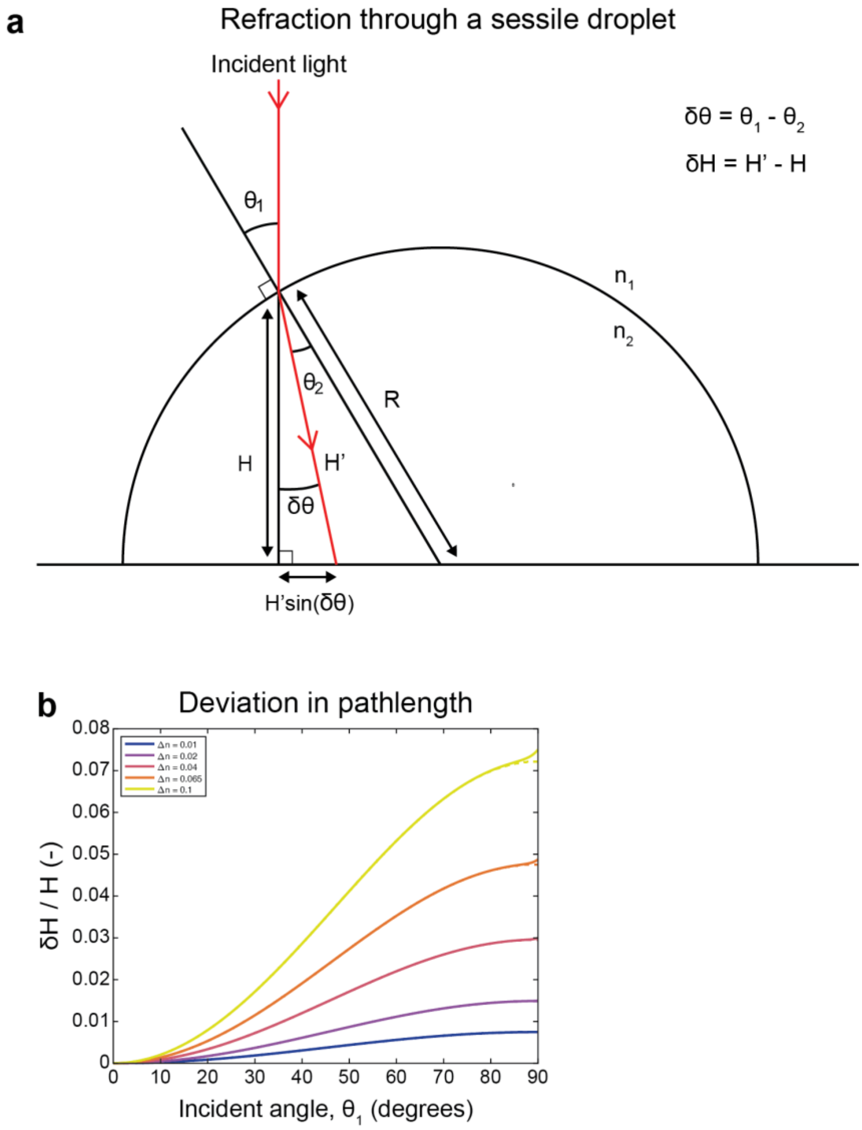

Fig. S1: Straight-line optical path approximation for QPI analysis
a Schematic for analysis of refraction of light by a sessile droplet of radius R on a substrate using ray-tracing in the context of geometric optics. The refractive indices of the droplet and the surrounding medium are denoted by n_2 and n_1, respectively. The angles of incidence and transmission (relative to the surface normal) are given by θ_1 and θ_2, respectively. H denotes the optical pathlength of a hypothetical non-refracted ray, while the pathlength of the refracted ray is $H' = H + \delta H$. Refraction of the incoming ray by the droplet interface alters the path of the transmitted ray by an angle $\delta\theta = \theta_1 - \theta_2$. **b**, The fractional deviation in the optical path length, $\delta H/H$, as a function of incident angle for a range of refractive index mismatches typical for biomolecular condensates (colors). The solid lines denote the exact expression (Eq. (3.7)) while dashed lines give the approximation in Eq. (3.8), valid in the limit that $\tan^2\theta_1(\delta n)^2 \ll 1$. We find that the refraction-induced deviation in optical pathlength increases with incident angle. However, the deviations are typically only a few percent. We expect that the good quantitative agreement we find between bulk refractometry and our QPI analysis (**Fig. 1j**), which neglects these deviations, is possible precisely because these deviations are so small for the low refractive index mismatches typical of condensates.

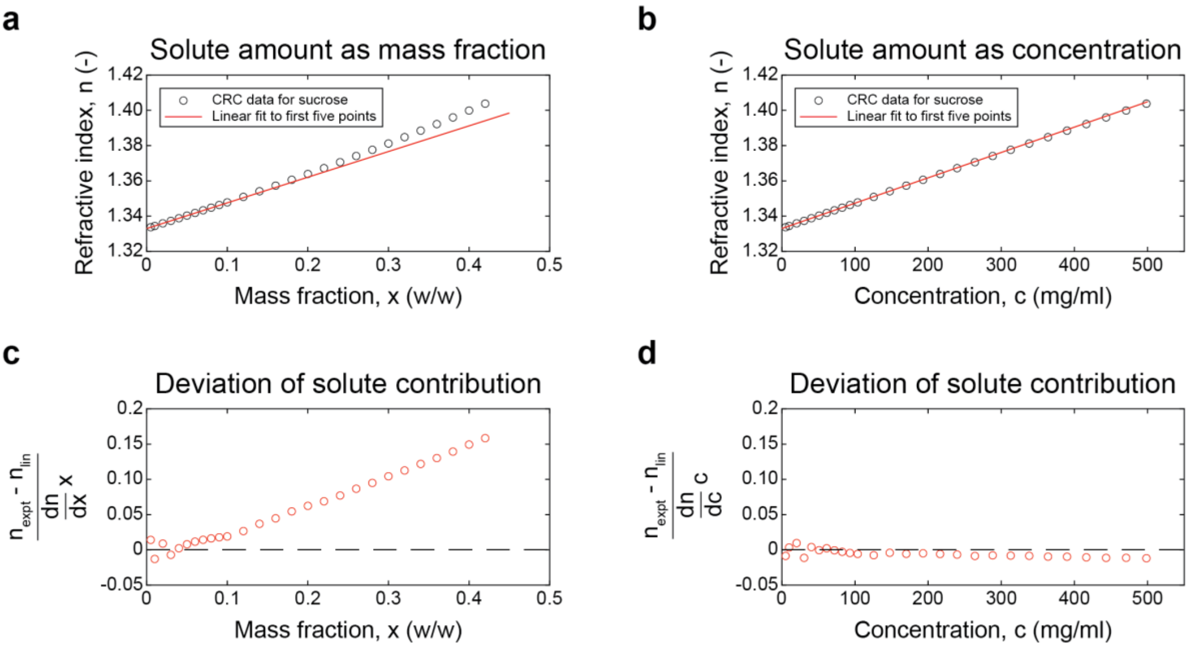

Fig. S2: Impact of solute concentration scale on assessment of refractive index linearity We compare a single set of refractive index measurements of aqueous sucrose solutions in which the solute amount is plotted in units of mass fraction (**a**) or concentration (**b**). Data are taken from the CRC Handbook of Chemistry and Physics and correspond to measurements at a wavelength of 589.3 nm at 20 °C. In each case, a red line is shown extrapolating a fit to the five lowest solute amounts. While significant deviations from linearity are visible when plotted against solute mass fraction (**a**), the same data are well-described as linear when plotted as a function of solute concentration (**b**). To better appreciate the magnitude of the discrepancy, we plot the deviation of the solute contribution to the solution refractive index from the linear model against solute mass fraction (**c**) or concentration (**d**). When plotted against solute mass fraction, the deviation grows as the amount of solute increases, exceeding 2% at x = 0.12 and exceeding 15% at x = 0.42, corresponding to solute concentrations of approximately 125 and 500 mg/ml, respectively. In contrast, deviations from linearity are below 1.2% over the entire range probed when plotted against solute concentration. To reduce systematic errors, compositional analysis of solution refractive index is best performed in units proportional to solute concentration as the linear range is greatest for this unit choice.

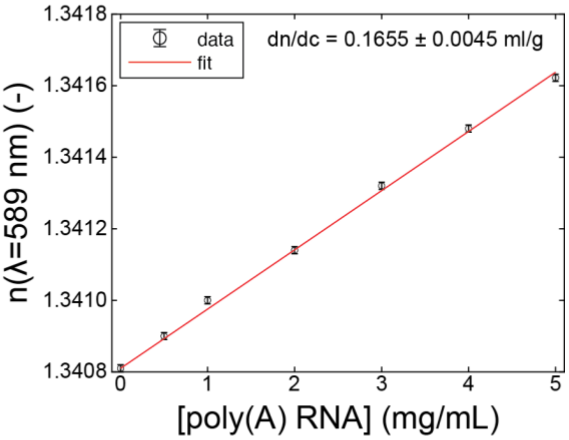

Fig. S3: Refractive index increment of poly(A) RNA at 589 nm
Refractive index of poly(A) RNA in water as a function of RNA concentration. Measurements were performed with a digital refractometer at 589 nm at 20 °C. Datapoints represent the mean of $N = 5$ measurements. Error bars are the larger of the standard deviation of $N = 5$ measurements or the instrument resolution 0.00001 (if all repeat measurements were identical). The refractive index increment is given by a linear fit as 0.1655 ± 0.0045 ml/g.

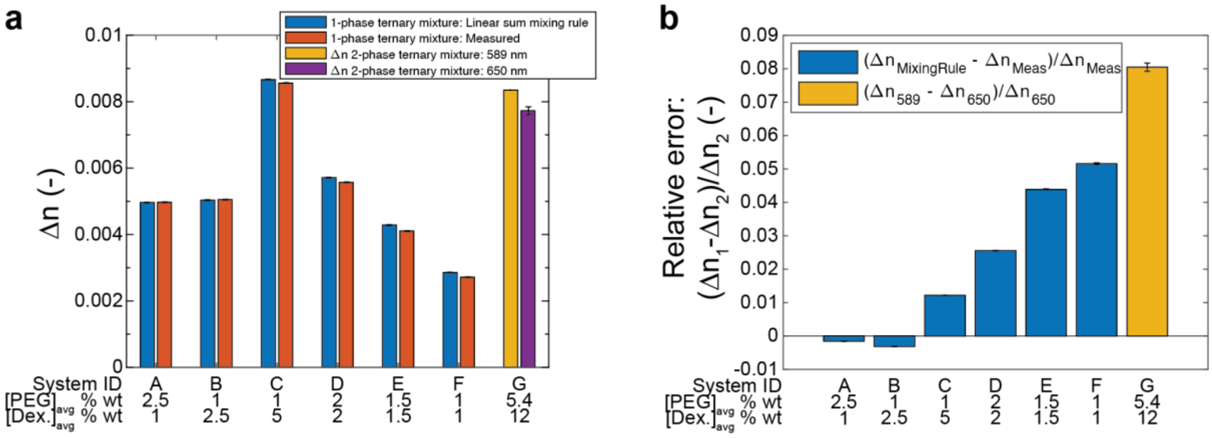

Fig. S4: Validation of linear sum approximation for homogeneous PEG/dextran mixtures
a, Refractive index difference between either homogeneous PEG/dextran mixtures prepared in the one-phase regime and water (blue and red) or between coexisting phases in the two-phase regime (yellow and purple). Average system composition is specified below each bar as weight fractions of PEG-35k and dextran-500k. Blue bars show the refractive index measured experimentally on a digital refractometer at 589 nm, while red bars show the refractive index predicted for the same compositions using a linear sum approximation for the mixing rule (see Methods). Yellow bar shows the difference between the refractive indices of the coexisting phases, each measured individually at 589 nm on a digital refractometer. Purple bar shows the population mean refractive index difference between the same phases measured via QPI for $N = 205$ droplets. Error bars for digital refractometry measurements are the larger of the standard deviation of $N = 5$ measurements or the instrument resolution 0.00001 (if all repeat measurements were identical). Error bars for the predictions are estimated at 0.00001. Error bar for the QPI measurement is the standard deviation of the measured Δn distribution. All measurements were performed at 24 °C. **b**, Relative error of the paired measurements in **a**. The relative error incurred by application of the linear sum mixing rule is typically much less than 5 %. For comparison, natural variation in optical properties with wavelength (dispersion) results in an 8-% change in Δn between 589 nm and 650 nm.

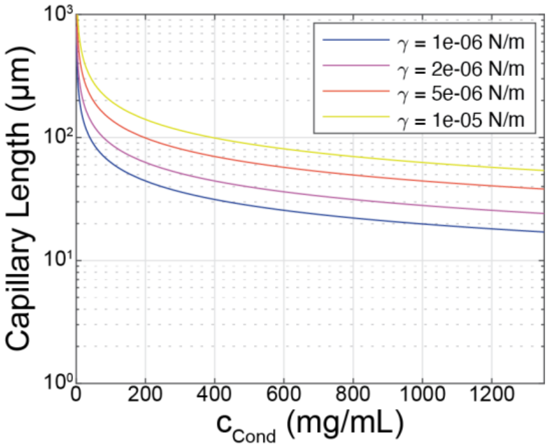

Fig. S5: Estimate of capillary length for biomolecular condensates
Capillary length as a function of the condensed-phase polymer mass concentration. The capillary length increases with increasing interfacial tension, so we plot traces for different values of interfacial tension. From bottom to top, interfacial tensions are 1, 2, 5, and 10 µN/m. This spans the range of tensions reported for PGL-3 condensates by Ref. [52]. For a given droplet density and interfacial tension, the spherical cap approximation for the droplet shape is valid so long as the droplet size is less than the capillary length. In the case of PGL-3, our density measurements of ~ 90 mg/ml indicate a capillary length of 67 µm at the lowest interfacial tension, which is much larger than the 1-8 µm radii of the droplets. Although the capillary length is reduced to ~ 30 µm for the higher ~ 400-500 mg/ml densities we measure for TAF15(RBD) condensates, this length is still much larger than the 0.67-3.2 µm radii of those condensates analyzed in this work.

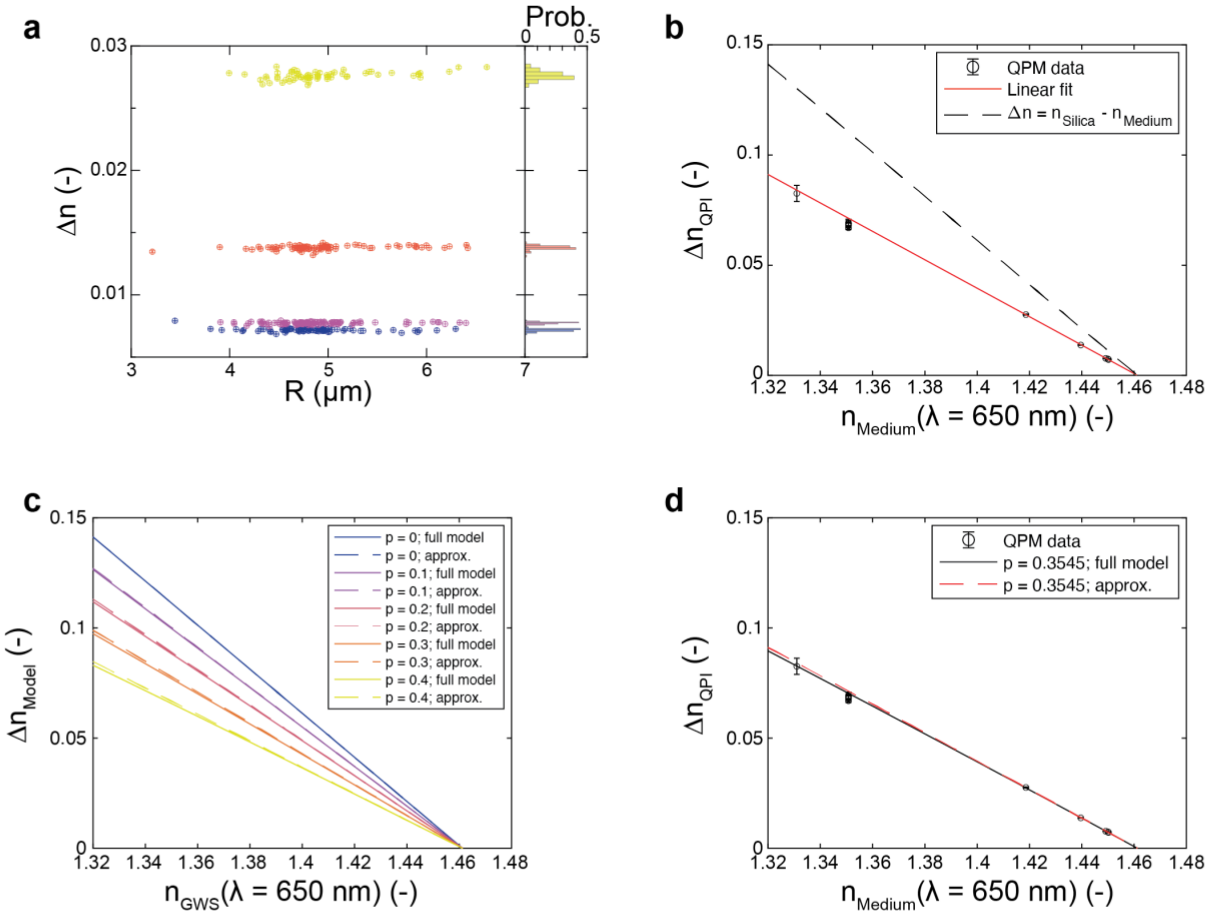

Fig. S6: Silica bead measurements and porosity

a, (left) Refractive index difference between silica beads and the surrounding glycerol/water mixture as a function of bead size as measured by QPI for a range of glycerol/water ratios. Glycerol weight fraction of the medium is 0.6641 (yellow, $N = 66$), 0.808 (orange, $N = 83$), 0.8688 (purple, $N = 102$), and 0.8751 (blue, $N = 74$). Datapoints represent individual beads, error bars are 95% confidence intervals returned from fits, and are typically smaller than the datapoint. Though the nominal bead diameter is 10 μm, the samples show significant polydispersity. Importantly, there is no strong systematic variation of the extracted Δn with bead size. (right) Measured refractive index distribution is approximately symmetric for each condition. **b**, Population mean (± standard deviation) of Δn measured by QPI at λ = 650 nm for silica beads as a function of the refractive index of the glycerol/water mixture, n_{medium}. In order of increasing Δn_{QPI}, N = 62, 71, 92, 77, 57, 7, 16, 8, 10, 56, 63, or 21 microspheres. Note that n_{medium} was measured at λ = 589 nm on a digital refractometer and is plotted following adjustment to λ = 650 nm using published dispersion relationships for glycerol and distilled water and an approximate mixing rule, as described in Methods. Solid red line is a linear fit to the data with the 4 largest x-values, with slope -0.6455 ± 0.01806. The x-intercept of the fit line, at which the refractive index of the silica bead is indistinguishable from that of the medium, is 1.461 ± 0.057. Importantly, this is comparable to published values of fused silica under similar conditions (e.g. n = 1.4565 at 650 nm, 20 °C, [53]), independently of whether we adjust for dispersion. However, the slope of the fit line (-0.6455 ± 0.0181) differs significantly from the value of -1 expected for beads of pure fused silica (dashed black line). This suggests that the beads may be porous, as has been reported previously [54]. **c**, The refractive index difference expected for silica beads with porosity p as a function of the refractive index of the surrounding glycerol/water mixture, assuming that the bead pores are filled with the glycerol/water mixture. Here p is the fraction of the bead volume occupied by pores rather than

silica. Different colors represent different values of porosity from 0 to 0.4. For each p, Δn is calculated using two different refractive index mixing rules, either a weighted linear combination (approx., dashed line) or the Lorentz-Lorenz relation (full model, solid line, Methods). Both models predict that fluid-filled pores in the silica beads would give a Δn that decreases (nearly) linearly with slope (1-p) as n_{medium}. increases. The two models are almost indistinguishable except at low n_{medium} and high p, where the simple approximation noticeably overestimates Δn. **d**, Same data as in **b** above, but now overlaid with model predictions for silica beads with p = 0.3545. All measurements at 25 °C.

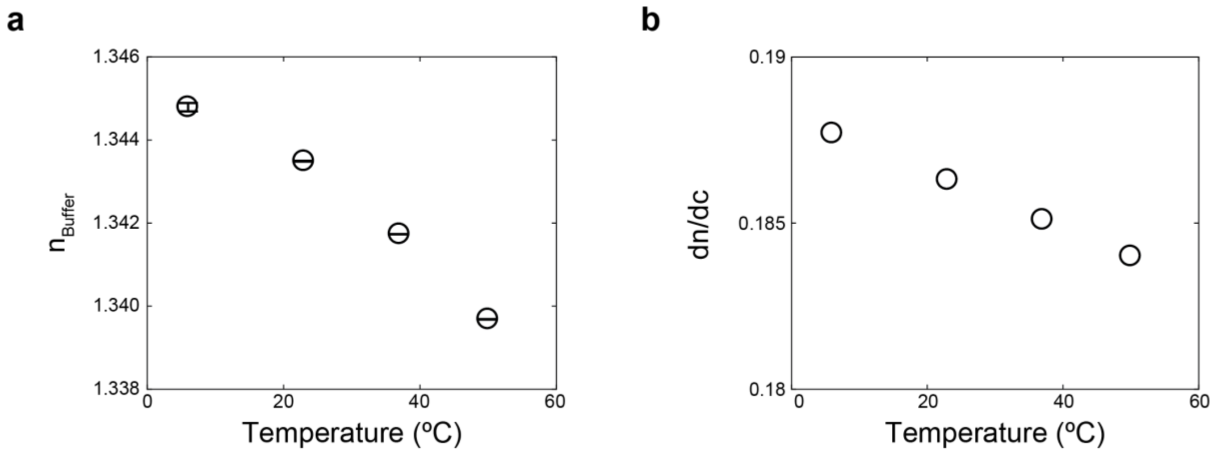

Fig. S7: Temperature-dependent optical constants

a, Refractive index of buffer as measured with a digital refractometer at 589 nm over the temperature range used in **Fig. 2d**. Datapoints represent the mean of $N = 5$ measurements. Error bars are the larger of the standard deviation of $N = 5$ measurements or the instrument resolution 0.00001 (if all repeat measurements were identical). The refractive index of the solution decreases with increasing temperature primarily due to the reduced electron density accompanying thermal expansion. Note that the reduction in refractive index due to this effect, n_{buffer}(6 °C) - n_{buffer}(50 °C) = 0.0051, is much smaller than the variation of Δn we measure for TAF15(RBD) condensates over the same range, Δn(6 °C) - Δn(50 °C) = 0.0144. **b**, Refractive index increment for SNAP-TAF15(RBD) estimated using SEDFIT software [15] as a function of temperature. These values were used in **Fig. 2d** to convert the Δn measured by QPI for SNAP-TAF15(RBD) condensates to the c_{cond} values reported at each temperature.

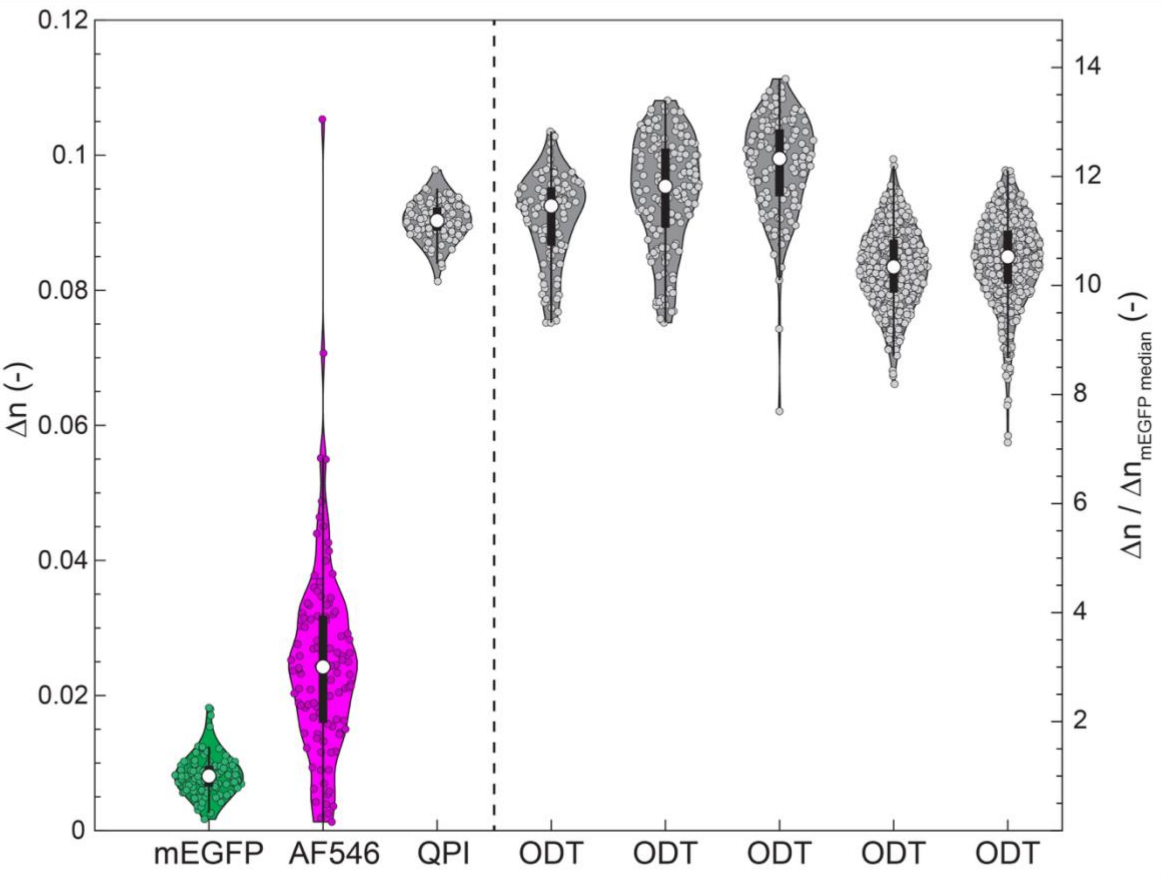

Sample ID	A	B	C	D	E	F	G	H
[KCl] mM	100	100	100	50	100	150	50	150
Temperature °C	22	22	37	23	23	23	23	23
[Tris HCl] mM	50	50	10	10	10	10	50	50
[Glycerol] % v/v	5	5	1	1	1	1	5	5

Fig. S8: Impact of environmental conditions on Δn for SNAP-TAF15(RBD) condensates Comparison of Δn distributions for SNAP-TAF15(RBD) condensates measured by different methods in similar but non-identical solution conditions. For samples A-C, the data from **Fig. 2f** are replotted in terms of Δn. The partition coefficient estimates obtained by fluorescence intensity ratios (samples A and B) were converted to refractive index difference according to $\Delta n = (P_{est} - 1)c_{dil}M_w\frac{dn}{dc}$ using M_w = 62924.3 g/mol, and $\frac{dn}{dc} = 0.1893$ mL/g, and a literature value[55] of $c_{dil} = 1.97$ µM. Distributions A-H contain N = 107, 104, 119, 101, 164, 133, 474, and 385 individual condensates, each from a single sample. For all violin plots, white circles denote medians, thick black bars are the interquartile range, and whiskers extend 1.5x beyond the interquartile range. Although the salt concentrations for samples A-C are identical, we note that the temperature and buffer composition differ. To assess how these differences influence the comparison, we examine ODT measurements (samples D-H) over a range of conditions. As in **Fig. 2c**, the spread is larger for the ODT measurements than for QPI. We find that Δn increases slightly upon temperature decrease from 37 to 23 °C (compare C to E), consistent with the behavior observed for mEGFP-TAF15(RBD) (**Fig. 2d**). Conversely, Δn decreases modestly upon increasing the Tris and glycerol content of the buffer (compare D to G and F to H). These effects thus partially cancel. Importantly, variation in Δn between QPI and ODT values is small relative to the difference between the fluorescence-based estimates (A-B) and any of the QPI or ODT measurements (C-H). In each case, the median Δn from QPI or ODT is larger than that from mEGFP fluorescence at least 10-fold (right y-axes).

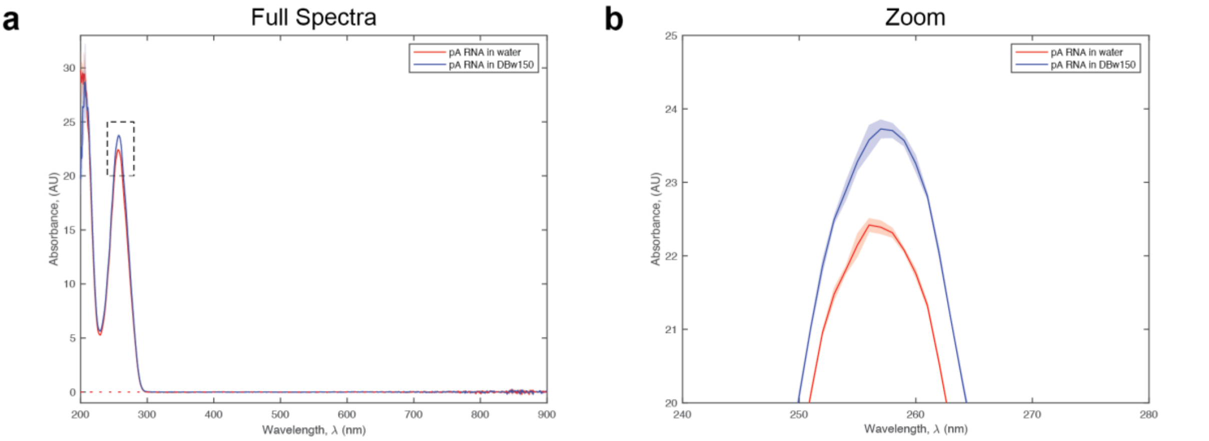

Fig. S9: RNA absorbance is sensitive to solution conditions

a, UV-Vis absorption spectra of equal concentrations of polyA RNA in either pure water (red) or a buffer solution (blue) containing 40 mM Tris HCl (pH 7.4), 150 mM KCl, 1 mM DTT, and 4% (v/v) glycerol. Spectra are shown following subtraction of the absorption spectra of the carrier solution in the absence of RNA. Solid lines represent the average of 5 repeat measurements, while the shaded regions show the standard deviation observed at each wavelength. Red dashed line shows the baseline. In both cases, the spectra are featureless above 300 nm, show a strong peak near 260 nm, a local minimum near 230 nm, and increase into the deep UV. The RNA absorption is consistently higher in the buffer solution relative to water for wavelengths between ~210 and 290 nm. This may be interpreted in terms of an extinction coefficient for RNA in the buffer that is 1.069 ± 0.006 times larger than that in water. **b**, Zoom-in on the region denoted by the black dashed box in **a**, which focuses on the peak near 260 nm used to estimate RNA concentrations. As in **a**, the solid line and shaded bands represent the mean and standard deviation, respectively, of 5 repeat spectral measurements. The separation between the spectra in water and buffer is many-fold larger than the variation between repeats.

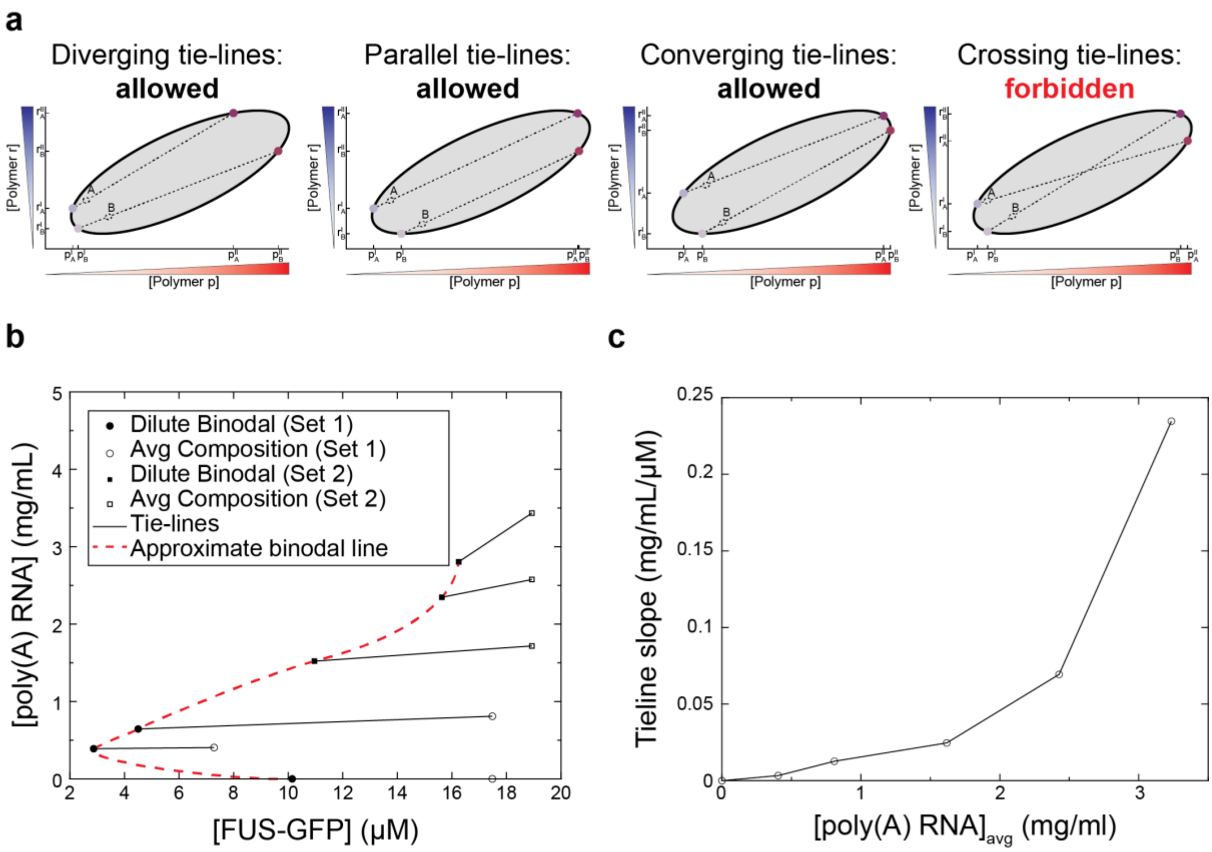

Fig. S10: Examples of physically permissible tie-lines for an associative ternary mixture
a, Examples of ternary phase diagrams with varying tie-line orientations. While tie-lines can in principle diverge (left), be parallel (center-left), or converge (center-right), it is unphysical and thus forbidden for them to cross within the multi-phase coexistence region (right). **b**, Ternary phase diagram for FUS/poly(A) RNA on linear scales zoomed in on the dilute binodal. **c**, Slopes of the tie-lines shown in **b**. Relative to the classification scheme introduced in **a**, this system displays diverging tie-lines.

Lane 2, Gel region 1: MBP-FUS-mEGFP

MKIEEGK**LVI WINGDK**GYNG LAEVGKKFEK DTGIK**VTVEH PDKLEEK**FPQ VAATGDGPDI IFWAHDRFGG YAQSGLLAEI TPDK**AFQDKL YPFTWDAVR**Y NGK**LIAYPIA VEALSLIYNK DLLPNPPK**TW EEIPALDKEL KAKGKSALMF NLQEPYFTWP
LIAADGGYAF KYENGKYDIK DVGVDNAGAK **AGLTFLVDLI K**NKHMNADTD YSIAEAAFNK GETAMTINGP WAWSNIDTSK **VNYGVTVLPT FKGQPSKPFV GVLSAGINAA SPNK**ELAKEF LENYLLTDEG LEAVNKDKPL GAVALKSYEE ELVKDPRIAA
TMENAQK**GEI MPNIPQMSAF WYAVRTAVIN AASGRQTVDE ALK**DAQTNSS SNNNNNNNNN NSSGRLEVLF QGPAAAMASN DYTQQATQSY GAYPTQPGQG YSQQSSQPYG QQSYSGYSQS TDTSGYGQSS YSSYGQSQNT GYGTQSTPQG YGSTGGYGSS
QSSQSSYGQQ SSYPGYGQQP APSSTSGSYG SSSQSSSYGQ PQSGSYSQQP SYGGQQQSYG QQQSYNPPQG YGQQNQYNSS SGGGGGGGGG GNYGQDQSSM SSGGGSGGGY GNQDQSGGGG SGGYGQQASD RGGRGRGGSG GGGGGGGGGY NRSSGGYEPR
GRGGGRGGRG GMGGSDRGGF NKFGGPRDQG SRHDSEQDNS DNNTIFVQGL GENVTIESVA DYFKQIGIIK TNKK**TGQPMI NLYTDR**ETGK **LKGEATVSFD DPPSAKAAID WFDGKEFSGN PIK**VSFATRR ADFNRGGGNG RGGRGRGGPM GRGGYGGGGS
GGGGR**GGFPS GGGGGGGQQR** AGDWKCPNPT CENMNFSWRN ECNQCKAPKP DGPGGGPGGS HMGGNYGDDR RGGRGGYDRG GYRGRGGDRG GFRGGRGGGD RGGFGPGKMD SRGEHRQDRR **ERPYGAPGSA GSAAGSGMVS KGEELFTGVV PILVELDGDV**
NGHKfsvsge gegdatygkL TLKFICTTGK LPVPWPTLVT TLTYGVQCFS RYPDHMKQHD FFK**SAMPEGY VQERTIFFKD DGNYK**TR**AEV Kfegdtlvnr ielk**GIDFKE DGNILGHKLE YNYNSHNVYI MADKQKNGIK VNFKIRHNIE DGSVQLADHY
QQNTPIGDGP VLLPDNHYLS TQSKLSKDPN EKRDHMVLLE FVTAAGITLG MDELYKLEVL FQGPGSSHHH HHHSG

Lane 2, Gel region 2: FUS-mEGFP

MKIEEGKLVI WINGDKGYNG LAEVGKKFEK DTGIKVTVEH PDKLEEKFPQ VAATGDGPDI IFWAHDRFGG YAQSGLLAEI TPDKAFQDKL YPFTWDAVRY NGKLIAYPIA VEALSLIYNK DLLPNPPKTW EEIPALDKEL KAKGKSALMF NLQEPYFTWP
LIAADGGYAF KYENGKYDIK DVGVDNAGAK AGLTFLVDLI KNKHMNADTD YSIAEAAFNK GETAMTINGP WAWSNIDTSK VNYGVTVLPT FKGQPSKPFV GVLSAGINAA SPNKELAKEF LENYLLTDEG LEAVNKDKPL GAVALKSYEE ELVKDPRIAA
TMENAQKGEI MPNIPQMSAF WYAVR**TAVIN AASGR**QTVDE ALKDAQTNSS SNNNNNNNNN NSSGRLEVLF QGPAAAMASN DYTQQATQSY GAYPTQPGQG YSQQSSQPYG QQSYSGYSQS TDTSGYGQSS YSSYGQSQNT GYGTQSTPQG YGSTGGYGSS
QSSQSSYGQQ SSYPGYGQQP APSSTSGSYG SSSQSSSYGQ PQSGSYSQQP SYGGQQQSYG QQQSYNPPQG YGQQNQYNSS SGGGGGGGGG GNYGQDQSSM SSGGGSGGGY GNQDQSGGGG SGGYGQQASD RGGRGRGGSG GGGGGGGGGY NRSSGGYEPR
GRGGGRGGRG GMGGSDRGGF NKFGGPRDQG SRHDSEQDNS DNNTIFVQGL GENVTIESVA DYFKQIGIIK TNK**KTGQPMI NLYTDRETGK LKGEATVSFD DPPSAKAAID WFDGKEFSGN PIK**VSFATRR ADFNRGGGNG RGGRGRGGPM GRGGYGGGGS
GGGGR**GGFPS GGGGGGGQQR** AGDWKCPNPT CENMNFSWRN ECNQCKAPKP DGPGGGPGGS HMGGNYGDDR RGGRGGYDRG GYRGRGGDRG GFRGGRGGGD RGGFGPGKMD SRGEHRQDRR **ERPYGAPGSA GSAAGSGMVS KGEELFTGVV PILVELDGDV**
NGHKfsvsge gegdatygkL TLKFICTTGK LPVPWPTLVT TLTYGVQCFS RYPDHMK**QHD FFKSAMPEGY VQERTIFFKD DGNYK**TR**AEV Kfegdtlvnr ielkGIDFKE DGNILGHKLE YNYNSHNVYI MADK**QKNGIK VNFKIRHNIE DGSVQLADHY
QQNTPIGDGP VLLPDNHYLS TQSKLSKDPN EKRDHMVLLE FVTAAGITLG MDELYKLEVL FQGPGSSHHH HHHSG

Lane 2, Gel region 3: shortFUS-mEGFP

MKIEEGKLVI WINGDKGYNG LAEVGKKFEK DTGIKVTVEH PDKLEEKFPQ VAATGDGPDI IFWAHDRFGG YAQSGLLAEI TPDKAFQDKL YPFTWDAVRY NGKLIAYPIA VEALSLIYNK DLLPNPPKTW EEIPALDKEL KAKGKSALMF NLQEPYFTWP
LIAADGGYAF KYENGKYDIK DVGVDNAGAK AGLTFLVDLI KNKHMNADTD YSIAEAAFNK GETAMTINGP WAWSNIDTSK VNYGVTVLPT FKGQPSKPFV GVLSAGINAA SPNKELAKEF LENYLLTDEG LEAVNKDKPL GAVALKSYEE ELVKDPRIAA
TMENAQKGEI MPNIPQMSAF WYAVRTAVIN AASGRQTVDE ALKDAQTNSS SNNNNNNNNN NSSGRLEVLF QGPAAAMASN DYTQQATQSY GAYPTQPGQG YSQQSSQPYG QQSYSGYSQS TDTSGYGQSS YSSYGQSQNT GYGTQSTPQG YGSTGGYGSS
QSSQSSYGQQ SSYPGYGQQP APSSTSGSYG SSSQSSSYGQ PQSGSYSQQP SYGGQQQSYG QQQSYNPPQG YGQQNQYNSS SGGGGGGGGG GNYGQDQSSM SSGGGSGGGY GNQDQSGGGG SGGYGQQASD RGGRGRGGSG GGGGGGGGGY NRSSGGYEPR
GRGGGRGGRG GMGGSDRGGF NKFGGPRDQG SRHDSEQDNS DNNTIFVQGL GENVTIESVA DYFKQIGIIK TNKK**TGQPMI NLYTDRETGK LKGEATVSFD DPPSAKAAID WFDGKEFSGN PIK**VSFATRR ADFNRGGGNG RGGRGRGGPM GRGGYGGGGS
GGGGR**GGFPS GGGGGGGQQR** AGDWKCPNPT CENMNFSWRN ECNQCKAPKP DGPGGGPGGS HMGGNYGDDR RGGRGGYDRG GYRGRGGDRG GFRGGRGGGD RGGFGPGKMD SRGEHRQDRR **ERPYGAPGSA GSAAGSGMVS KGEELFTGVV PILVELDGDV**
NGHKfsvsge gegdatygkL TLKFICTTGK LPVPWPTLVT TLTYGVQCFS RYPDHMKQHD FFK**SAMPEGY VQERTIFFKD DGNYK**TR**AEV Kfegdtlvnr ielkGIDFKE DGNILGHK**LE YNYNSHNVYI MADKQKNGIK VNFKIRHNIE DGSVQLADHY
QQNTPIGDGP VLLPDNHYLS TQSKLSKDPN EKRDHMVLLE FVTAAGITLG MDELYKLEVL FQGPGSSHHH HHHSG

Lane 3, Gel region 3: SNAP-TAF15(RBD)

*
GPMDKDCEMK R**ttldsplgk** LELSGCEQGL HRIIFLGKGT SAADAVEVPA PAAVLGGPEP LMQATAWLNA YFHQPEAIEE FPVPALHHPV FQQESFTRQV LWKLLKVVKf gevisyshla alagnpaata avkTALSGNP VPILIPCHR**v vqgdldvggy**
egglavkEWL LAHEGHRLGK PGLGGSSSGR **ENLYFQGAAA GR**GRGGYDKD GRGPMTGSSG GDRGGFKNFG GHRDYGPRTD ADSESDNSDN NTIFVQGLGE GVSTDQVGEF FKQIGIIKTN K**KTGKPMINL YTDK**DTGKPK **GEATVSFDDP PSAKAAIDWF**
DGKEFHGNII KVSFATRRPE FMRGGGSGGG RRGRGGYRGR GGFQGRGGDP KSGDWVCPNP SCGNMNFARR NSCNQCNEPR PEDSRPSGGD FRGRGYGGER GYRGRGGRGG DRGGYGGDRS GGGYGGDRSS GGGYSGDRSG GGYGGDRSGG GYGGDRGGGY
GGDRGGGYGG DRGGGYGGDR GGYGGDRGGG YGGDRGGYGG DRGGYGGDRG GYGGDRGGYG GDRSRGGYGG DRGGGSGYGG DRSGGYGGDR SGGGYGGDRG GGYGGDRGGY GGKMGGRNDY RNDQRNRPYG AP

Fig. S11: Identification of protein species by mass spectrometry
Gel lanes and regions refer to **Extended Data Fig. 7**. Peptides detected by mass spectrometry are shown in RED **Bold**. Sequence of the MBP-FUS-mEGFP fusion comprises MBP (position 5-379, shown in *Italic*), FUS (397-924) and mEGFP (938-1176, underlined). Sequence of the SNAP-TAF fusion includes SNAP (position 3-184, underlined) and TAF (202–609). Quantotypic mEGFP and SNAP peptides used for absolute quantification are shown as lower case; “*” – peptides detected only in PRM mode.

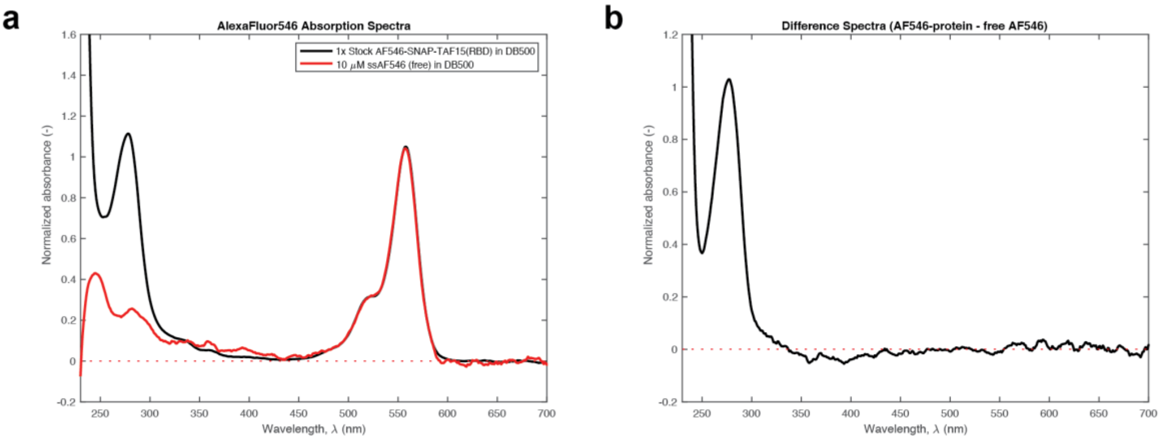

Fig. S12: Contribution of dye to AF546-SNAP-TAF15(RBD) absorption spectra
a, Normalized UV-Vis spectra of SNAP-TAF15(RBD) labeled with the dye AF546 (black) and of 10 uM free dye (red). Spectrum of a buffer "blank" was subtracted prior to normalization in each case. For the protein spectrum, the blank was a solution of storage buffer (50 mM Tris HCl, pH 7.4, 500 mM KCl, 1 mM DTT, 5 % (v/v) glycerol). For the free dye, the blank was storage buffer supplemented with 1 % methanol to account for the carrier solution in which the dye is stored. Spectra were normalized by the value at 554 nm, near the dye absorption peak. A spectral baseline of zero absorbance (dashed red line) is shown as a guide to the eye. The AF546 dye absorbs significantly near the 260-nm and 280-nm wavelengths used to infer the protein and RNA content in mixtures. **b**, Normalized UV-Vis spectrum for unlabeled ("black") SNAP-TAF15(RBD) in storage buffer. This spectrum was obtained following subtraction of the dye spectra from that of the labeled protein in **a**, and subsequently normalizing the difference spectra by the value at 280 nm. As expected for spectra of pure unlabeled protein, this spectrum shows a prominent peak near 280 nm, a 260/280 absorbance ratio of 0.58 < 0.6, and no strong features at longer wavelengths. This spectrum was used as a reference spectrum for black SNAP-TAF15(RBD) in the compositional analysis of dilute-phase spectra for the FUS/TAF15/RNA system.

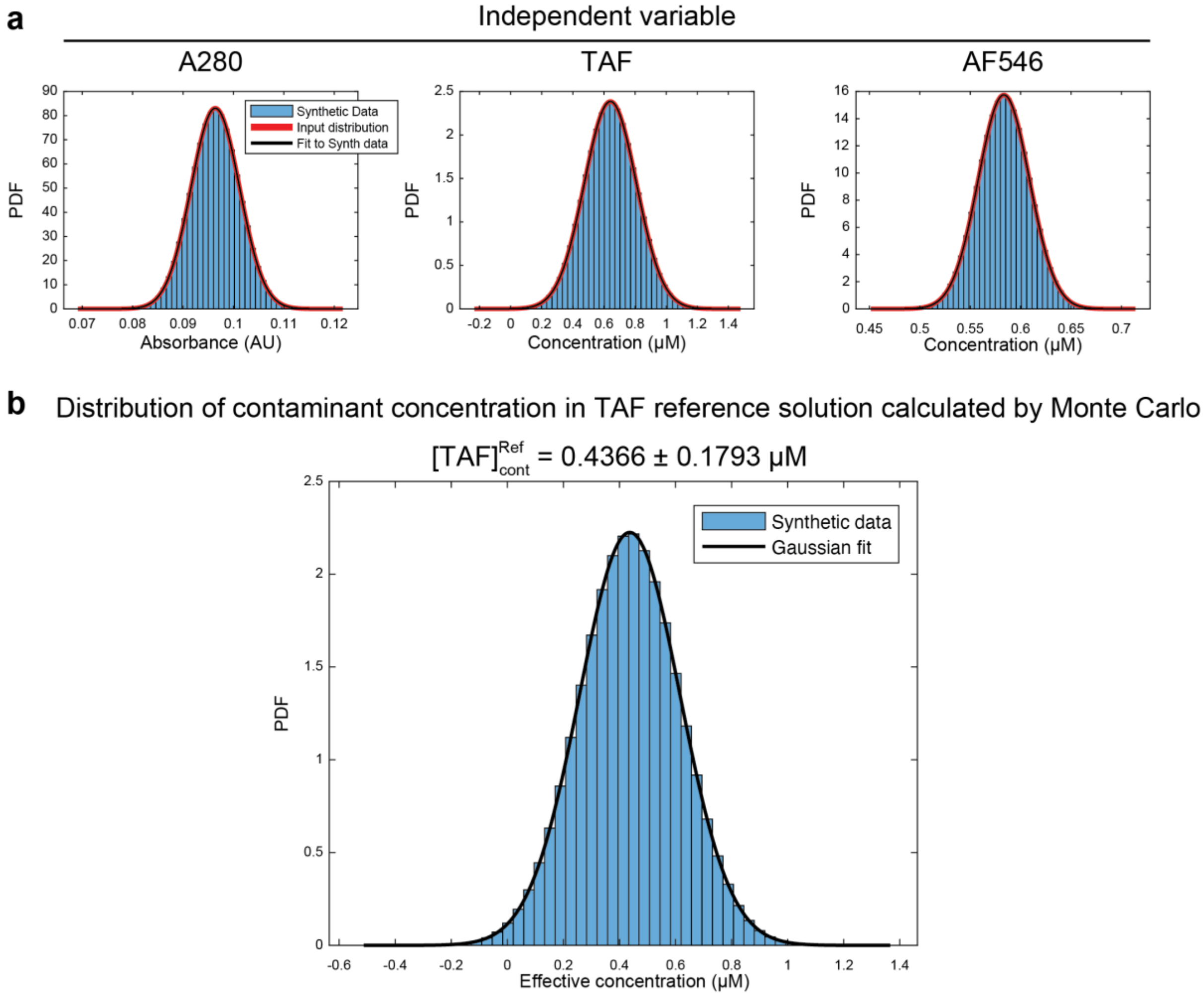

Fig. S13: Estimation of UV-active contaminant abundance in SNAP-TAF15(RBD) stock solution via Monte Carlo simulation

a, Synthetic distributions of 3 non-zero quantities allowed to vary in the calculations: the absorbance at 280 nm of a diluted reference sample from the TAF protein stock (A280, left) as well as the concentrations in that reference sample of SNAP-TAF15(RBD) (TAF, middle) and of Alexa Fluor 546 (AF546, right). Synthetic distributions (blue) were generated via the Monte Carlo method by independently drawing $N_{draw} = 10^7$ values from Gaussian input distributions (red). Black curves represent Gaussian-fits to the synthetic data. Parameters for the input distributions are the mean and standard deviations from measurements in the reference solution of absorbance at 280 nm (left), SNAP-TAF15(RBD) concentration via mass spectrometry (middle), and dye concentration estimates derived from absorption measurements at 554 nm (right). We find that the synthetic distributions are generally well-described as Gaussian. **b**, Concentration distribution of an effective contaminant species calculated from

$$c_{TAF-cont}^{Ref} = \frac{A_{280} - \varepsilon_{TAF} c_{TAF}^{Ref} - \varepsilon_{AF546} c_{AF546}^{Ref}}{\varepsilon_{TAF-cont}}$$

using the synthetic distributions from **a** and setting the extinction coefficient for the contaminant equal to that of SNAP-TAF15(RBD). Black line is a Gaussian fit to the resulting distribution (blue), and generally describes the distributions very well. We estimate the concentration of an effective contaminant species in the reference solution as the mean ± standard deviation of the synthetic distribution: 0.4366 ± 0.1793 μM.

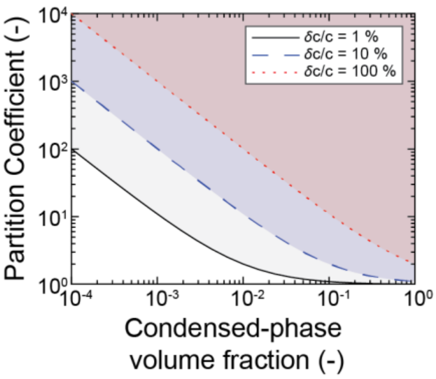

Fig. S14: Minimum partition coefficient resolvable by ATRI

a, The minimum partition coefficient P_{min} for which the concentration of a species in the dilute phase c^I is measurably different from its total average concentration in the system $\bar{c}$ depends on the relative precision $\delta c/c$ of the dilute-phase detection strategy. For a given $\delta c/c$, P_{min} decreases as the volume fraction of the condensed phase increases. In this diagram, curves show the minimum partition coefficient for which partitioning of an arbitrary species would be detectable in a system as a function of condensed-phase volume fraction for $\delta c/c$ = 1% (solid black), 10% (blue dashed), and 100 % (red dotted). The shading denotes that all partition coefficients above the corresponding lower-limit are detectable.

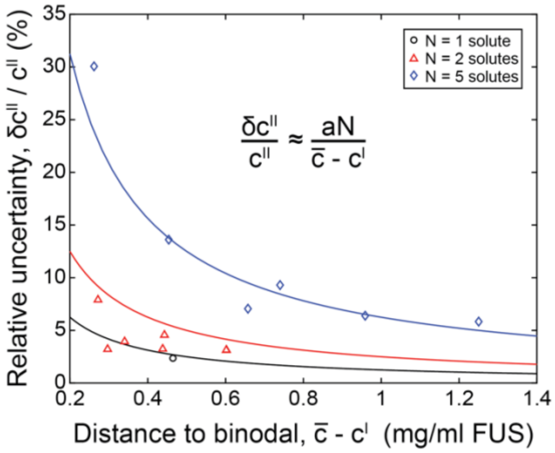

Fig. S15: Scaling analysis of dense-phase uncertainty in multi-component systems
The relative uncertainty in the dense-phase concentration of FUS, $\delta c^{II}/c^{II}$, is shown here as a function of the difference between the system average concentration and the dilute-phase concentration, $\bar{c} - c^{I}$, as the number of macromolecular solutes varies. The N = 1 datapoint corresponds to an effective binary mixture of FUS and buffer. The data for N = 2 solutes are from the FUS/RNA ternary system (**Fig. 4**). For the 5-solute system data containing FL-FUS, MBP-FUS, shortFUS, TAF15(RBD), and RNA (**Fig. 5**), the plotted concentrations refer to FL-FUS. In each case, we find that the relative uncertainty in the dense-phase concentration decreases as the distance into the two-phase region increases. For a given distance into the two-phase region, we also find that the relative dense-phase uncertainty increases with the number of solutes resolved. A global fit to these data (solid curves) to the scaling ansatz (inset) captures the trends reasonably well and yields a value for the proportionality constant of a = 0.0125.

SUPPLEMENTARY TABLES

Table S1: Compositions of biomolecular condensates in vitro.

Construct	Conditions (T in °C, [salt], pH)	c_{cond} mg/mL[a]	Protein Volume Fraction[b]	Partition Coefficient	Method
PGL3	(25.0, 75 mM, 7.4)	87.0 ± 1.7	0.0649 ± 0.0013	n.d.	QPI
PGL3	(21.5, 87 mM, 7.4)	99.2 ± 5.9	0.0740 ± 0.0044	n.d.	ODT
PGL3-mEGFP	(21.5, 87 mM, 7.4)	113.2 ± 8.6	0.0844 ± 0.0064	n.d.	ODT
SNAP-TAF15(RBD)	(37.0, 100 mM, 7.4)	477.1 ± 13.7	0.3400 ± 0.0098	3850[c]	QPI
FUS-mEGFP	(21.0, 150 mM, 7.4)	337.3 ± 8.2	0.2395 ± 0.0058	860[d]	QPI

[a] Uncertainty represents standard deviation from a population of at least 100 individual condensates
[b] Fraction of the condensed phase volume occupied by protein, $\phi \equiv c_{Cond}\bar{v}$; $\bar{v} \approx 0.75$ mL/g for PGL3 constructs and $\bar{v} \approx 0.71$ mL/g for TAF15 and FUS constructs
[c] $c_{Dil} = 1.97 \pm 0.09$ µM, $M_w = 62.92$ kDa; [55]
[d] $c_{Dil} = 4.87 \pm 0.48$ µM, $M_w = 80.38$ kDa; [55]

Table S2: Peptides for MS Western quantification by the method of PRM

N	Peptide sequence	*m/z*		Retention time window, min
		light	heavy	
GFP				
1	FSVSGEGEGDATYGK	752.3335	755.3444	11 - 21
2	FEGDTLVNR	525.7644	530.7692	12 - 23
SNAP				
1	TTLDSPLGK	466.2584	469.2683	12 - 22
2	FGEVISYSHLAALAGNPAATAAVK	786.7533	788.7599	23 - 33
3	VVQGDLDVGGYEGGLAVK	888.4623	891.4721	18 - 28
BSA				
1	DAFLGSFLYEYSR	784.3750	789.3799	25 - 35
2	HLVDEPQNLIK	653.3617	656.3725	15 - 24
3	LGEYGFQNALIVR	740.4014	745.4062	20 - 30
4	LVNELTEFAK	582.319	585.3298	18 - 25
5	YLYEIAR	464.2504	469.2552	15 - 24

Table S3: Protein expression constructs used

**Plasmid name**	**Gene**	**N-term tag**	**C-term tag**	**Origin**
SS02B	PGL-3	-	6xHis-mEGFP	[14]
TH1166	TAF15 RBD 181-589	SNAP	-	[55]
TH1163	TAF15 RBD 181-589	mEGFP	-	Gift from Jie Wang
TH0951	FUS	-	mEGFP	[56]

SUPPLEMENTARY LISTS

List S1: Protein sequences used

Color key:
Main protein sequence
TEV protease recognition sequences (cleave between Q | S)
Precission protease recognition sequences (cleave between Q | GP)
Linkers
6xHis-tag
mEGFP-tag
SNAP-tag
MBP-tag

>> PGL-3-6xHis-mEGFP

MEANKRQIVEVDGIKSYFFPHLAHYLASNDELLVNNIAQANKLAAFVLGATDKRPSNEEIAE
MILPNDSSAYVLAAGMDVCLILGDDFRPKFDSGAEKLSQLGQAHDLAPIIDDEKKISMLARK
TKLKKSNDAKILQVLLKVLGAEEAEEKFVELSELSSALDLDFDVYVLAKLLGFASEELQEEI
EIIRDNVTDAFEACKPLLKKLMIEGPKIDSVDPFTQLLLTPQEESIEKAVSHIVARFEEASA
VEDDESLVLKSQLGYQLIFLVVRSLADGKRDASRTIQSLMPSSVRAEVFPGLQRSVFKSAVF
LASHIIQVFLGSMKSFEDWAFVGLAEDLESTWRRRAIAELLKKFRISVLEQCFSQPIPLLPQ
SELNNETVIENVNNALQFALWITEFYGSESEKKSLNQLQFLSPKSNLLVDSFKKFAQGLDS
KDHVNRIIESLEKSSSSEPSATAKQTTTSNGPTTVSTAAQVVTVEKMPFSRQTIPCEGTDLA
NVLNSAKIIGESVTVAAHDVIPEKLNAEKNDNTPSTASPVQFSSDGWDSPTKSVALPPKIST
LEEEQEEDTTITKVSPQPQERTGTAWGSGDATPVPLATPVNEYKVSGFGAAPVASGFGQFAS
SNGTSGRGSYGGGRGGDRGGRGAYGGDRGRGGSGDGSRGYRGGDRGGRGSYGEGSRGYQGGR
AGFFGGSRGGSSRENLYFQSSAHHHHHHVMVSKGEELFTGVVPILVELDGDVNGHKFSVSG
EGEGDATYGKLTLKFICTTGKLPVPWPTLVTTLTYGVQCFSRYPDHMKQHDFFKSAMPEGYV
QERTIFFKDDGNYKTRAEVKFEGDTLVNRIELKGIDFKEDGNILGHKLEYNYNSHNVYIMAD
KQKNGIKVNFKIRHNIEDGSVQLADHYQQNTPGDGPVLLPDNHYLSTQSALSKDPNEKRDHM
VLKEFVTAAGITLGMDELYKGA

>> PGL-3 untagged
(obtained by treatment of preceding construct with TEV protease)

MEANKRQIVEVDGIKSYFFPHLAHYLASNDELLVNNIAQANKLAAFVLGATDKRPSNEEIAE
MILPNDSSAYVLAAGMDVCLILGDDFRPKFDSGAEKLSQLGQAHDLAPIIDDEKKISMLARK
TKLKKSNDAKILQVLLKVLGAEEAEEKFVELSELSSALDLDFDVYVLAKLLGFASEELQEEI
EIIRDNVTDAFEACKPLLKKLMIEGPKIDSVDPFTQLLLTPQEESIEKAVSHIVARFEEASA
VEDDESLVLKSQLGYQLIFLVVRSLADGKRDASRTIQSLMPSSVRAEVFPGLQRSVFKSAVF
LASHIIQVFLGSMKSFEDWAFVGLAEDLESTWRRRAIAELLKKFRISVLEQCFSQPIPLLPQ
SELNNETVIENVNNALQFALWITEFYGSESEKKSLNQLQFLSPKSNLLVDSFKKFAQGLDS
KDHVNRIIESLEKSSSSEPSATAKQTTTSNGPTTVSTAAQVVTVEKMPFSRQTIPCEGTDLA
NVLNSAKIIGESVTVAAHDVIPEKLNAEKNDNTPSTASPVQFSSDGWDSPTKSVALPPKIST
LEEEQEEDTTITKVSPQPQERTGTAWGSGDATPVPLATPVNEYKVSGFGAAPVASGFGQFAS
SNGTSGRGSYGGGRGGDRGGRGAYGGDRGRGGSGDGSRGYRGGDRGGRGSYGEGSRGYQGGR
AGFFGGSRGGSSRENLYFQ

>> MBP-FUS-mEGFP-6xHis

MKIEEGKLVIWINGDKGYNGLAEVGKKFEKDTGIKVTVEHPDKLEEKFPQVAATGDGPDIIF
WAHDRFGGYAQSGLLAEITPDKAFQDKLYPFTWDAVRYNGKLIAYPIAVEALSLIYNKDLLP
NPPKTWEEIPALDKELKAKGKSALMFNLQEPYFTWPLIAADGGYAFKYENGKYDIKDVGVDN
AGAKAGLTFLVDLIKNKHMNADTDYSIAEAAFNKGETAMTINGPWAWSNIDTSKVNYGVTVL
PTFKGQPSKPFVGVLSAGINAASPNKELAKEFLENYLLTDEGLEAVNKDKPLGAVALKSYEE
ELVKDPRIAATMENAQKGEIMPNIPQMSAFWYAVRTAVINAASGRQTVDEALKDAQTNSSSN
NNNNNNNNNSSGRLEVLFQGPAAAMASNDYTQQATQSYGAYPTQPGQGYSQQSSQPYGQQSY
SGYSQSTDTSGYGQSSYSSYGQSQNTGYGTQSTPQGYGSTGGYGSSQSSQSSYGQQSSYPGY
GQQPAPSSTSGSYGSSSQSSSYGQPQSGSYSQQPSYGGQQQSYGQQQSYNPPQGYGQQNQYN
SSSGGGGGGGGGNYGQDQSSMSSGGGSGGGYGNQDQSGGGGSGGYGQQDRGGRGRGGSGGG
GGGGGGGYNRSSGGYEPRGRGGGRGGRGGMGGSDRGGFNKFGGPRDQGSRHDSEQDNSDNNT
IFVQGLGENVTIESVADYFKQIGIIKTNKKTGQPMINLYTDRETGKLKGEATVSFDDPPSAK
AAIDWFDGKEFSGNPIKVSFATRRADFNRGGGNGRGGRGRGGPMGRGGYGGGGSGGGGRGGF
PSGGGGGGGQQRAGDWKCPNPTCENMNFSWRNECNQCKAPKPDGPGGGPGGSHMGGNYGDDR
RGGRGGYDRGGYRGRGGDRGGFRGGRGGGDRGGFGPGKMDSRGEHRQDRRERPYGAPGSAGS
AAGSGMVSKGEELFTGVVPILVELDGDVNGHKFSVSGEGEGDATYGKLTLKFICTTGKLPVP
WPTLVTTLTYGVQCFSRYPDHMKQHDFFKSAMPEGYVQERTIFFKDDGNYKTRAEVKFEGDT
LVNRIELKGIDFKEDGNILGHKLEYNYNSHNVYIMADKQKNGIKVNFKIRHNIEDGSVQLAD
HYQQNTPIGDGPVLLPDNHYLSTQSKLSKDPNEKRDHMVLLEFVTAAGITLGMDELYKLEVL
FQGPGSSHHHHHHSG

>> FUS-mEGFP
(obtained by cleavage of the preceding construct with Precission protease)

GPAAAMASNDYTQQATQSYGAYPTQPGQGYSQQSSQPYGQQSYSGYSQSTDTSGYGQSSYSS
YGQSQNTGYGTQSTPQGYGSTGGYGSSQSSQSSYGQQSSYPGYGQQPAPSSTSGSYGSSSQS
SSYGQPQSGSYSQQPSYGGQQQSYGQQQSYNPPQGYGQQNQYNSSSGGGGGGGGGNYGQDQ
SSMSSGGGSGGGYGNQDQSGGGGSGGYGQQDRGGRGRGGSGGGGGGGGGGYNRSSGGYEPRG
RGGGRGGRGGMGGSDRGGFNKFGGPRDQGSRHDSEQDNSDNNTIFVQGLGENVTIESVADYF
KQIGIIKTNKKTGQPMINLYTDRETGKLKGEATVSFDDPPSAKAAIDWFDGKEFSGNPIKVS
FATRRADFNRGGGNGRGGRGRGGPMGRGGYGGGGSGGGGRGGFPSGGGGGGGQQRAGDWKCP
NPTCENMNFSWRNECNQCKAPKPDGPGGGPGGSHMGGNYGDDRRGGRGGYDRGGYRGRGGDR
GGFRGGRGGGDRGGFGPGKMDSRGEHRQDRRERPYGAPGSAGSAAGSGMVSKGEELFTGVVP
ILVELDGDVNGHKFSVSGEGEGDATYGKLTLKFICTTGKLPVPWPTLVTTLTYGVQCFSRYP
DHMKQHDFFKSAMPEGYVQERTIFFKDDGNYKTRAEVKFEGDTLVNRIELKGIDFKEDGNIL
GHKLEYNYNSHNVYIMADKQKNGIKVNFKIRHNIEDGSVQLADHYQQNTPIGDGPVLLPDNH
YLSTQSKLSKDPNEKRDHMVLLEFVTAAGITLGMDELYKLEVLFQ

>> SNAP-TAF15(RBD) (residues 181-589)

GPMDKDCEMKRTTLDSPLGKLELSGCEQGLHRIIFLGKGTSAADAVEVPAPAAVLGGPEPLM
QATAWLNAYFHQPEAIEEFPVPALHHPVFQQESFTRQVLWKLLKVVKFGEVISYSHLAALAG
NPAATAAVKTALSGNPVPILIPCHRVVQGDLDVGGYEGGLAVKEWLLAHEGHRLGKPGLGGS
SSGRENLYFQGAAAGRGRGGYDKDGRGPMTGSSGGDRGGFKNFGGHRDYGPRTDADSESDNS
DNNTIFVQGLGEGVSTDQVGEFFKQIGIIKTNKKTGKPMINLYTDKDTGKPKGEATVSFDDP
PSAKAAIDWFDGKEFHGNIIKVSFATRRPEFMRGGGSGGGRRGRGGYRGRGGFQGRGGDPKS
GDWVCPNPSCGNMNFARRNSCNQCNEPRPEDSRPSGGDFRGRGYGGERGYRGRGGRGGDRGG
YGGDRSGGGYGGDRSSGGGYSGDRSGGGYGGDRSGGGYGGDRGGGYGGDRGGGYGGDRGGGY
GGDRGGYGGDRGGGYGGDRGGYGGDRGGYGGDRGGYGGDRGGYGGDRSRGGYGGDRGGGSGY
GGDRSGGYGGDRSGGGYGGDRGGGYGGDRGGYGGKMGGRNDYRNDQRNRPYGAP

>> mEGFP-TAF15(RBD) (residues 181-589)

GPMVSKGEELFTGVVPILVELDGDVNGHKFSVSGEGEGDATYGKLTLKFICTTGKLPVPWPT
LVTTLTYGVQCFSRYPDHMKQHDFFKSAMPEGYVQERTIFFKDDGNYKTRAEVKFEGDTLVN
RIELKGIDFKEDGNILGHKLEYNYNSHNVYIMADKQKNGIKVNFKIRHNIEDGSVQLADHYQ
QNTPIGDGPVLLPDNHYLSTQSKLSKDPNEKRDHMVLLEFVTAAGITLGMDELYKGSSSGRE
NLYFQGAAAGRGRGGYDKDGRGPMTGSSGGDRGGFKNFGGHRDYGPRTDADSESDNSDNNTI
FVQGLGEGVSTDQVGEFFKQIGIIKTNKKTGKPMINLYTDKDTGKPKGEATVSFDDPPSAKA
AIDWFDGKEFHGNIIKVSFATRRPEFMRGGGSGGGRRGRGGYRGRGGFQGRGGDPKSGDWVC
PNPSCGNMNFARRNSCNQCNEPRPEDSRPSGGDFRGRGYGGERGYRGRGGRGGDRGGYGGDR
SGGGYGGDRSSGGGYSGDRSGGGYGGDRSGGGYGGDRGGGYGGDRGGGYGGDRGGGYGGDRG
GYGGDRGGGYGGDRGGYGGDRGGYGGDRGGYGGDRGGYGGDRSRGGYGGDRGGGSGYGGDRS
GGYGGDRSGGGYGGDRGGGYGGDRGGYGGKMGGRNDYRNDQRNRPYGAP

>> BSA (Uniprot ID P02769, FT CHAIN residues 25-607)

DTHKSEIAHRFKDLGEEHFKGLVLIAFSQYLQQCPFDEHVKLVNELTEFAKTCVADESHAGC
EKSLHTLFGDELCKVASLRETYGDMADCCEKQEPERNECFLSHKDDSPDLPKLKPDPNTLCD
EFKADEKKFWGKYLYEIARRHPYFYAPELLYYANKYNGVFQECCQAEDKGACLLPKIETMRE
KVLASSARQRLRCASIQKFGERALKAWSVARLSQKFPKAEFVEVTKLVTDLTKVHKECCHGD
LLECADDRADLAKYICDNQDTISSKLKECCDKPLLEKSHCIAEVEKDAIPENLPPLTADFAE
DKDVCKNYQEAKDAFLGSFLYEYSRRHPEYAVSVLLRLAKEYEATLEECCAKDDPHACYSTV
FDKLKHLVDEPQNLIKQNCDQFEKLGEYGFQNALIVRYTRKVPQVSTPTLVEVSRSLGKVGT
RCCTKPESERMPCTEDYLSLILNRLCVLHEKTPVSEKVTKCCTESLVNRRPCFSALTPDETY
VPKAFDEKLFTFHADICTLPDTEKQIKKQTALVELLKHKPKATEEQLKTVMENFVAFVDKCC
AADDKEACFAVEGPKLVVSTQTALA

SUPPLEMENTARY REFERENCES

1. Broide, M. L., Berland, C. R., Pande, J., Ogun, O. O. & Benedek, G. B. Binary-liquid phase separation of lens protein solutions. *Proc. Natl. Acad. Sci.* **88**, 5660–5664 (1991).
2. Zhang, F. *et al.* Charge-controlled metastable liquid–liquid phase separation in protein solutions as a universal pathway towards crystallization. *Soft Matter* **8**, 1313–1316 (2012).
3. Li, L. *et al.* Phase Behavior and Salt Partitioning in Polyelectrolyte Complex Coacervates. *Macromolecules* (2018) doi:10.1021/acs.macromol.8b00238.
4. Küffner, A. M. *et al.* Acceleration of an Enzymatic Reaction in Liquid Phase Separated Compartments Based on Intrinsically Disordered Protein Domains. *ChemSystemsChem* **2**, e2000001 (2020).
5. Avni, A., Joshi, A., Walimbe, A., Pattanashetty, S. G. & Mukhopadhyay, S. Single-droplet surface-enhanced Raman scattering decodes the molecular determinants of liquid-liquid phase separation. *Nat. Commun.* **13**, 4378 (2022).
6. Conrad, N., Chang, G., Fygenson, D. K. & Saleh, O. A. Emulsion imaging of a DNA nanostar condensate phase diagram reveals valence and electrostatic effects. *J. Chem. Phys.* **157**, 234203 (2022).
7. Fritsch, A. W., Iglesias-Artola, J. M. & Hyman, A. A. inPhase — A simple, accurate and fast approach to determine phase diagrams of protein condensates. 2024.10.02.616352 Preprint at https://doi.org/10.1101/2024.10.02.616352 (2024).
8. Klein, I. A. *et al.* Partitioning of cancer therapeutics in nuclear condensates. *Science* **368**, 1386–1392 (2020).
9. Hong, Y. *et al.* Label-Free Quantitative Analysis of Coacervates via 3D Phase Imaging. *Adv. Opt. Mater.* **9**, 2100697 (2021).
10. Kim, T. *et al.* RNA-mediated demixing transition of low-density condensates. *Nat. Commun.* **14**, 2425 (2023).
11. Brady, J. P. *et al.* Structural and hydrodynamic properties of an intrinsically disordered region of a germ cell-specific protein on phase separation. *Proc. Natl. Acad. Sci.* **114**, E8194–E8203 (2017).
12. Martin, E. W. *et al.* Valence and patterning of aromatic residues determine the phase behavior of prion-like domains. *Science* **367**, 694–699 (2020).
13. Bremer, A. *et al.* Deciphering how naturally occurring sequence features impact the phase behaviours of disordered prion-like domains. *Nat. Chem.* **14**, 196–207 (2022).
14. Saha, S. *et al.* Polar Positioning of Phase-Separated Liquid Compartments in Cells Regulated by an mRNA Competition Mechanism. *Cell* **166**, 1572-1584.e16 (2016).
15. Zhao, H., Brown, P. H. & Schuck, P. On the Distribution of Protein Refractive Index Increments. *Biophys. J.* **100**, 2309–2317 (2011).
16. Heller, W. Remarks on Refractive Index Mixture Rules. *J. Phys. Chem.* **69**, 1123–1129 (1965).
17. Mayerhöfer, T. G., Dabrowska, A., Schwaighofer, A., Lendl, B. & Popp, J. Beyond Beer's Law: Why the Index of Refraction Depends (Almost) Linearly on Concentration. *ChemPhysChem* **21**, 707–711 (2020).
18. Bakker, H. J. Reaction-field model for the dielectric response of mixtures. *J. Chem. Phys.* **153**, 054503 (2020).
19. Zhuang, B., Ramanauskaite, G., Koa, Z. Y. & Wang, Z.-G. Like dissolves like: A first-principles theory for predicting liquid miscibility and mixture dielectric constant. *Sci. Adv.* **7**, eabe7275 (2021).
20. Barer, R. & Tkaczyk, S. Refractive Index of Concentrated Protein Solutions. *Nature* **173**, 821–822 (1954).

21. Barer, R. & Joseph, S. Refractometry of Living Cells: Part I. Basic Principles. *J. Cell Sci.* **s3-95**, 399–423 (1954).
22. Popescu, G. *et al.* Optical imaging of cell mass and growth dynamics. *Am. J. Physiol.-Cell Physiol.* **295**, C538–C544 (2008).
23. Biswas, A., Kim, K., Cojoc, G., Guck, J. & Reber, S. The Xenopus spindle is as dense as the surrounding cytoplasm. *Dev. Cell* **56**, 967-975.e5 (2021).
24. Park, J., Park, J., Lim, H. & Kim, H.-Y. Shape of a large drop on a rough hydrophobic surface. *Phys. Fluids* **25**, 022102 (2013).
25. Iserman, C. *et al.* Condensation of Ded1p Promotes a Translational Switch from Housekeeping to Stress Protein Production. *Cell* **181**, 818-831.e19 (2020).
26. Mittasch, M. *et al.* Non-invasive perturbations of intracellular flow reveal physical principles of cell organization. *Nat. Cell Biol.* **20**, 344–351 (2018).
27. Fritsch, A. W. *et al.* Local thermodynamics govern formation and dissolution of Caenorhabditis elegans P granule condensates. *Proc. Natl. Acad. Sci.* **118**, e2102772118 (2021).
28. Schlüßler, R. *et al.* Correlative all-optical quantification of mass density and mechanics of subcellular compartments with fluorescence specificity. *eLife* **11**, e68490 (2022).
29. Sarimov, R. M., Matveyeva, T. A., Vasin, A. L. & Binhi, V. N. Changes in the refractive index of a solution during proteolysis of bovine serum albumin with pepsin. *Biophysics* **62**, 177–181 (2017).
30. Khago, D., Bierma, J. C., Roskamp, K. W., Kozlyuk, N. & Martin, R. W. Protein refractive index increment is determined by conformation as well as composition. *J. Phys. Condens. Matter* **30**, 435101 (2018).
31. Jawerth, L. M. *et al.* Protein condensates as aging Maxwell fluids. *Science* **370**, 1317–1323 (2020).
32. Alshareedah, I. *et al.* Sequence-specific interactions determine viscoelasticity and ageing dynamics of protein condensates. *Nat. Phys.* **20**, 1482–1491 (2024).
33. Roy, H. L. & Rios, P. D. L. Microscopic model for aging of biocondensates. Preprint at https://doi.org/10.48550/arXiv.2407.21710 (2024).
34. Artifacts of light. *Nat. Methods* **10**, 1135–1135 (2013).
35. Magidson, V. & Khodjakov, A. Chapter 23 - Circumventing Photodamage in Live-Cell Microscopy. in *Methods in Cell Biology* (eds. Sluder, G. & Wolf, D. E.) vol. 114 545–560 (Academic Press, 2013).
36. Wäldchen, S., Lehmann, J., Klein, T., van de Linde, S. & Sauer, M. Light-induced cell damage in live-cell super-resolution microscopy. *Sci. Rep.* **5**, 15348 (2015).
37. Icha, J., Weber, M., Waters, J. C. & Norden, C. Phototoxicity in live fluorescence microscopy, and how to avoid it. *BioEssays* **39**, 1700003 (2017).
38. Foote, C. S. Mechanisms of Photosensitized Oxidation. *Science* **162**, 963–970 (1968).
39. Rubinstein, M. & Colby, R. H. *Polymer Physics*. (Oxford University Press, New York, NY, 2003).
40. Toll, J. S. Causality and the Dispersion Relation: Logical Foundations. *Phys. Rev.* **104**, 1760–1770 (1956).
41. Fitzgerald, P. D. Numerical Approximation of Kramers-Kronig Relations to Transform Discretized Absorption Data. Preprint at https://doi.org/10.48550/arXiv.2012.02369 (2020).
42. Jordi Taltavull, M. The Uncertain Limits Between Classical and Quantum Physics: Optical Dispersion and Bohr's Atomic Model. *Ann. Phys.* **530**, 1800104 (2018).
43. Shevchenko, A., Tomas, H., Havli, J., Olsen, J. V. & Mann, M. In-gel digestion for mass spectrometric characterization of proteins and proteomes. *Nat. Protoc.* **1**, 2856–2860 (2006).

44. Kumar, M. *et al.* MS Western, a Method of Multiplexed Absolute Protein Quantification is a Practical Alternative to Western Blotting. *Mol. Cell. Proteomics* **17**, 384–396 (2018).
45. Müller, P., Schürmann, M., Girardo, S., Cojoc, G. & Guck, J. Accurate evaluation of size and refractive index for spherical objects in quantitative phase imaging. *Opt. Express* **26**, 10729–10743 (2018).
46. Stanley, C. B., Hong, H. & Strey, H. H. DNA Cholesteric Pitch as a Function of Density and Ionic Strength. *Biophys. J.* **89**, 2552–2557 (2005).
47. Scheff, D. R. *et al.* Tuning shape and internal structure of protein droplets via biopolymer filaments. *Soft Matter* **16**, 5659–5668 (2020).
48. Porus, M., Labbez, C., Maroni, P. & Borkovec, M. Adsorption of monovalent and divalent cations on planar water-silica interfaces studied by optical reflectivity and Monte Carlo simulations. *J. Chem. Phys.* **135**, 064701 (2011).
49. *CRC Handbook of Chemistry and Physics*. (CRC Press, Boca Raton, 2014).
50. McMeekin, T. L., Wilensky, M. & Groves, M. L. Refractive indices of proteins in relation to amino acid composition and specific volume. *Biochem. Biophys. Res. Commun.* **7**, 151–156 (1962).
51. Slavov, N. Scaling Up Single-Cell Proteomics. *Mol. Cell. Proteomics* **21**, 100179 (2022).
52. Jawerth, L. M. *et al.* Salt-Dependent Rheology and Surface Tension of Protein Condensates Using Optical Traps. *Phys. Rev. Lett.* **121**, 258101 (2018).
53. Malitson, I. H. Interspecimen Comparison of the Refractive Index of Fused Silica*,†. *JOSA* **55**, 1205–1209 (1965).
54. Cheong, F. C., Xiao, K., Pine, D. J. & Grier, D. G. Holographic characterization of individual colloidal spheres' porosities. *Soft Matter* **7**, 6816–6819 (2011).
55. Wang, J. *et al.* A Molecular Grammar Governing the Driving Forces for Phase Separation of Prion-like RNA Binding Proteins. *Cell* **174**, 688-699.e16 (2018).
56. Patel, A. *et al.* A Liquid-to-Solid Phase Transition of the ALS Protein FUS Accelerated by Disease Mutation. *Cell* **162**, 1066–1077 (2015).
