# Supplementary material for: A label-free method for measuring the composition of multicomponent biomolecular condensates
Source: Nat Chem. 2025 Sep 3;17(12):1891–902. doi: 10.1038/s41557-025-01928-3 (PMC12669041; doi:10.1038/s41557-025-01928-3)
Supplement: Supplementary file 2 — Reporting Summary [file 41557_2025_1928_MOESM2_ESM.pdf]

nature portfolio

Corresponding author(s): Patrick M. McCall, Jan Brugués

Last updated by author(s): Jul 12, 2025

Reporting Summary

Nature Portfolio wishes to improve the reproducibility of the work that we publish. This form provides structure for consistency and transparency in reporting. For further information on Nature Portfolio policies, see our Editorial Policies and the Editorial Policy Checklist.

Statistics

For all statistical analyses, confirm that the following items are present in the figure legend, table legend, main text, or Methods section.

n/a	Confirmed	
☐	☒	The exact sample size (*n*) for each experimental group/condition, given as a discrete number and unit of measurement
☐	☒	A statement on whether measurements were taken from distinct samples or whether the same sample was measured repeatedly
☐	☒	The statistical test(s) used AND whether they are one- or two-sided<br>*Only common tests should be described solely by name; describe more complex techniques in the Methods section.*
☒	☐	A description of all covariates tested
☒	☐	A description of any assumptions or corrections, such as tests of normality and adjustment for multiple comparisons
☐	☒	A full description of the statistical parameters including central tendency (e.g. means) or other basic estimates (e.g. regression coefficient) AND variation (e.g. standard deviation) or associated estimates of uncertainty (e.g. confidence intervals)
☒	☐	For null hypothesis testing, the test statistic (e.g. *F*, *t*, *r*) with confidence intervals, effect sizes, degrees of freedom and *P* value noted<br>*Give P values as exact values whenever suitable.*
☒	☐	For Bayesian analysis, information on the choice of priors and Markov chain Monte Carlo settings
☒	☐	For hierarchical and complex designs, identification of the appropriate level for tests and full reporting of outcomes
☐	☒	Estimates of effect sizes (e.g. Cohen's *d*, Pearson's *r*), indicating how they were calculated

Our web collection on statistics for biologists contains articles on many of the points above.

Software and code

Policy information about availability of computer code

Data collection	Q-PHASE Software v7 for acquisition of quantitative phase images on TESCAN Q-PHASE G1.<br>SophiQ v9.2.415 for acquisition of quantitative phase images on Telight Q-PHASE G2.<br>Custom GUI run with MATLAB 2017b for acquisition of ODT images.<br>Carl Zeiss Zen 2.3 SP1 FP3 (black) v14.0.25.201 for acquisition of confocal fluorescence images.<br>IMPLEN NPOS 4.2g firmware build 14900 for acquisition of UV-Vis spectra.
Data analysis	MATLAB 2017b for ODT image analysis.<br>MATLAB 2021b for analysis of quantitative phase images, confocal fluorescence images, UV-Vis spectra, and refractometry data.<br>Fiji running with ImageJ 2.3.051 and Java 1.8.0_202 for image exploration, contrast-scaling, false-coloring and cropping.

For manuscripts utilizing custom algorithms or software that are central to the research but not yet described in published literature, software must be made available to editors and reviewers. We strongly encourage code deposition in a community repository (e.g. GitHub). See the Nature Portfolio guidelines for submitting code & software for further information.

Data

Policy information about availability of data

All manuscripts must include a data availability statement. This statement should provide the following information, where applicable:

- Accession codes, unique identifiers, or web links for publicly available datasets
- A description of any restrictions on data availability
- For clinical datasets or third party data, please ensure that the statement adheres to our policy

Sequences for the proteins used in this work are provided in the Supplementary Information. Mass spectrometry data are publicly available on the Edmond data repository (https://doi.org/10.17617/3.PCTHKT) hosted by the Max Planck Society. Source data, including the dense-phase and dilute-phase compositions measured for the multi-component protein/RNA systems described in this work, are provided with this paper. Further materials, such as raw microscopy images, are available upon reasonable request to the corresponding authors.

Human research participants

Policy information about studies involving human research participants and Sex and Gender in Research.

Reporting on sex and gender	*Use the terms sex (biological attribute) and gender (shaped by social and cultural circumstances) carefully in order to avoid confusing both terms. Indicate if findings apply to only one sex or gender; describe whether sex and gender were considered in study design whether sex and/or gender was determined based on self-reporting or assigned and methods used. Provide in the source data disaggregated sex and gender data where this information has been collected, and consent has been obtained for sharing of individual-level data; provide overall numbers in this Reporting Summary. Please state if this information has not been collected. Report sex- and gender-based analyses where performed, justify reasons for lack of sex- and gender-based analysis.*
Population characteristics	*Describe the covariate-relevant population characteristics of the human research participants (e.g. age, genotypic information, past and current diagnosis and treatment categories). If you filled out the behavioural & social sciences study design questions and have nothing to add here, write "See above."*
Recruitment	*Describe how participants were recruited. Outline any potential self-selection bias or other biases that may be present and how these are likely to impact results.*
Ethics oversight	*Identify the organization(s) that approved the study protocol.*

Note that full information on the approval of the study protocol must also be provided in the manuscript.

Field-specific reporting

Please select the one below that is the best fit for your research. If you are not sure, read the appropriate sections before making your selection.

☒ Life sciences ☐ Behavioural & social sciences ☐ Ecological, evolutionary & environmental sciences

For a reference copy of the document with all sections, see nature.com/documents/nr-reporting-summary-flat.pdf

Life sciences study design

All studies must disclose on these points even when the disclosure is negative.

Sample size	For bulk measurements of UV-VIS spectra, 3 repeats of each sample were performed for each condition.<br>For bulk refractometry measurements, 5 repeats of each sample were performed for each condition.<br>For individual droplet measurements, the number of fields of view recorded (typically 9-25) was chosen based on an assessment by eye of the typical size and number density of droplets in single FOV for a given experimental condition such that typically several hundred or more individual droplets from that condition would be suitable for further analysis.
Data exclusions	As described in the Methods, individual droplets were excluded from quantitative analysis when they did not meet the physical assumptions of the analysis. Droplets were excluded from QPI analysis if the shape inferred from the phase image was not well-described by a spherical cap, for instance due to irregular wetting of the coverslip or the presence of an additional object in solution directly above the droplet of interest. Similarly, droplets were excluded from ODT analysis if their tomographic reconstructions were strongly impacted by artifacts due to their position near the edge of the field of view. Droplets with volumes smaller than 0.1194 μm^3 (equivalent to fewer than 100 voxels) were also excluded from ODT analysis to avoid misclassification of small segmented regions of background noise as droplets. As samples G and H in Fig. S8 were both prepared in the presence of a small number of polystyrene microspheres with radii near 2 µm, we additionally restricted the ODT analysis of these samples to objects with volumes smaller than 15 µm^3 to ensure that the few microspheres present in our tomograms were excluded. Droplets were excluded from final fluorescence analysis when they were too small relative to the axial point-spread function for the fluorescence intensity estimated from interior pixels to reach the level observed in larger droplets (see also Extended Data Fig. 2). Otherwise, data were not excluded from the analyses.

Replication	All attempts at replication were successful. In particular, we observed similar results upon repeating quantitative phase measurements under similar conditions on different days with polymers within and across batches. The method validation described in Fig 2. as well as the smooth and physically sensible variation we observe in the measured droplet composition upon titrations of buffer composition or temperature give us confidence in reported trends and absolute magnitudes. For multi-component systems, binodals measured on different days were self-consistent.
Randomization	For titration experiments in which individual samples were prepared in parallel and subsequently measured in series, the measurement order was manually shuffled (i.e. not measured in order of monotonically increasing concentration) in order to avoid introducing potential bias from the slightly different sample ages at the time of measurement.
Blinding	Not relevant for this study.

Reporting for specific materials, systems and methods

We require information from authors about some types of materials, experimental systems and methods used in many studies. Here, indicate whether each material, system or method listed is relevant to your study. If you are not sure if a list item applies to your research, read the appropriate section before selecting a response.

Materials & experimental systems

n/a	Involved in the study
☒	☐ Antibodies
☒	☐ Eukaryotic cell lines
☒	☐ Palaeontology and archaeology
☒	☐ Animals and other organisms
☒	☐ Clinical data
☒	☐ Dual use research of concern

Methods

n/a	Involved in the study
☒	☐ ChIP-seq
☒	☐ Flow cytometry
☒	☐ MRI-based neuroimaging
