# Supplementary figures and images for: A label-free method for measuring the composition of multicomponent biomolecular condensates
Source: Nat Chem. 2025 Sep 3;17(12):1891–902. doi: 10.1038/s41557-025-01928-3 (PMC12669041; doi:10.1038/s41557-025-01928-3)

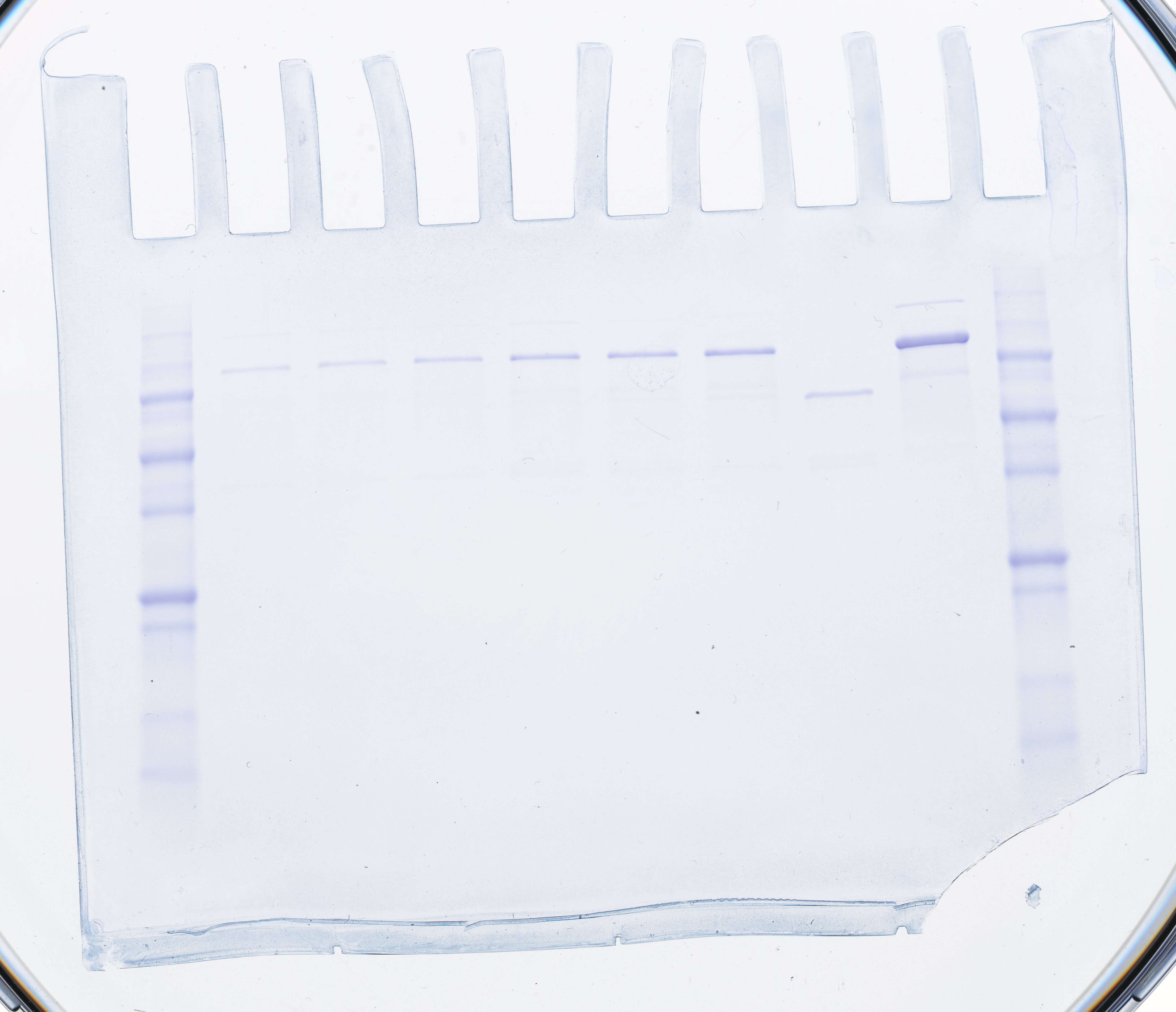

Supplement: Supplementary file 18 — Unprocessed gel image. [file 41557_2025_1928_MOESM18_ESM.jpg]
